# Supplementary material for: Treatment effects of soluble guanylate cyclase modulation on diabetic kidney disease at single-cell resolution
Source: Cell Rep Med. 2023 Apr 5;4(4):100992. doi: 10.1016/j.xcrm.2023.100992 (PMC10140477; doi:10.1016/j.xcrm.2023.100992)
Supplement: Document S1. Figures S1–S14 and Table S1 [file mmc1.pdf]

Cell Reports Medicine, Volume *4*

Supplemental information

Treatment effects of soluble guanylate cyclase modulation on diabetic kidney disease at single-cell resolution

Michael S. Balzer, Mira Pavkovic, Julia Frederick, Amin Abedini, Alexius Freyberger, Julia Vienenkötter, Ilka Mathar, Krystyna Siudak, Frank Eitner, Peter Sandner, Manuel Grundmann, and Katalin Susztak

Supplementary Information

Treatment effects of soluble guanylate cyclase modulation on diabetic kidney disease at single-cell resolution

Michael S. Balzer[1,2,3,4], Mira Pavkovic[5], Julia Frederick[1,2], Amin Abedini[1,2], Alexius Freyberger[5], Julia Vienenkötter[5], Ilka Mathar[5], Krystyna Siudak[5], Frank Eitner[5,6], Peter Sandner[5,7], Manuel Grundmann[5], and Katalin Susztak[1,2,8,9]

[1]Renal, Electrolyte, and Hypertension Division, Department of Medicine, Perelman School of Medicine, University of Pennsylvania, Philadelphia, PA 19104, USA
[2]Institute for Diabetes, Obesity and Metabolism, Perelman School of Medicine, University of Pennsylvania, Philadelphia, PA 19104, USA
[3]Department of Nephrology and Medical Intensive Care, Charité – Universitätsmedizin Berlin, 10117 Berlin, Germany
[4]Berlin Institute of Health at Charité – Universitätsmedizin Berlin, BIH Biomedical Innovation Academy, BIH Charité Clinician Scientist Program, 10117 Berlin, Germany
[5]Bayer AG, Research and Early Development, Pharma Research Center, 42096 Wuppertal, Germany
[6]Division of Nephrology and Clinical Immunology, RWTH Aachen University, 52062 Aachen, Germany
[7]Department of Pharmacology, Hannover Medical School, 30625 Hannover, Germany
[8]Department of Genetics, Perelman School of Medicine, University of Pennsylvania, Philadelphia, PA 19104, USA

[9]Lead contact
Katalin Susztak, MD, PhD
Professor of Medicine
University of Pennsylvania
Perelman School of Medicine
3400 Civic Center Blvd
Smilow Translational Research Center 12-123
Philadelphia, PA 19104
Phone: (215) 898-2009
ksusztak@pennmedicine.upenn.edu

Supplementary Table

Page 2

Supplementary Figures

Pages 3-30

Table S1. Blood pressure effects of sGC activation in the ZSF1 rat. Related to Figure 1.

Treatment	Baseline MAP (mmHg)	Post-application MAP (mmHg)
Vehicle	120.4 ± 4.8	112.6 ± 1.9
Runcaciguat (3 mg/kg)	122.4 ± 4.0	110.7 ± 1.8

Mean arterial blood pressure (MAP) measured in mmHg with telemetric implants in ZSF1 rats treated with either vehicle (n=6) or 3 mg/kg runcaciguat (n=5); data are shown as mean ± SEM registered over 1 hour before the application for baseline (hour -1 to 0) and 5 hours after application (hour +1 to +6).

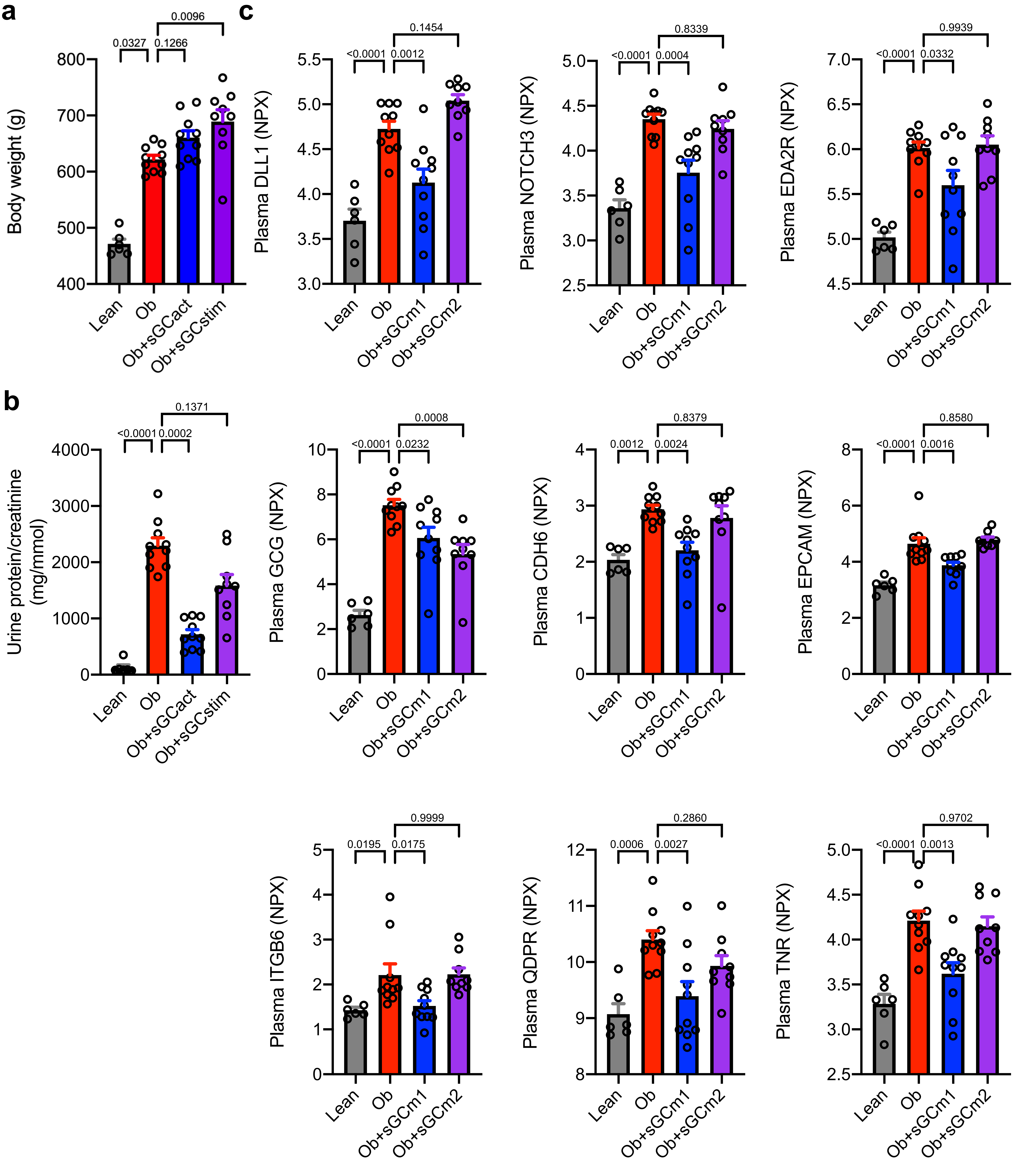
a
b
c
Body weight (g)
Urine protein/creatinine (mg/mmol)
Plasma DLL1 (NPX)
Plasma NOTCH3 (NPX)
Plasma EDA2R (NPX)
Plasma GCG (NPX)
Plasma CDH6 (NPX)
Plasma EPCAM (NPX)
Plasma ITGB6 (NPX)
Plasma QDPR (NPX)
Plasma TNR (NPX)
Lean
Ob
Ob+sGCact
Ob+sGCstim
Ob+sGCm1
Ob+sGCm2
0.0327
0.1266
0.0096
<0.0001
0.0002
0.1371
0.0012
0.1454
0.0004
0.8339
0.0332
0.9939
0.0232
0.0008
0.0024
0.8379
0.0016
0.8580
0.0195
0.0175
0.9999
0.0006
0.0027
0.2860
0.0013
0.9702

Figure S1. Diabetic ZSF1 rats recapitulate phenotypic changes of DKD with marked disease improvement by sGC activators. Related to Figure 1.

(a) Body weight; p values are given for Kruskal-Wallis test (Benjamini, Krieger, Yekutieli-corrected); Ob, Obese.

(b) Urinary protein/creatinine ratio; p values are given for Kruskal-Wallis test (Benjamini, Krieger, Yekutieli-corrected); Ob, Obese.

(c) Explorative proteomics analysis of 92 plasma proteins using a multiplexed proximity extension assay (Olink); p values are given for one-way ANOVA (Benjamini, Krieger, Yekutieli-corrected); NPX, normalized protein expression; Ob, Obese.

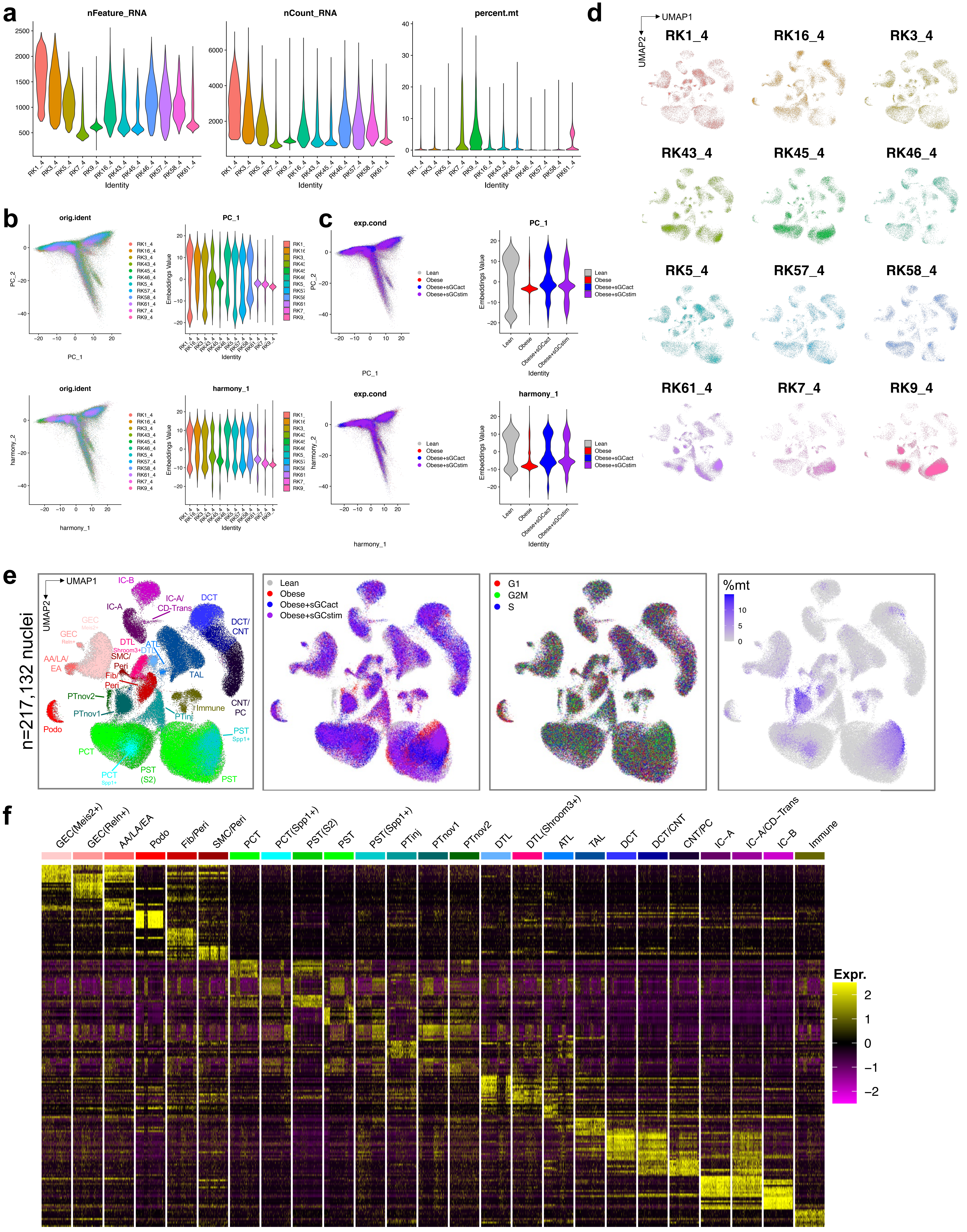
a
nFeature_RNA
nCount_RNA
percent.mt
Identity
RK1_4
RK3_4
RK5_4
RK7_4
RK9_4
RK16_4
RK43_4
RK45_4
RK46_4
RK57_4
RK58_4
RK61_4
b
orig.ident
PC_1
PC_2
harmony_1
harmony_2
Embeddings Value
c
exp.cond
Lean
Obese
Obese+sGCact
Obese+sGCstim
d
UMAP1
UMAP2
RK1_4
RK16_4
RK3_4
RK43_4
RK45_4
RK46_4
RK5_4
RK57_4
RK58_4
RK61_4
RK7_4
RK9_4
e
n=217,132 nuclei
IC-B
IC-A
IC-A/ CD-Trans
DCT
DCT/ CNT
GEC Meis2+
GEC Reln+
AA/LA/ EA
DTL Shroom3+
ATL
DTL
SMC/ Peri
Fib/ Peri
TAL
PTnov2
PTnov1
Immune
PTinj
CNT/ PC
Podo
PST Spp1+
PCT
PCT Spp1+
PST (S2)
PST
G1
G2M
S
%mt
f
GEC(Meis2+)
GEC(Reln+)
AA/LA/EA
Podo
Fib/Peri
SMC/Peri
PCT
PCT(Spp1+)
PST(S2)
PST
PST(Spp1+)
PTinj
PTnov1
PTnov2
DTL
DTL(Shroom3+)
ATL
TAL
DCT
DCT/CNT
CNT/PC
IC-A
IC-A/CD-Trans
IC-B
Immune
Expr.

Figure S2. Single-cell transcriptomic landscape of the diabetic ZSF1 rat. Related to Figure 2.

(a) Violin plots showing number of informative genes (nFeature_RNA), unique molecular identifiers (nCount_RNA), and percentage of mitochondrial genes (percent.mt) per single cell.

(b) Principal component (PC) representation (left) and violin plots of corresponding embeddings values (right) before (top) and after batch correction (bottom) using Harmony, stratified by individual sample.

(c) Same as in **(b)**, stratified by experimental group.

(d) Integrated UMAP corresponding to **Fig. 2a**, stratified by sample.

(e) Integrated UMAP corresponding to **Fig. 2a**, annotated by low-level clustering, experimental group, cell cycle phase, and mitochondrial percentage (%mt), respectively; GEC, glomerular endothelial cell; AA/LA/EA, afferent arteriole/large artery/efferent arteriole; Podo, podocyte; Fib, fibroblast; Peri, pericyte; SMC; smooth muscle cell; PCT, proximal convoluted tubule; PST(S2); proximal straight tubule (segment 2); PTinj, injured proximal tubule; PTnov, novel PT; DTL, descending thin limb; ATL, ascending thin limb; TAL, thick ascending limb; DCT, distal convoluted tubule; CNT, connecting tubule; PC, collecting duct principal cell; IC-A, collecting duct intercalated cell type A; CD-Trans, collecting duct transitional cell; IC-B, collecting duct intercalated cell type B; Immune, immune cell.

(f) Heatmap of top 10 differentially expressed genes for low-level clustering, corresponding to **Fig. 2b**.

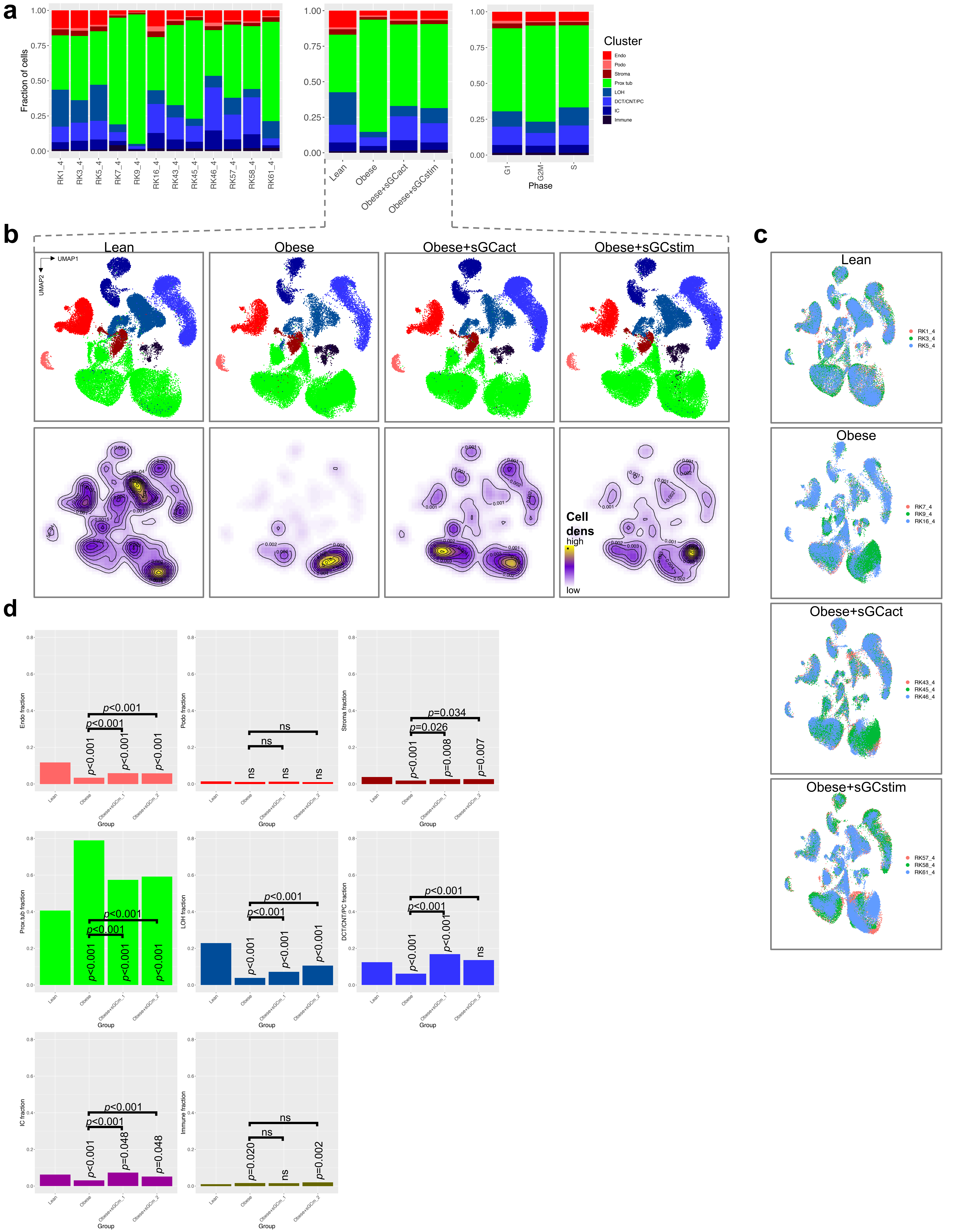

a
Fraction of cells
RK1_4 RK3_4 RK5_4 RK7_4 RK9_4 RK16_4 RK43_4 RK45_4 RK46_4 RK57_4 RK58_4 RK61_4
Lean
Obese
Obese+sGCact
Obese+sGCstim
G1
G2M
S
Phase
Cluster
Endo
Podo
Stroma
Prox tub
LOH
DCT/CNT/PC
IC
Immune
b
Lean
Obese
Obese+sGCact
Obese+sGCstim
UMAP1
UMAP2
Cell dens
high
low
c
Lean
RK1_4
RK3_4
RK5_4
Obese
RK7_4
RK9_4
RK16_4
Obese+sGCact
RK43_4
RK45_4
RK46_4
Obese+sGCstim
RK57_4
RK58_4
RK61_4
d
Endo fraction
Podo fraction
Stroma fraction
Prox.tub fraction
LOH fraction
DCT/CNT/PC fraction
IC fraction
Immune fraction
Group
Lean
Obese
Obese+sGCm_1
Obese+sGCm_2
p<0.001
p=0.034
p=0.026
p=0.008
p=0.007
p=0.048
p=0.020
p=0.002
ns

Figure S3. Cell fraction changes. Related to Figure 2.

(a) Fractions of high-level clustering cell types across samples, experimental groups, and cell cycle phase, respectively; Endo, endothelial cell; Podo, podocyte; Stroma, stromal cell; Prox tub, proximal tubule; LOH, loop of Henle; DCT/CNT/PC, distal convoluted tubule/connecting tubule/collecting duct principal cell; IC, collecting duct intercalated cell; Immune, immune cell.

(b) Integrated UMAP (top) and density plots (bottom) corresponding to **Fig. 2a**, stratified by experimental group.

(c) Integrated UMAP stratified by experimental group and colored by individual sample demonstrates negligible within-group heterogeneity.

(d) Cell fractions of high-level clusters stratified by experimental group. Statistical significance for comparisons was derived using differential proportion analysis, with a mean error of 0.1 over 100,000 iterations; p values are given for comparisons between Lean and (un-)treated Obese groups and between untreated Obese and treated Obese groups, respectively; ns, not significant.

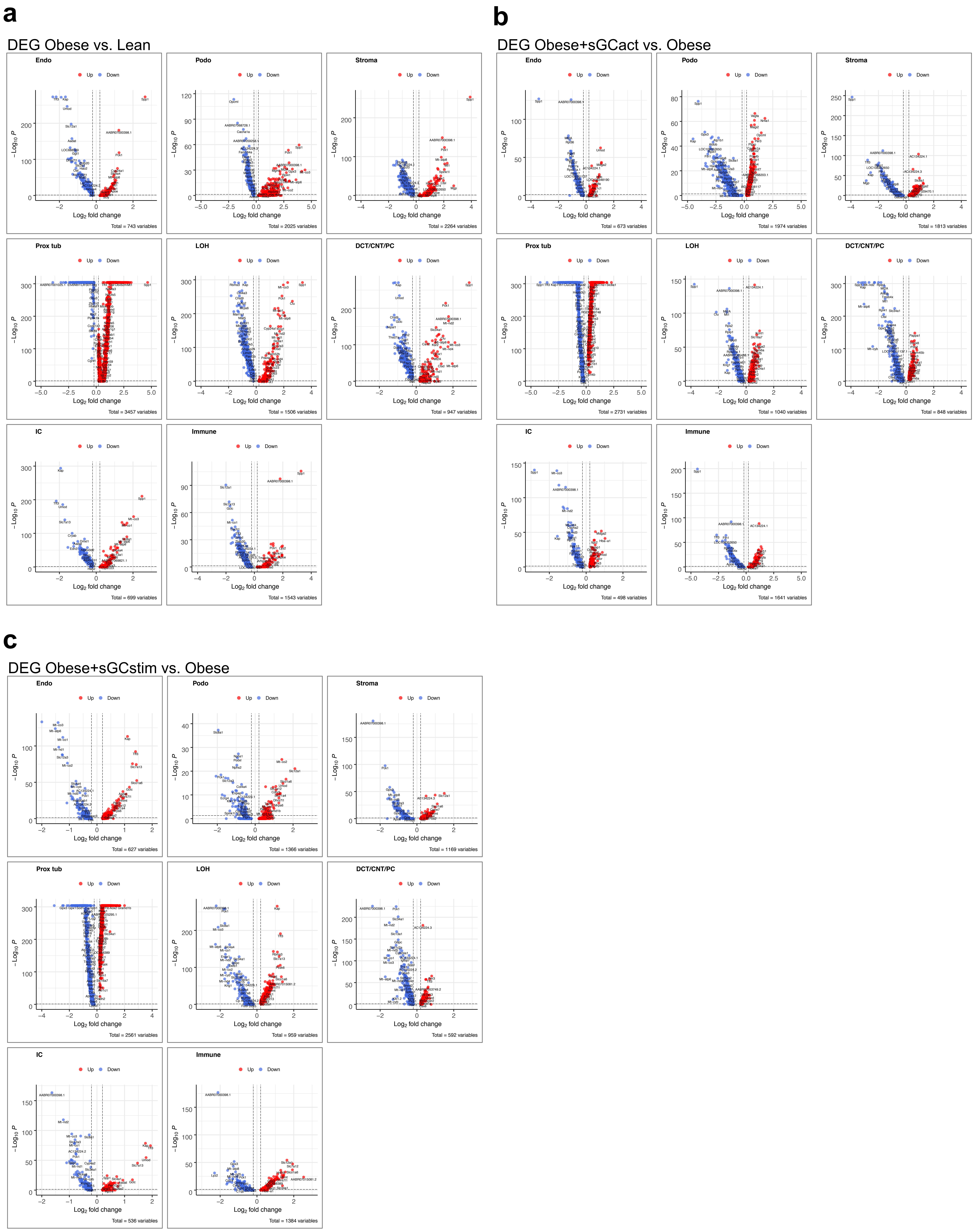
a
DEG Obese vs. Lean
Endo
Total = 743 variables
Podo
Total = 2025 variables
Stroma
Total = 2264 variables
Prox tub
Total = 3457 variables
LOH
Total = 1506 variables
DCT/CNT/PC
Total = 947 variables
IC
Total = 699 variables
Immune
Total = 1543 variables
b
DEG Obese+sGCact vs. Obese
Endo
Total = 673 variables
Podo
Total = 1974 variables
Stroma
Total = 1813 variables
Prox tub
Total = 2731 variables
LOH
Total = 1040 variables
DCT/CNT/PC
Total = 848 variables
IC
Total = 498 variables
Immune
Total = 1641 variables
c
DEG Obese+sGCstim vs. Obese
Endo
Total = 627 variables
Podo
Total = 1366 variables
Stroma
Total = 1169 variables
Prox tub
Total = 2561 variables
LOH
Total = 959 variables
DCT/CNT/PC
Total = 592 variables
IC
Total = 536 variables
Immune
Total = 1384 variables
Up
Down
−Log10 P
Log2 fold change

Figure S4. Differentially expressed genes. Related to Figure 2.

(a-c) Volcano plots of differentially expressed genes (DEGs) comparing Obese vs. Lean **(a)**, Obese+sGCact vs. Obese **(b)**, and Obese+sGCstim vs. Obese samples **(c)**, respectively. Comparisons are given separately for high-level cell types. X axes indicate log_2-fold change (Model-based Analysis of Single-cell Transcriptomics, MAST) and y axis indicates statistical significance-adjusted p = $-log_{10}$. Endo, endothelial cell; Podo, podocyte; Stroma, stromal cell; Prox tub, proximal tubule; LOH, loop of Henle; DCT/CNT/PC, distal convoluted tubule/connecting tubule/collecting duct principal cell; IC, collecting duct intercalated cell; Immune, immune cell.

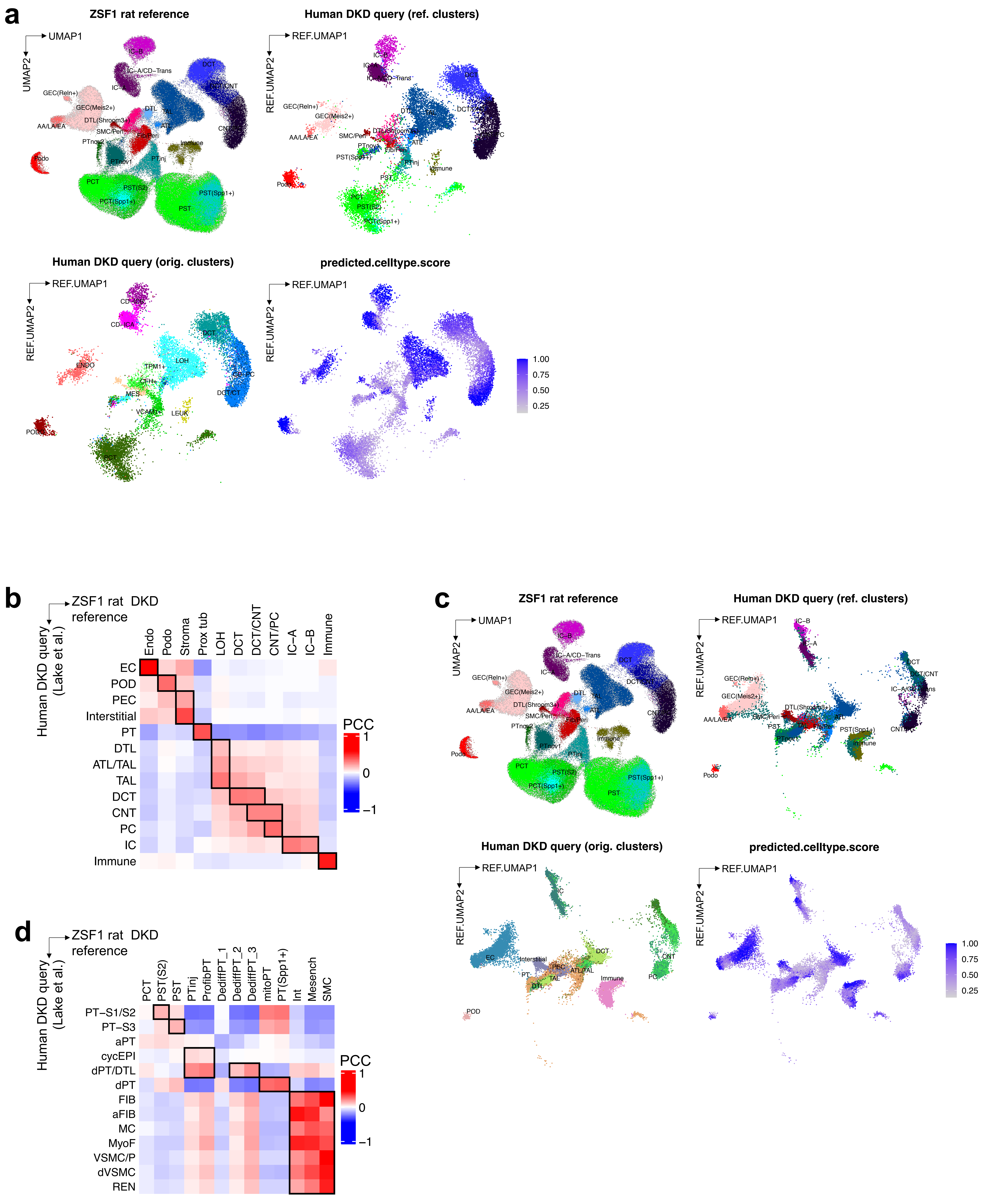

a
ZSF1 rat reference
UMAP1
UMAP2
Human DKD query (ref. clusters)
REF.UMAP1
REF.UMAP2
Human DKD query (orig. clusters)
predicted.celltype.score
1.00
0.75
0.50
0.25
IC–B
IC–A/CD–Trans
IC–A
DCT
DCT/CNT
CNT
GEC(Reln+)
GEC(Meis2+)
DTL
TAL
ATL
DTL(Shroom3+)
AA/LA/EA
SMC/Peri
Fib/Peri
PTnov2
PTnov1
Immune
PTinj
Podo
PCT
PST(S2)
PCT(Spp1+)
PST(Spp1+)
PST
CD–ICB
CD–ICA
DCT
ENDO
LOH
TPM1+
CFH+
MES
CD–PC
DCT/CT
VCAM1+
LEUK
POD
PCT
b
ZSF1 rat DKD reference
Human DKD query (Lake et al.)
Endo
Podo
Stroma
Prox tub
LOH
DCT
DCT/CNT
CNT/PC
IC–A
IC–B
Immune
EC
POD
PEC
Interstitial
PT
DTL
ATL/TAL
TAL
DCT
CNT
PC
IC
Immune
PCC
1
0
−1
c
ZSF1 rat reference
Human DKD query (ref. clusters)
Human DKD query (orig. clusters)
predicted.celltype.score
IC
EC
Interstitial
PEC
PT
DTL
TAL
ATL/TAL
DCT
CNT
PC
Immune
POD
d
ZSF1 rat DKD reference
Human DKD query (Lake et al.)
PCT
PST(S2)
PST
PTinj
ProfibPT
DediffPT_1
DediffPT_2
DediffPT_3
mitoPT
PT(Spp1+)
Int
Mesench
SMC
PT–S1/S2
PT–S3
aPT
cycEPI
dPT/DTL
dPT
FIB
aFIB
MC
MyoF
VSMC/P
dVSMC
REN
PCC
1
0
−1

Figure S5. ZSF1 rat integration with human DKD. Related to Figure 2.

(a) UMAP representations of ZSF1 rat reference with low-level clustering (top left) and human DKD query dataset (Wilson et al.) projected onto ZSF1 rat reference dataset (other subpanels). Human DKD subpanels are annotated/colored by ZSF1 rat reference clustering (top right), by original clustering (bottom left), and by predicted cell type score (bottom right).

(b) Pearson correlation coefficient (PCC) matrix of average cell type gene expression between ZSF1 rats (Lean and Obese samples only) and a corresponding human snRNA-seq dataset from the Kidney Precision Medicine Project (KPMP, Lake et al.) with Control and DKD kidney samples; ATL, ascending thin limb; CNT, connecting tubule; DCT, distal convoluted tubule; DTL, descending thin limb; Endo/EC, endothelial cell; IC, collecting duct intercalated cell; LEUK, leukocyte; LOH, loop of Henle; MES, mesangial cells; PC, collecting duct principal cell; PEC, parietal epithelial cell; Podo/POD, podocytes; Prox tub/PT, proximal tubule; TAL, thick ascending limb.

(c) Similar UMAP representations as in **(a)**, but with dataset from Kidney Precision Medicine Project (KPMP, Lake et al.).

(d) Similar correlation analysis as in **(b)**, but with PT and stromal cell subclustering. aFIB, "adaptive" fibroblast; aPT, "adaptive epithelial state" proximal tubule; cycEPI, cycling epithelial cells; dPT, "degenerative" proximal tubule; DTL, descending thin limb; dVSCM, "degenerative" vascular smooth muscle cell; FIB, fibroblast; Int, interstitial cell; MC, mesangial cell; Mesench, mesenchymal cell; mitoPT, high mitochondrial gene PT; MyoF, myofibroblast; PCT, proximal convoluted tubule; PST, proximal straight tubule; PT, proximal tubule; PTinj, injured PT; ProfibPT, profibrotic PT; DediffPT, dedifferentiated PT; PT(Spp1+), $Spp1^+$ PT; REN, Renin-positive juxtaglomerular granular cell; SMC, smooth muscle cell; VSCM/P, vascular smooth muscle cell/pericyte.

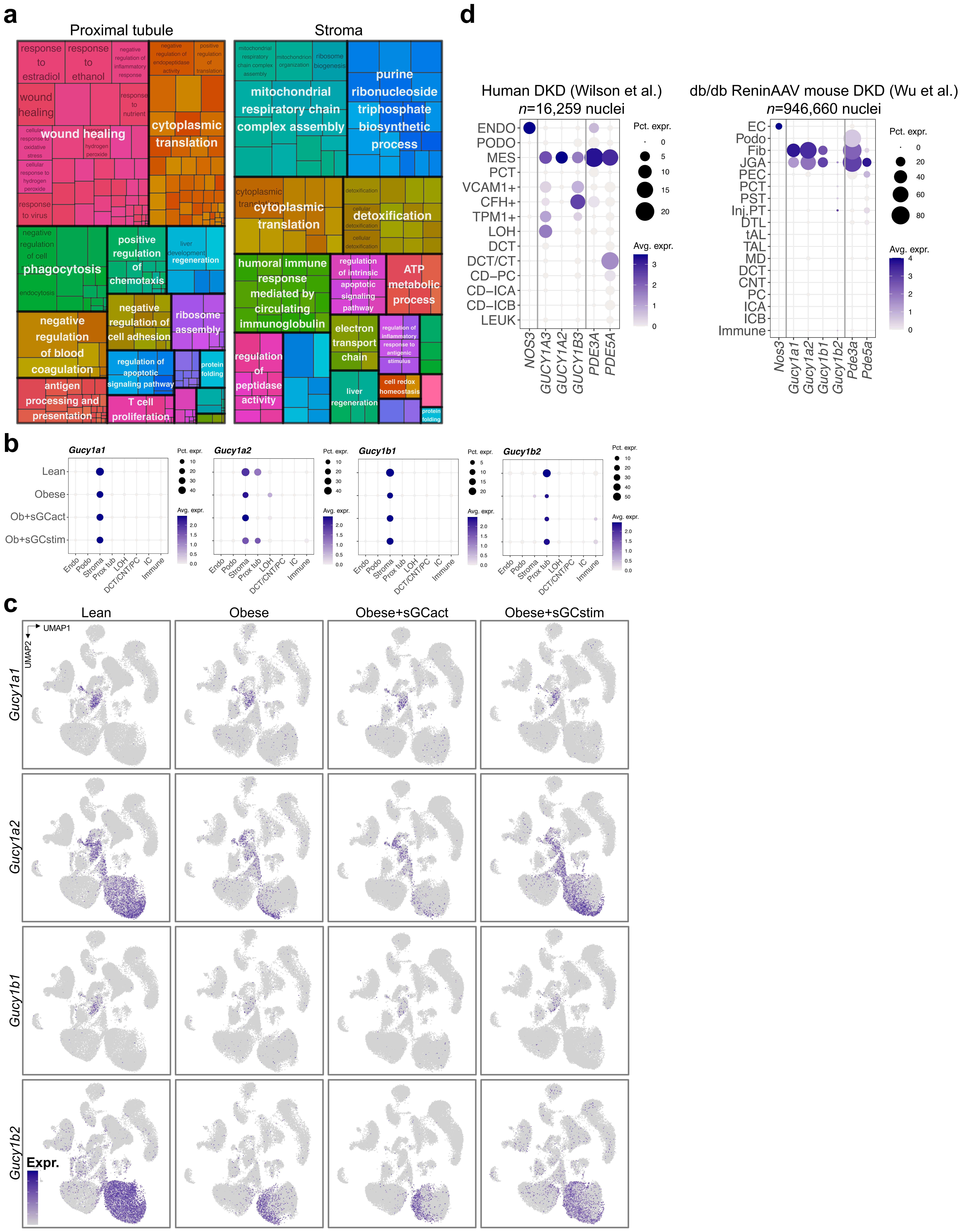
a
Proximal tubule
Stroma
response to estradiol
response to ethanol
wound healing
cytoplasmic translation
phagocytosis
positive regulation of chemotaxis
liver development regeneration
negative regulation of blood coagulation
negative regulation of cell adhesion
ribosome assembly
regulation of apoptotic signaling pathway
protein folding
antigen processing and presentation
T cell proliferation
mitochondrial respiratory chain complex assembly
purine ribonucleoside triphosphate biosynthetic process
detoxification
humoral immune response mediated by circulating immunoglobulin
regulation of intrinsic apoptotic signaling pathway
ATP metabolic process
electron transport chain
regulation of peptidase activity
liver regeneration
cell redox homeostasis
b
Gucy1a1
Gucy1a2
Gucy1b1
Gucy1b2
Lean
Obese
Ob+sGCact
Ob+sGCstim
Endo
Podo
Stroma
Prox tub
LOH
DCT/CNT/PC
IC
Immune
Pct. expr.
Avg. expr.
c
Lean
Obese
Obese+sGCact
Obese+sGCstim
UMAP1
UMAP2
Expr.
d
Human DKD (Wilson et al.)
n=16,259 nuclei
ENDO
PODO
MES
PCT
VCAM1+
CFH+
TPM1+
LOH
DCT
DCT/CT
CD–PC
CD–ICA
CD–ICB
LEUK
NOS3
GUCY1A3
GUCY1A2
GUCY1B3
PDE3A
PDE5A
db/db ReninAAV mouse DKD (Wu et al.)
n=946,660 nuclei
EC
Podo
Fib
JGA
PEC
PCT
PST
Inj.PT
DTL
tAL
TAL
MD
DCT
CNT
PC
ICA
ICB
Immune
Nos3
Gucy1a1
Gucy1a2
Gucy1b1
Gucy1b2
Pde3a
Pde5a
Pct. expr.
Avg. expr.

Figure S6. GO term analysis of tensor decomposition factor 1-loading genes and PT and stromal cell type specificity of sGC gene expression. Related to Figure 2.

(a) Gene ontology (GO) terms of factor 1-loading genes in proximal tubule and stromal cells.

(b) Expression dot plots for sGC genes, stratified by experimental group (rows) and high-level clustering (columns); dot size denotes percentage of cells expressing the marker. Color scale represents average gene expression values.

(c) Feature plots corresponding to **(b)**.

(d) Expression dot plots for NO/sGC/cGAMP pathway genes in human DKD (left) and murine DKD (right) datasets, corresponding to **Fig. 2g**; dot size denotes percentage of cells expressing the marker. Color scale represents average gene expression values.

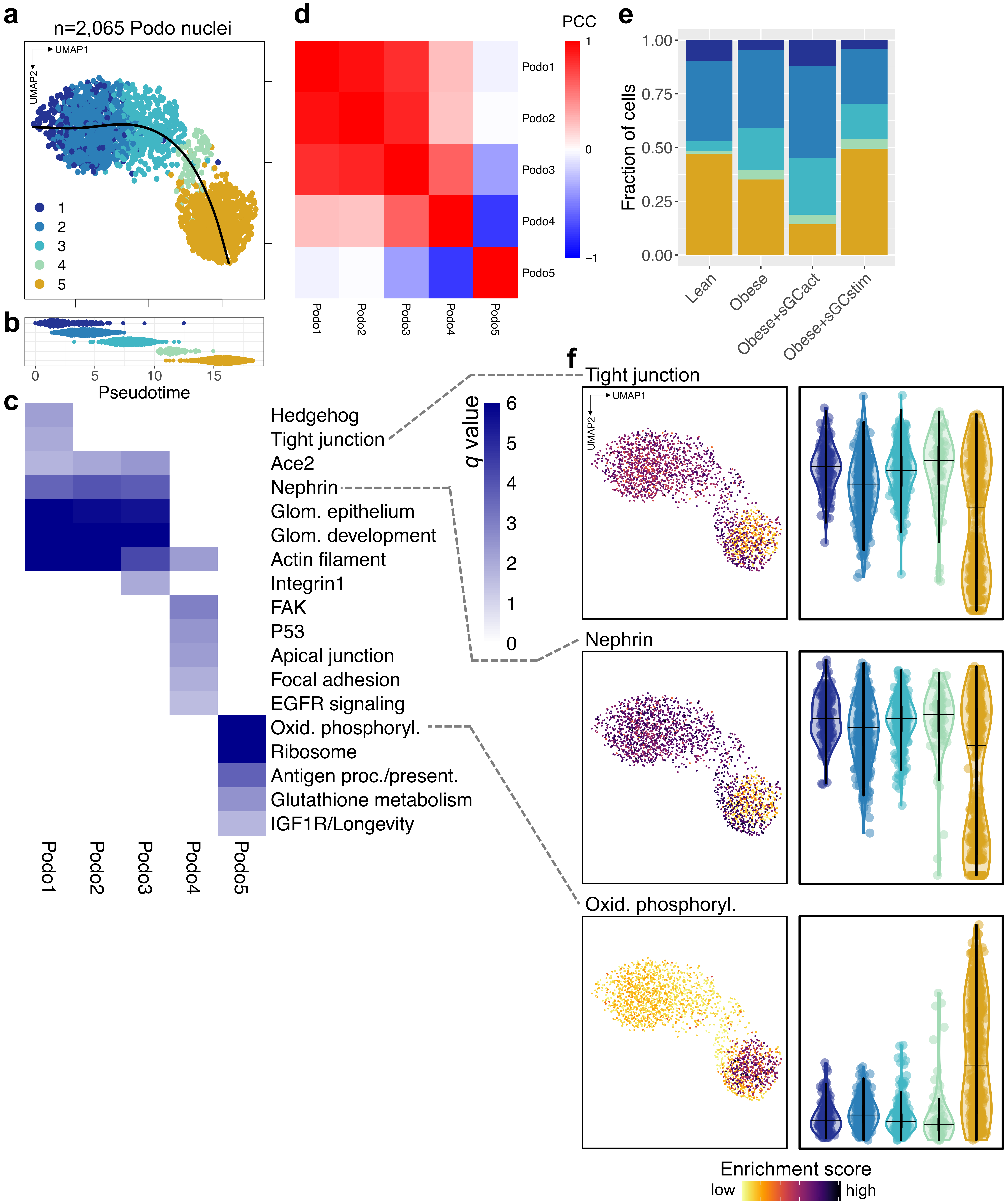
a
n=2,065 Podo nuclei
UMAP1
UMAP2
1
2
3
4
5
b
0
5
10
15
Pseudotime
c
Hedgehog
Tight junction
Ace2
Nephrin
Glom. epithelium
Glom. development
Actin filament
Integrin1
FAK
P53
Apical junction
Focal adhesion
EGFR signaling
Oxid. phosphoryl.
Ribosome
Antigen proc./present.
Glutathione metabolism
IGF1R/Longevity
Podo1
Podo2
Podo3
Podo4
Podo5
q value
6
5
4
3
2
1
0
d
PCC
1
0
−1
Podo1
Podo2
Podo3
Podo4
Podo5
e
Fraction of cells
1.00
0.75
0.50
0.25
0.00
Lean
Obese
Obese+sGCact
Obese+sGCstim
f
Tight junction
Nephrin
Oxid. phosphoryl.
Enrichment score
low
high

Figure S7. Podocyte subclustering. Related to Figure 2.

(a) Integrated UMAP of 2,065 high-quality podocyte nuclei from 12 rat kidney samples. Black line indicates Slingshot-inferred trajectory from clusters 1 through 5.

(b) Pseudotime values across podocyte clusters 1 through 5. Colors correspond to **(a)**.

(c) Heatmaps shows enrichment of top pseudotime-specific GO biological processes and KEGG pathways.

(d) Pearson correlation coefficient (PCC) matrix of average cell type gene expression between podocyte clusters 1 through 5.

(e) Cell fractions of podocyte clusters stratified by experimental group. Colors correspond to **(a)**.

(f) Scoring of gene sets corresponding to representative pathways from **(c)**. Left panels show pathway enrichment along the podocyte trajectory. Right panels show gene set scores by podocyte cluster. Colors correspond to **(a)**.

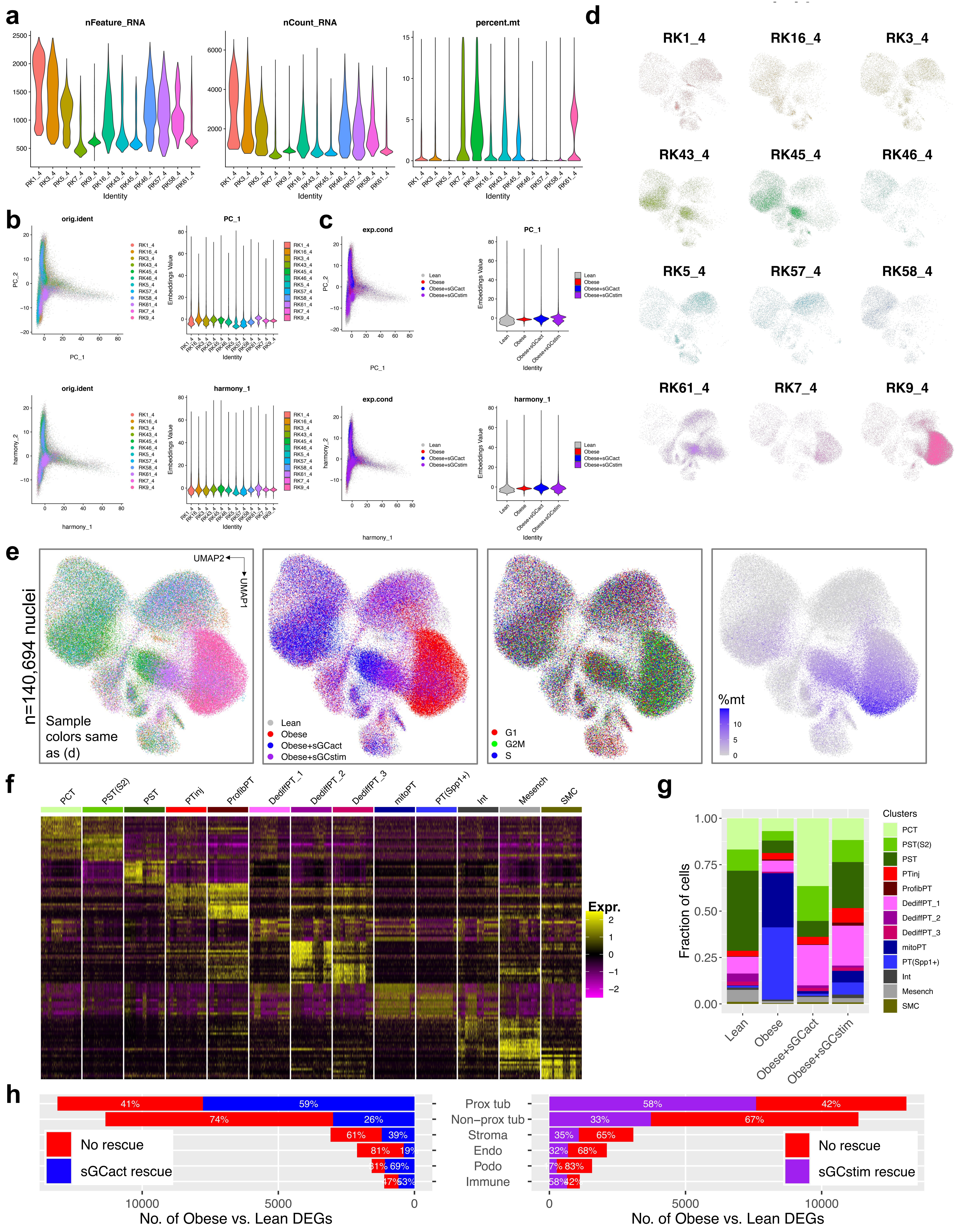
a
nFeature_RNA
nCount_RNA
percent.mt
Identity
b
orig.ident
PC_1
PC_2
Embeddings Value
harmony_1
harmony_2
c
exp.cond
Lean
Obese
Obese+sGCact
Obese+sGCstim
d
RK1_4
RK16_4
RK3_4
RK43_4
RK45_4
RK46_4
RK5_4
RK57_4
RK58_4
RK61_4
RK7_4
RK9_4
e
UMAP2
UMAP1
n=140,694 nuclei
Sample colors same as (d)
G1
G2M
S
%mt
f
PCT
PST(S2)
PST
PTinj
ProfibPT
DediffPT_1
DediffPT_2
DediffPT_3
mitoPT
PT(Spp1+)
Int
Mesench
SMC
Expr.
g
Fraction of cells
Clusters
h
Prox tub
Non−prox tub
Stroma
Endo
Podo
Immune
No rescue
sGCact rescue
sGCstim rescue
No. of Obese vs. Lean DEGs

Figure S8. PT and stromal cell subclustering. Related to Figure 3.

(a) Violin plots showing number of informative genes (nFeature_RNA), unique molecular identifiers (nCount_RNA), and percentage of mitochondrial genes (percent.mt) per single cell.

(b) Principal component (PC) representation (left) and violin plots of corresponding embeddings values (right) before (top) and after batch correction (bottom) using Harmony, stratified by individual sample.

(c) Same as in **(b)**, stratified by experimental group.

(d) Integrated UMAP corresponding to **Fig. 3a**, stratified by sample.

(e) Integrated UMAP corresponding to **Fig. 3a**, annotated by sample, experimental group, cell cycle phase, and mitochondrial percentage (%mt), respectively.

(f) Heatmap of top 10 differentially expressed genes for subclusters. PCT, proximal convoluted tubule; PST(S2), proximal straight tubule (segment 2); PST, proximal straight tubule; PTinj, injured PT; ProfibPT, profibrotic PT; DediffPT, dedifferentiated PT; mitoPT, high mitochondrial gene PT; PT(Spp1+), $Spp1^{+}$ PT; Int, interstitial cell; Mesench, mesenchymal cell; SMC, smooth muscle cell.

(g) Cell fractions of PT and stromal cell subclusters stratified by experimental group. Colors correspond to **(f)**.

(h) Bar graphs representing the number of genes differentially expressed (DEG) between Obese and Lean samples in coarse-grained, high-level cell types. Percentages indicate absent or present rescue effect (normalization) for DEG comparison between sGC modulator-treated rats (sGCstim, sGCact) and vehicle-treated rats.

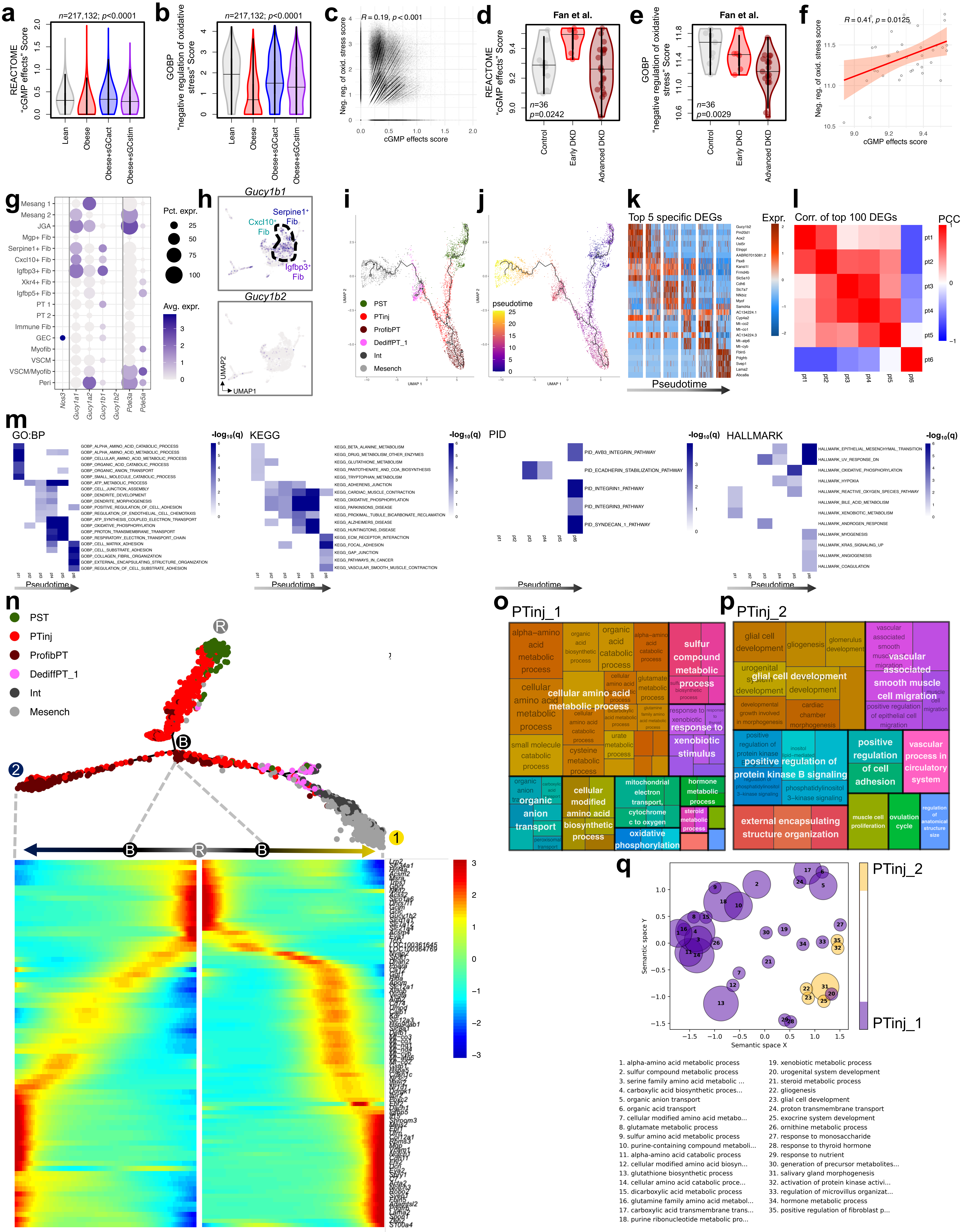

a
n=217,132; p<0.0001
REACTOME "cGMP effects" Score
Lean
Obese
Obese+sGCact
Obese+sGCstim
b
n=217,132; p<0.0001
GOBP "negative regulation of oxidative stress" Score
c
R = 0.19, p < 0.001
Neg. reg. of oxid. stress score
cGMP effects score
d
Fan et al.
REACTOME "cGMP effects" Score
n=36
p=0.0242
Control
Early DKD
Advanced DKD
e
Fan et al.
GOBP "negative regulation of oxidative stress" Score
n=36
p=0.0029
f
R = 0.41, p = 0.0125
Neg. reg. of oxid. stress score
cGMP effects score
g
Mesang 1
Mesang 2
JGA
Mgp+ Fib
Serpine1+ Fib
Cxcl10+ Fib
Igfbp3+ Fib
Xkr4+ Fib
Igfbp5+ Fib
PT 1
PT 2
Immune Fib
GEC
Myofib
VSCM
VSCM/Myofib
Peri
Nos3
Gucy1a1
Gucy1a2
Gucy1b1
Gucy1b2
Pde3a
Pde5a
Pct. expr.
Avg. expr.
h
Gucy1b1
Serpine1+ Fib
Cxcl10+ Fib
Igfbp3+ Fib
Gucy1b2
UMAP2
UMAP1
i
PST
PTinj
ProfibPT
DediffPT_1
Int
Mesench
j
pseudotime
k
Top 5 specific DEGs
Expr.
Pseudotime
l
Corr. of top 100 DEGs
PCC
pt1
pt2
pt3
pt4
pt5
pt6
m
GO:BP
KEGG
PID
HALLMARK
-log10(q)
Pseudotime
n
PST
PTinj
ProfibPT
DediffPT_1
Int
Mesench
o
PTinj_1
p
PTinj_2
q
PTinj_2
PTinj_1
Semantic space X
Semantic space Y
1. alpha-amino acid metabolic process
2. sulfur compound metabolic process
3. serine family amino acid metabolic ...
4. carboxylic acid biosynthetic proces...
5. organic anion transport
6. organic acid transport
7. cellular modified amino acid metabo...
8. glutamate metabolic process
9. sulfur amino acid metabolic process
10. purine-containing compound metaboli...
11. alpha-amino acid catabolic process
12. cellular modified amino acid biosyn...
13. glutathione biosynthetic process
14. cellular amino acid catabolic proce...
15. dicarboxylic acid metabolic process
16. glutamine family amino acid metabol...
17. carboxylic acid transmembrane trans...
18. purine ribonucleotide metabolic pro...
19. xenobiotic metabolic process
20. urogenital system development
21. steroid metabolic process
22. gliogenesis
23. glial cell development
24. proton transmembrane transport
25. exocrine system development
26. ornithine metabolic process
27. response to monosaccharide
28. response to thyroid hormone
29. response to nutrient
30. generation of precursor metabolites...
31. salivary gland morphogenesis
32. activation of protein kinase activi...
33. regulation of microvillus organizat...
34. hormone metabolic process
35. positive regulation of fibroblast p...

Figure S9. Trajectory analysis highlights dynamic changes of PT cells towards profibrotic and mesenchymal cell states. Related to Figures 3 and 4.

(a-b) REACTOME "cGMP effects" **(a)** and GOBP "negative regulation of oxidative stress" scores **(b)** in the integrated ZSF1 rat DKD dataset by experimental groups; p values are given for one-way ANOVA (Tukey-corrected).

(c) Corresponding scatterplot for correlation of GOBP "negative regulation of oxidative stress" score (y axis) and REACTOME "cGMP effects" score (x axis); p value is given for linear regression.

(d-e) REACTOME "cGMP effects" **(d)** and GOBP "negative regulation of oxidative stress" scores **(e)** in a human kidney bulk RNA-seq dataset by DKD severity; p values are given for one-way ANOVA (Tukey-corrected).

(f) Corresponding scatterplot for correlation of GOBP "negative regulation of oxidative stress" score (y axis) and REACTOME "cGMP effects" score (x axis); p value is given for linear regression; error bands represent 95% confidence interval.

(g) Expression dot plot for NO/sGC/cGAMP pathway genes in stroma subclusters (rows), corresponding to **Fig. 3e**. Dot size denotes percentage of cells expressing the marker. Color scale represents average gene expression values. Mesang, mesangial cell; JGA, juxtaglomerular apparatus cell; Fib, fibroblast; PT, proximal tubule; GEC, glomerular endothelial cell; Myofib, myofibroblast; VSCM, vascular smooth muscle cell; Peri, pericyte.

(h) Feature plots for *Gucy1b1* and *Gucy1b2* in UMAP space.

(i-j) Monocle3-derived PT-Mesench trajectory, colored by cell clusters along the trajectory **(i)** and by pseudotime **(j)**, respectively. PST, proximal straight tubule; PTinj, injured PT; ProfibPT, profibrotic PT; DediffPT, dedifferentiated PT; Int, interstitial cell; Mesench, mesenchymal cell.

(k) Expression heatmap of top 5 differentially expressed genes (DEGs) along Monocle3-derived pseudotime. Rows represent DEGs, columns represent individual PT cells in bins along pseudotime.

(l) Pearson correlation coefficient (PCC) matrix of top 100 DEG expression between pseudotime bins along Monocle3-derived trajectory.

(m) Corresponding enrichment of top pseudotime-specific GO biological processes and KEGG, Pathway Interaction Database (PID), and HALLMARK pathways, respectively.

(n) Monocle2-derived PT-Mesench trajectory (top) and corresponding heatmap visualizing pseudotiome-dependent gene expression along lineages 1 and 2, respectively. R denotes root node, B denotes branching point, 1 and 2 denote end points of lineages 1 and 2, respectively.

(o-p) Gene ontology (GO) terms derived from top PTinj_1 **(o)** and PTinj_2-specific **(p)** DEGs, corresponding to **Fig. 3e**.

(q) GO terms enriched in PTinj_1 and PTinj_2, respectively, in latent semantic space.

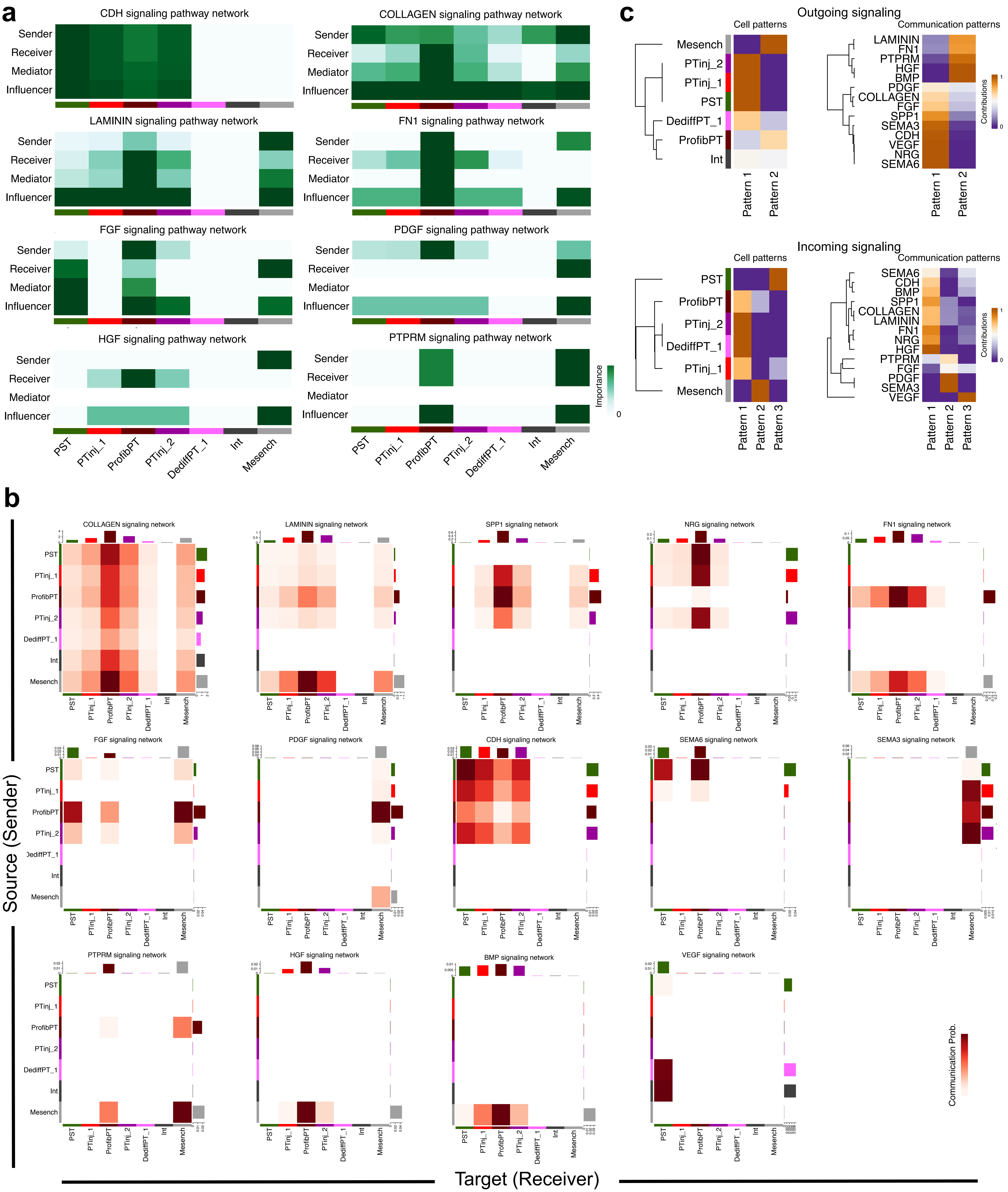
a
CDH signaling pathway network
COLLAGEN signaling pathway network
LAMININ signaling pathway network
FN1 signaling pathway network
FGF signaling pathway network
PDGF signaling pathway network
HGF signaling pathway network
PTPRM signaling pathway network
Sender
Receiver
Mediator
Influencer
Importance
0
PST
PTinj_1
ProfibPT
PTinj_2
DedifPT_1
Int
Mesench
c
Outgoing signaling
Cell patterns
Communication patterns
Mesench
PTinj_2
PTinj_1
PST
DedifPT_1
ProfibPT
Int
LAMININ
FN1
PTPRM
HGF
BMP
PDGF
COLLAGEN
FGF
SPP1
SEMA3
CDH
VEGF
NRG
SEMA6
Contributions
Pattern 1
Pattern 2
Incoming signaling
Pattern 3
b
COLLAGEN signaling network
LAMININ signaling network
SPP1 signaling network
NRG signaling network
FN1 signaling network
FGF signaling network
PDGF signaling network
CDH signaling network
SEMA6 signaling network
SEMA3 signaling network
PTPRM signaling network
HGF signaling network
BMP signaling network
VEGF signaling network
Source (Sender)
Target (Receiver)
Communication Prob.

Figure S10. Cell-cell communication analysis identifies a secretory phenotype of profibrotic PT. Related to Figure 5.

(a) Importance of signaling (sender, receiver, mediator, influencer) roles, as computed by CellChat centrality scores, stratified by cell clusters along the PT-Mesench trajectory, corresponding to **Fig. 5**. PST, proximal straight tubule; PTinj, injured PT; ProfibPT, profibrotic PT; DediffPT, dedifferentiated PT; Int, interstitial cell; Mesench, mesenchymal cell.

(b) Communication probability between source/sender (rows) and target/receiver cell clusters (columns) along the PT-Mesench trajectory, displayed individually for various signaling pathways.

(c) Communication patterns for outgoing (top) and incoming (bottom) signaling. Degree of contribution is plotted by cell clusters along the trajectory (left) and by signaling pathways (right), respectively.

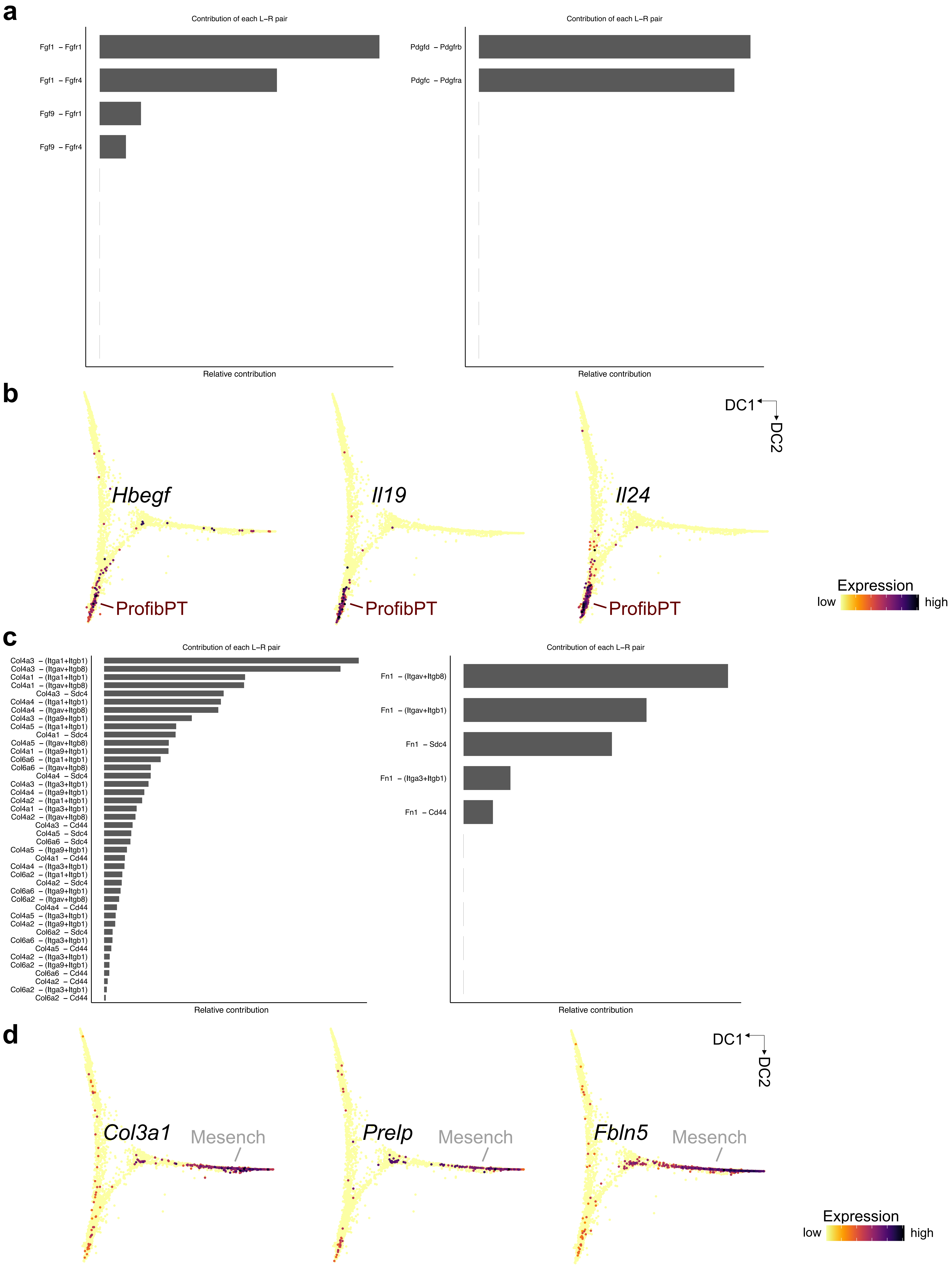
a
Contribution of each L–R pair
Fgf1 – Fgfr1
Fgf1 – Fgfr4
Fgf9 – Fgfr1
Fgf9 – Fgfr4
Relative contribution
Contribution of each L–R pair
Pdgfd – Pdgfrb
Pdgfc – Pdgfra
Relative contribution
b
Hbegf
ProfibPT
Il19
ProfibPT
Il24
ProfibPT
DC1
DC2
Expression
low
high
c
Contribution of each L–R pair
Col4a3 – (Itga1+Itgb1)
Col4a3 – (Itgav+Itgb8)
Col4a1 – (Itga1+Itgb1)
Col4a1 – (Itgav+Itgb8)
Col4a3 – Sdc4
Col4a4 – (Itga1+Itgb1)
Col4a4 – (Itgav+Itgb8)
Col4a3 – (Itga9+Itgb1)
Col4a5 – (Itga1+Itgb1)
Col4a1 – Sdc4
Col4a5 – (Itgav+Itgb8)
Col4a1 – (Itga9+Itgb1)
Col6a6 – (Itga1+Itgb1)
Col6a6 – (Itgav+Itgb8)
Col4a4 – Sdc4
Col4a3 – (Itga3+Itgb1)
Col4a4 – (Itga9+Itgb1)
Col4a2 – (Itga1+Itgb1)
Col4a1 – (Itga3+Itgb1)
Col4a2 – (Itgav+Itgb8)
Col4a3 – Cd44
Col4a5 – Sdc4
Col6a6 – Sdc4
Col4a5 – (Itga9+Itgb1)
Col4a1 – Cd44
Col4a4 – (Itga3+Itgb1)
Col6a2 – (Itga1+Itgb1)
Col4a2 – Sdc4
Col6a6 – (Itga9+Itgb1)
Col6a2 – (Itgav+Itgb8)
Col4a4 – Cd44
Col4a5 – (Itga3+Itgb1)
Col4a2 – (Itga9+Itgb1)
Col6a2 – Sdc4
Col6a6 – (Itga3+Itgb1)
Col4a5 – Cd44
Col4a2 – (Itga3+Itgb1)
Col6a2 – (Itga9+Itgb1)
Col6a6 – Cd44
Col4a2 – Cd44
Col6a2 – (Itga3+Itgb1)
Col6a2 – Cd44
Relative contribution
Contribution of each L–R pair
Fn1 – (Itgav+Itgb8)
Fn1 – (Itgav+Itgb1)
Fn1 – Sdc4
Fn1 – (Itga3+Itgb1)
Fn1 – Cd44
Relative contribution
d
Col3a1
Mesench
Prelp
Mesench
Fbln5
Mesench
DC1
DC2
Expression
low
high

Figure S11. Secretory and ECM-receptor phenotypes of profibrotic PT and mesenchymal cells, respectively. Related to Figure 5.

(a, c) Relative contribution of secreted extracellular matrix (ECM) factors **(a)** and ECM-receptor signaling **(b)**.

(b, d) Feature plots for representative secreted ECM factor genes *(Hbegf, Il19, Il24)* **(b)** and representative core matrisome genes *(Col3a1, Prelp, Fbln5)* **(d)** along the trajectory in diffusion map space, corresponding to **Figs. 5g, j**. ProfibPT, profibrotic PT; Mesench, mesenchymal cell.

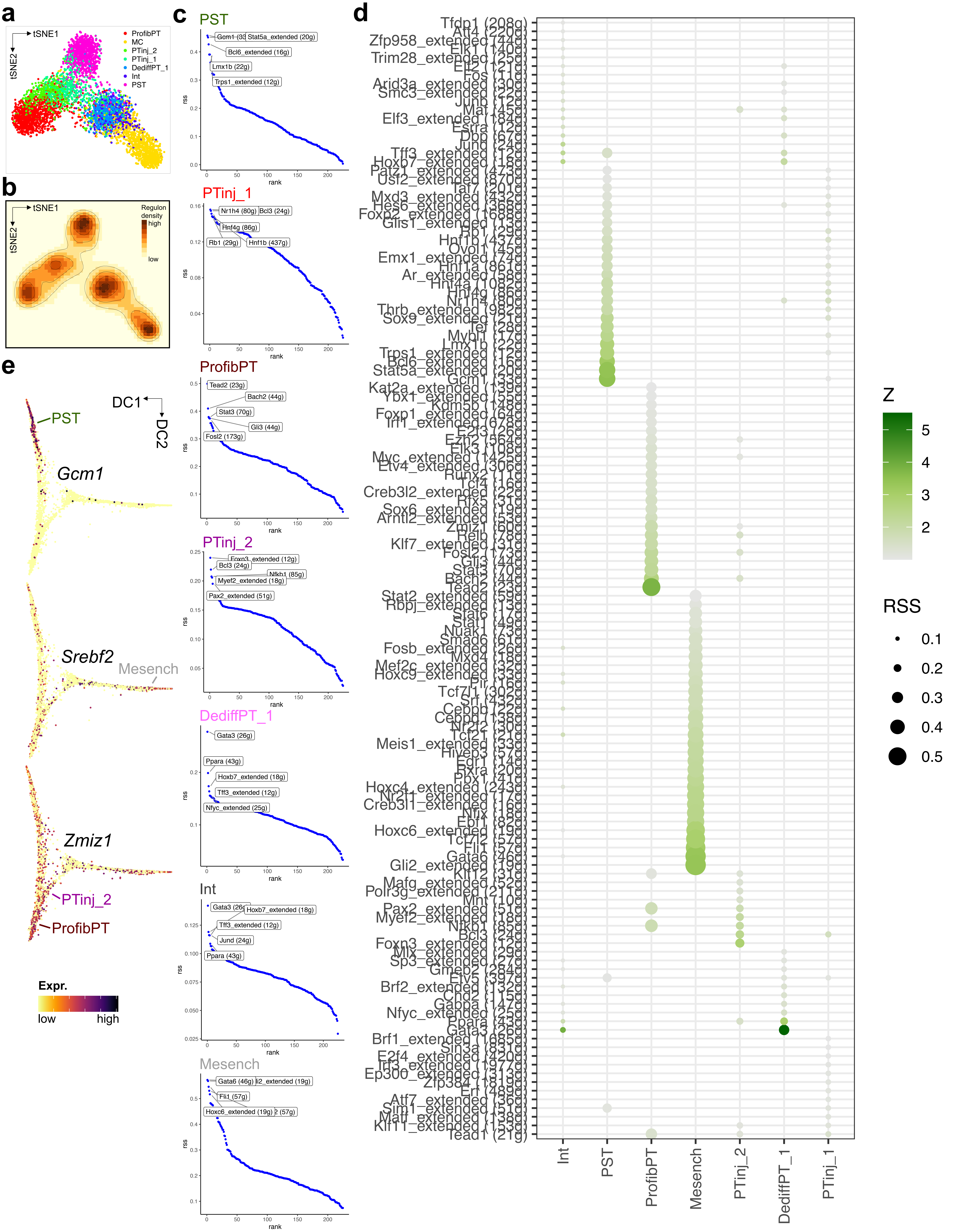
a
tSNE1
tSNE2
ProfibPT
MC
PTinj_2
PTinj_1
DediffPT_1
Int
PST
b
tSNE1
tSNE2
Regulon density
high
low
c
PST
Stat5a_extended (20g)
Bcl6_extended (16g)
Lmx1b (22g)
Trps1_extended (12g)
rss
rank
PTinj_1
Nr1h4 (80g)
Bcl3 (24g)
Hnf4g (86g)
Rb1 (29g)
Hnf1b (437g)
ProfibPT
Tead2 (23g)
Bach2 (44g)
Stat3 (70g)
Gli3 (44g)
Fosl2 (173g)
PTinj_2
Foxn3_extended (12g)
Bcl3 (24g)
Nfkb1 (85g)
Myef2_extended (18g)
Pax2_extended (51g)
DediffPT_1
Gata3 (26g)
Ppara (43g)
Hoxb7_extended (18g)
Tff3_extended (12g)
Nfyc_extended (25g)
Int
Hoxb7_extended (18g)
Tff3_extended (12g)
Jund (24g)
Ppara (43g)
Mesench
Gata6 (46g)
Fli1 (57g)
Hoxc6_extended (19g)
d
Z
RSS
0.1
0.2
0.3
0.4
0.5
Int
PST
ProfibPT
Mesench
PTinj_2
DediffPT_1
PTinj_1
e
DC1
DC2
PST
Gcm1
Srebf2
Mesench
Zmiz1
PTinj_2
ProfibPT
Expr.
low
high

Figure S12. Gene regulatory network analysis highlights cell type-specific transcription factors driving the PT-to-mesenchymal trajectory and prioritizes cell types of action for sGC modulation. Related to Figure 6.

(a) t-distributed stochastic neighbor embedding (t-SNE) reduction plot of regulon-derived clustering of cell types along the PT-Mesench trajectory. PST, proximal straight tubule; PTinj, injured PT; ProfibPT, profibrotic PT; DediffPT, dedifferentiated PT; Int, interstitial cell; Mesench, mesenchymal cell.

(b) tSNE representation of regulon density as a surrogate for stability of regulon states.

(c) Regulons ranked according to their specificity scores (rss) for individual cell types along the PT-Mesench trajectory. The top 5 specific regulons per cell type are annotated.

(d) Dot plot of regulons (rows) according to cell types along the PT-Mesench trajectory (columns). Dot size denotes regulon specificity score (RSS), color denotes z-scored RSS.

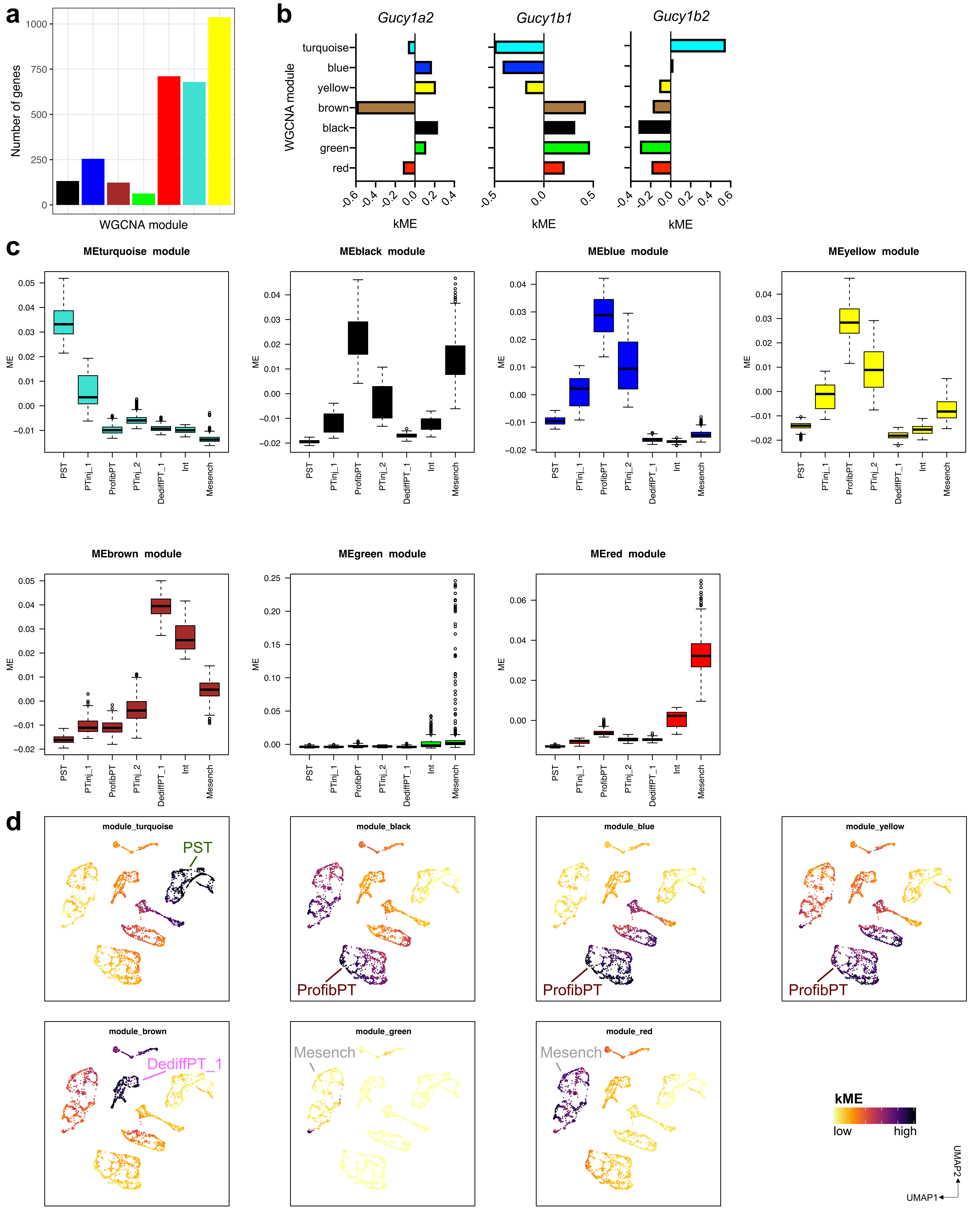
a
Number of genes
WGCNA module
b
Gucy1a2
Gucy1b1
Gucy1b2
WGCNA module
turquoise
blue
yellow
brown
black
green
red
kME
c
MEturquoise module
MEblack module
MEblue module
MEyellow module
MEbrown module
MEgreen module
MEred module
ME
PST
PTinj_1
ProfibPT
PTinj_2
DediffPT_1
Int
Mesench
d
module_turquoise
module_black
module_blue
module_yellow
module_brown
module_green
module_red
PST
ProfibPT
DediffPT_1
Mesench
kME
low
high
UMAP1
UMAP2

Figure S13. WGCNA analysis in PT-Mesench trajectory metanuclei. Related to Figure 7.

(a) Bar graph depicting the number of genes in WGCNA-derived modules.

(b) Intramodular connectivity (kME) values of *Gucy1a2, Gucy1b1*, and *Gucy1b2*.

(c) Module eigengene (ME) scores of WGCNA modules displayed as Tukey box plots (outliers denoted as dots outside box plot whiskers) by cell clusters along the PT-Mesench trajectory (n=3,120 metanuclei). PST, proximal straight tubule; PTinj, injured PT; ProfibPT, profibrotic PT; DediffPT, dedifferentiated PT; Int, interstitial cell; Mesench, mesenchymal cell.

(d) kME values of individual WGCNA modules visualized in UMAP space.

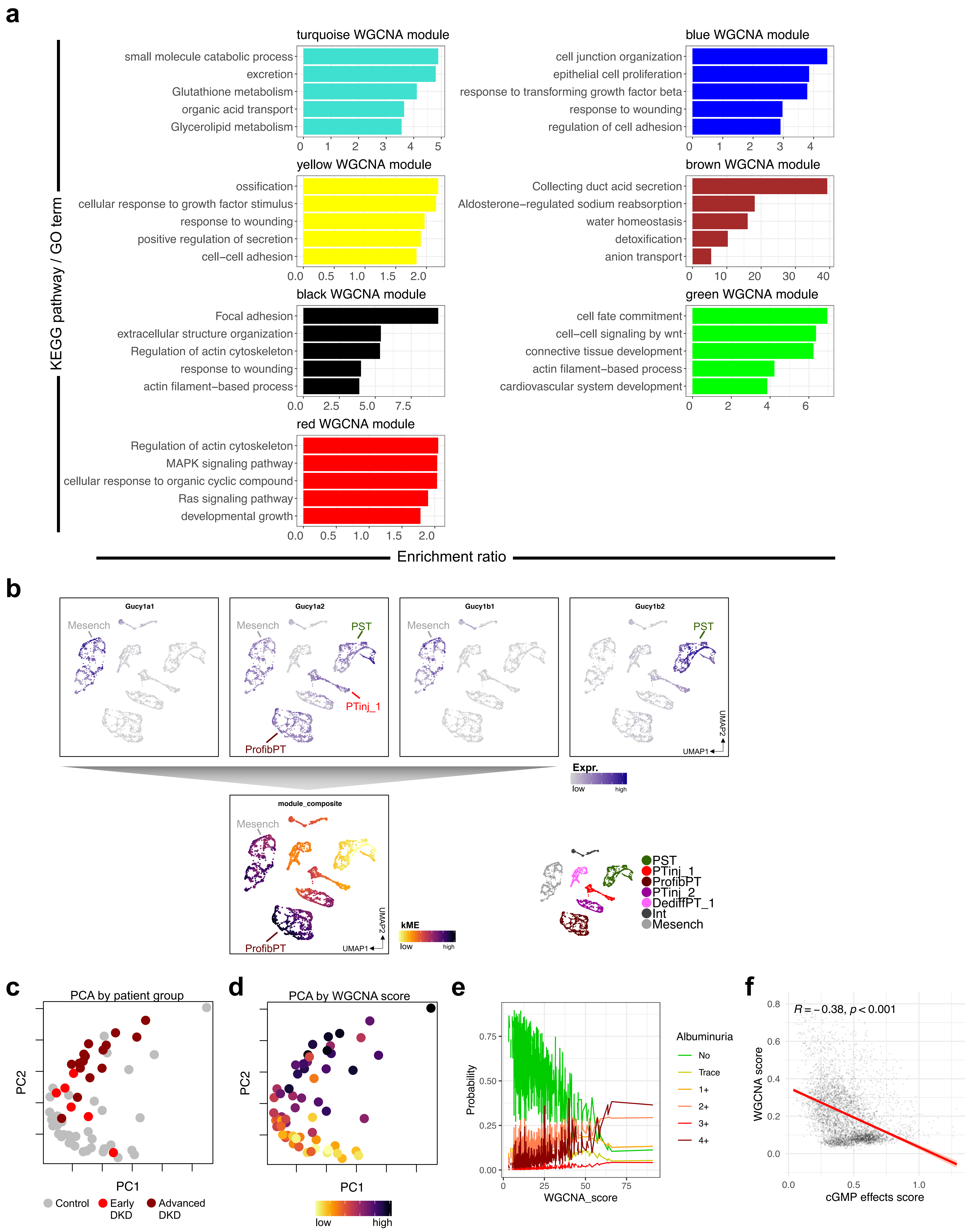
a
KEGG pathway / GO term
turquoise WGCNA module
small molecule catabolic process
excretion
Glutathione metabolism
organic acid transport
Glycerolipid metabolism
blue WGCNA module
cell junction organization
epithelial cell proliferation
response to transforming growth factor beta
response to wounding
regulation of cell adhesion
yellow WGCNA module
ossification
cellular response to growth factor stimulus
response to wounding
positive regulation of secretion
cell–cell adhesion
brown WGCNA module
Collecting duct acid secretion
Aldosterone–regulated sodium reabsorption
water homeostasis
detoxification
anion transport
black WGCNA module
Focal adhesion
extracellular structure organization
Regulation of actin cytoskeleton
response to wounding
actin filament–based process
green WGCNA module
cell fate commitment
cell–cell signaling by wnt
connective tissue development
actin filament–based process
cardiovascular system development
red WGCNA module
Regulation of actin cytoskeleton
MAPK signaling pathway
cellular response to organic cyclic compound
Ras signaling pathway
developmental growth
Enrichment ratio
b
Gucy1a1
Gucy1a2
Gucy1b1
Gucy1b2
Mesench
PST
PTinj_1
ProfibPT
UMAP1
UMAP2
Expr.
low
high
module_composite
kME
PTinj_2
DediffPT_1
Int
c
PCA by patient group
PC1
PC2
Control
Early DKD
Advanced DKD
d
PCA by WGCNA score
e
Probability
WGCNA_score
Albuminuria
No
Trace
1+
2+
3+
4+
f
R = –0.38, p < 0.001
WGCNA score
cGMP effects score

Figure S14. WGCNA-derived sGC co-expression modules correlate with human DKD outcome variables. Related to Figure 7.

(a) Enrichment ratios of top enriched KEGG pathway and GO terms, derived by overrepresentation analysis of WGCNA module genes. The graph is split into subpanels by WGCNA module.

(b) Expression of sGC genes *(Gucy1a1, Gucy1a2, Gucy1b1, Gucy1b2)* (top) and kME values of composite WGCNA score (bottom) visualized in UMAP space. Insert (bottom right) indicates cell types along the PT-Mesench trajectory. PST, proximal straight tubule; PTinj, injured PT; ProfibPT, profibrotic PT; DediffPT, dedifferentiated PT; Int, interstitial cell; Mesench, mesenchymal cell.

(c-d) Principal component analysis (PCA) plots stratifying microdissected kidney tubule RNA-seq samples from human individuals with and without DKD by disease severity **(c)** and by WGCNA score **(d)**.

(e) Ordinal logistic regression showed that as WGCNA score increased, probability of human subjects to have any form of albuminuria increased and probability to be normoalbuminuric decreased.

(f) Scatterplot for correlation of WGCNA score (y axis) and REACTOME "cGMP effects" score (x axis); p value is given for linear regression, error bands represent 95% confidence interval.
