# Supplementary material for: Treatment effects of soluble guanylate cyclase modulation on diabetic kidney disease at single-cell resolution
Source: Cell Rep Med. 2023 Apr 5;4(4):100992. doi: 10.1016/j.xcrm.2023.100992 (PMC10140477; doi:10.1016/j.xcrm.2023.100992)
Supplement: Document S2. Article plus supplemental information [file mmc24.pdf]

Cell Reports Medicine

Article

Treatment effects of soluble guanylate cyclase modulation on diabetic kidney disease at single-cell resolution

Graphical abstract

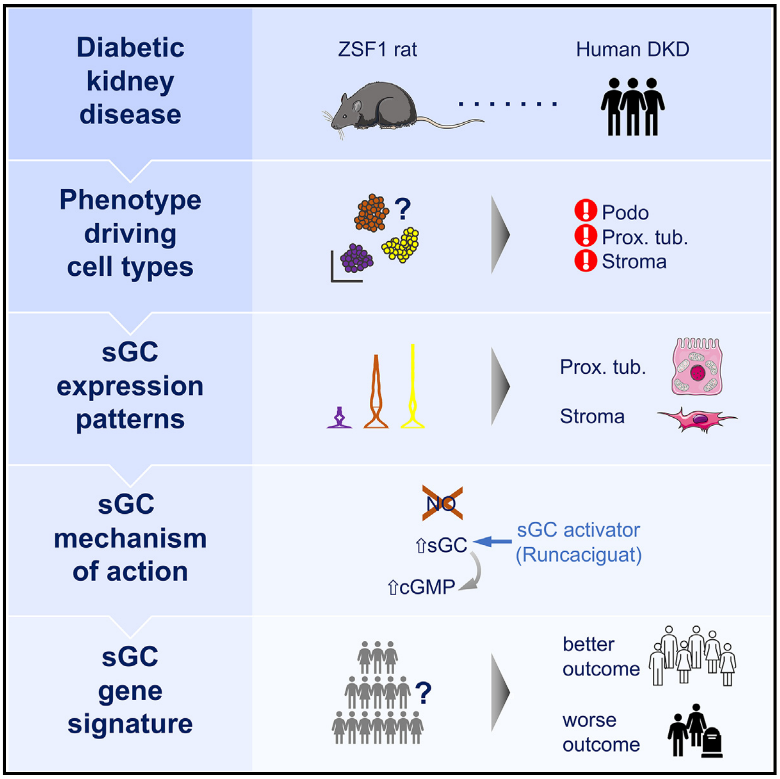

Authors

Michael S. Balzer, Mira Pavkovic, Julia Frederick, ..., Peter Sandner, Manuel Grundmann, Katalin Susztak

Correspondence

ksusztak@pennmedicine.upenn.edu

In brief

Balzer et al. show how ZSF1 rats recapitulate human diabetic kidney disease (DKD), the most common cause of renal failure, on a phenotypic and single-cell transcriptomic level. Their study prioritizes disease-relevant cell types and demonstrates the benefits of pharmacological modulation of soluble guanylate cyclase, a promising DKD drug target.

Highlights

- ZSF1 rats recapitulate human DKD on phenotypic and single-cell transcriptomic levels
- Proximal tubule and stroma cells exhibit a continuous lineage relationship
- Pharmacological sGC activation confers benefits over sGC stimulation
- SGC co-expression modules stratify human kidneys by DKD-relevant disease measures

Balzer et al., 2023, Cell Reports Medicine *4*, 100992
April 18, 2023 © 2023 The Author(s).
https://doi.org/10.1016/j.xcrm.2023.100992

CellPress

Cell Reports Medicine

CellPress
OPEN ACCESS

Article

Treatment effects of soluble guanylate cyclase modulation on diabetic kidney disease at single-cell resolution

Michael S. Balzer,[1,2,3,4] Mira Pavkovic,[5] Julia Frederick,[1,2] Amin Abedini,[1,2] Alexius Freyberger,[5] Julia Vienenkötter,[5] Ilka Mathar,[5] Krystyna Siudak,[5] Frank Eitner,[5,6] Peter Sandner,[5,7] Manuel Grundmann,[5] and Katalin Susztak[1,2,8,9,*]

[1]Renal, Electrolyte, and Hypertension Division, Department of Medicine, Perelman School of Medicine, University of Pennsylvania, Philadelphia, PA 19104, USA
[2]Institute for Diabetes, Obesity and Metabolism, Perelman School of Medicine, University of Pennsylvania, Philadelphia, PA 19104, USA
[3]Department of Nephrology and Medical Intensive Care, Charité – Universitätsmedizin Berlin, 10117 Berlin, Germany
[4]Berlin Institute of Health at Charité – Universitätsmedizin Berlin, BIH Biomedical Innovation Academy, BIH Charité Clinician Scientist Program, 10117 Berlin, Germany
[5]Bayer AG, Research and Early Development, Pharma Research Center, 42096 Wuppertal, Germany
[6]Division of Nephrology and Clinical Immunology, RWTH Aachen University, 52062 Aachen, Germany
[7]Department of Pharmacology, Hannover Medical School, 30625 Hannover, Germany
[8]Department of Genetics, Perelman School of Medicine, University of Pennsylvania, Philadelphia, PA 19104, USA
[9]Lead contact
*Correspondence: ksusztak@pennmedicine.upenn.edu
https://doi.org/10.1016/j.xcrm.2023.100992

SUMMARY

Diabetic kidney disease (DKD) is the most common cause of renal failure. Therapeutics development is hampered by our incomplete understanding of animal models on a cellular level. We show that ZSF1 rats recapitulate human DKD on a phenotypic and transcriptomic level. Tensor decomposition prioritizes proximal tubule (PT) and stroma as phenotype-relevant cell types exhibiting a continuous lineage relationship. As DKD features endothelial dysfunction, oxidative stress, and nitric oxide depletion, soluble guanylate cyclase (sGC) is a promising DKD drug target. sGC expression is specifically enriched in PT and stroma. In ZSF1 rats, pharmacological sGC activation confers considerable benefits over stimulation and is mechanistically related to improved oxidative stress regulation, resulting in enhanced downstream cGMP effects. Finally, we define sGC gene co-expression modules, which allow stratification of human kidney samples by DKD prevalence and disease-relevant measures such as kidney function, proteinuria, and fibrosis, underscoring the relevance of the sGC pathway to patients.

INTRODUCTION

Chronic kidney disease (CKD) is the fourth fastest growing cause of death, affecting >850 million people worldwide.[1] Patients with CKD have 3- to 5-fold increased mortality.[2] The survival rate for kidney failure (end-stage renal disease [ESRD]) is often worse than for many solid tumors, underscoring the importance and urgency of the disease.[3] Therapies to prevent progression of CKD are mostly based on inhibition of the renin-angiotensin-aldosterone system, introduced >20 years ago, and on blockade of a sodium glucose transporter, introduced recently. Although these therapies clearly slow progression, not all CKD patients benefit to the same degree and even non-responders are emerging. CKD remains a major unmet medical need, for which therapeutics are desperately needed.

One critical limitation has been that animal models poorly recapitulate human diabetic kidney disease (DKD). Most strains of mice when made diabetic (e.g., by streptozotocin injection) do not develop phenotypes observed in patients with DKD, such as mesangial expansion, glomerular basement membrane thickening, tubulointerstitial damage, and endothelial hyalinosis.[4] Animal models often do not show progressive kidney function decline and other microvascular complications of diabetes such as hypertension and heart failure. DKD is associated with reduced nitric oxide (NO) bioavailability and endothelial dysfunction, similar to other cardiovascular disorders such as hypertension, heart failure, and metabolic syndrome.[5,6]

NO-soluble guanylate cyclase-cyclic guanosine monophosphate (NO-sGC-cGMP) signaling plays a critical role in regulating renal function.[7,8] Defects in NO availability (e.g., endothelial NO synthase [eNOS] deletion) can lead to severe kidney function deterioration and CKD.[8] Endogenous NO is generated from L-arginine by eNOS. After release from the endothelium, NO binds to sGC, which is a heterodimeric enzyme consisting of an α and β subunit carrying an N-heme-NO binding domain. The major isoforms are sGCα1β1 and sGCα2β1 encoded by *GUCY1A1*, *GUCY1B1*,

This is an open access article under the CC BY license (http://creativecommons.org/licenses/by/4.0/).

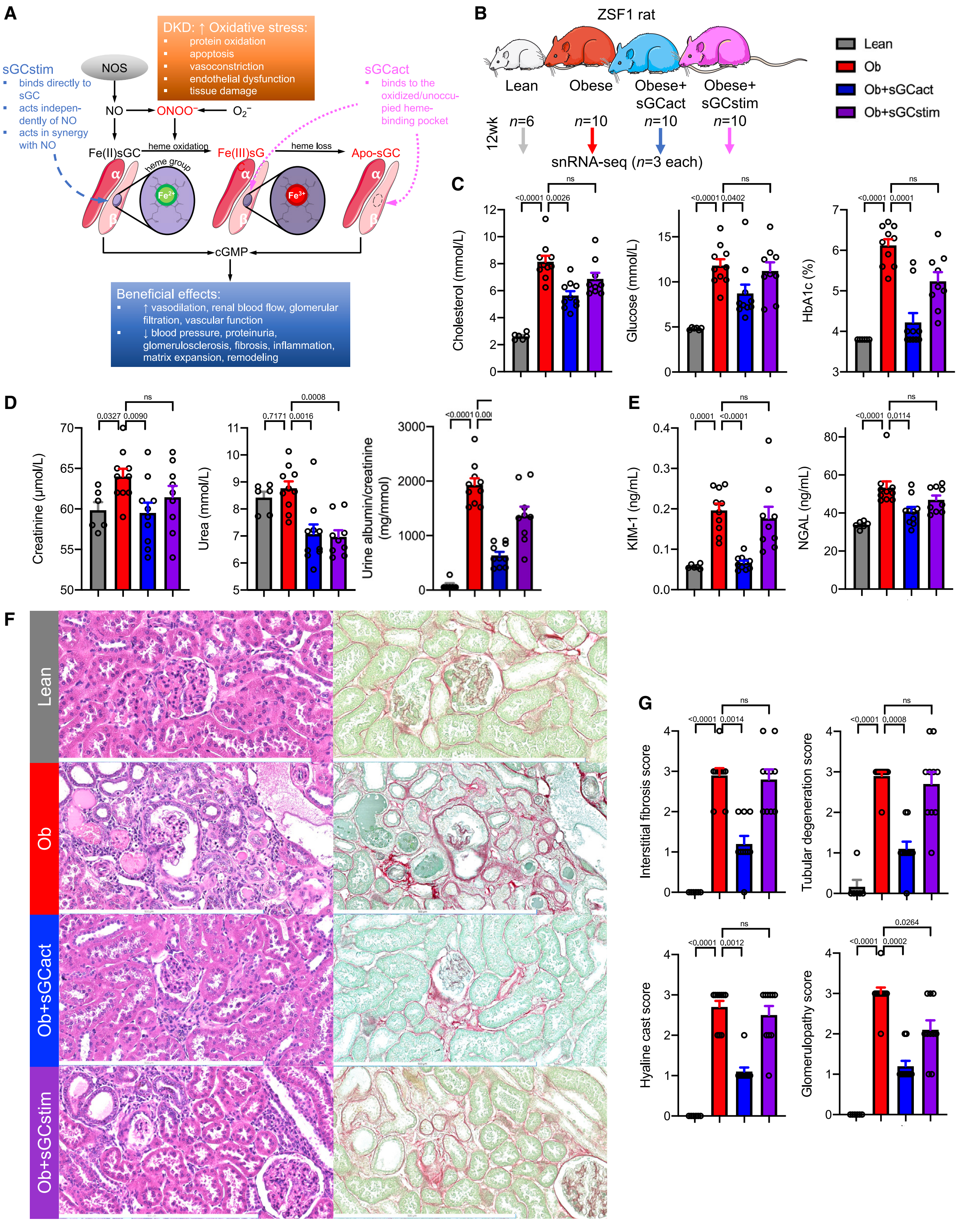

(legend on next page)

and *GUCY1A2*, respectively. NO-dependent sGC stimulation triggers formation of cGMP, which is the cellular second messanger.[9] sGC is therefore a key signal transducer of NO-mediated organ effects. NO-sGC-cGMP signaling can be impaired by increased reactive oxygen species production, scavenging of NO via the reaction of NO and O^{2-} to form peroxynitrite, or direct scavenging by free hemoglobin but also by oxidation of Fe^{2+} (ferrous) sGC to its NO-insensitive Fe^{3+} (ferric) state.[10,11] Moreover, sGC transcription and mRNA stability are affected by oxidative stress.[12]

Agonizing sGC directly has become a promising therapeutic approach. Current sGC agonists are categorized into two distinct classes based on their molecular mode of action. sGC stimulators (sGCstim) propel cGMP formation by binding to sGC, allosterically to the N-NOX domain, NO-independently and synergistically with NO. However, their efficacy depends on the ferrous state of the prosthetic heme group. In contrast, sGC activators (sGCact), by binding to the H-NOX domain directly, induce cGMP production preferentially at the oxidized/heme-free apo form of the enzyme, which is no longer responsive to NO and sGC stimulators[9,13] (Figure 1A). Hence, maintaining sGC heme in the ferrous state is essential for sGC-cGMP signaling via NO and sGCstim, whereas sGCact can act independently of the ferrous heme group, bound to the β1 subunit (encoded by *GUCY1B1*), potentially explaining higher sGCact activity under pathophysiological and high oxidative stress conditions, such as DKD, compared with sGCstim.[14]

Both sGCstim and sGCact have shown kidney-protective effects in preclinical CKD and DKD models[8,15–18] and have been advanced to clinical studies.[9] Despite the positive effect of sGC modulation on clinical outcomes, the target cell types and molecular mechanism of action for sGC are poorly understood. Here, we studied the pharmacological effects of sGCstim and sGCact in the ZSF1 rat as a representative DKD model at the single-cell level in the kidney. Through unbiased tensor decomposition analysis, we prioritize podocytes, proximal tubule (PT) cells, and stromal cells as most disease-relevant cell types in DKD and describe the latter two as sGC-expressing cells. We highlight a continuous transcriptional lineage relationship of PT and stromal cells, starting with differentiated PT cells to injured (PTinj) and profibrotic PT (ProfibPT) states toward mesenchymal cells (Mesench). Finally, we use unbiased weighted gene correlation network analysis (WGCNA) to build a score, which successfully stratified 991 human kidney bulk RNA sequencing (RNA-seq) samples by DKD prevalence, and functional (degree of proteinuria, glomerular filtration rate) and structural (fibrosis) kidney impairment.

RESULTS

Diabetic ZSF1 rats recapitulate phenotypic changes of DKD with marked disease improvement by sGC activators

It has been suggested that the obese ZSF1 rat model exhibits many of the phenotypic characteristics of human DKD, such as proteinuria, structural renal lesions, hyperglycemia, dyslipidemia, hypertension, oxidative stress, and obesity.[18–22] We analyzed ZSF1 obese diabetic rats at 25–26 weeks of age (Figure 1B). In line with previous publications, diabetic ZSF1 rats demonstrated marked obesity, hypercholesterolemia, hyperglycemia, elevated hemoglobin A1c (HbA1c), and hypertension (Figures 1C and S1A; Table S1), reflecting the pronounced metabolic disturbances reminiscent of the metabolic syndrome in humans.[23,24] Obese ZSF1 rats demonstrated impaired kidney function, as measured by elevated serum creatinine and urea, as well as marked proteinuria and albuminuria (Figures 1D and S1B). We noted higher levels of circulating kidney injury markers such as kidney injury molecule 1 (KIM-1)[25] and neutrophil gelatinase-associated lipocalin (NGAL)[26] (Figure 1E) in diabetic ZSF rats. We performed explorative proteomics analysis of 92 plasma proteins using a multiplexed proximity extension assay (Olink) (Data S1). Plasma proteins showing higher levels in diabetic rats included Delta-like 1 (DLL1) and ectodysplasin A2 receptor (EDA2R) (Figure S1C), both of which were recently found in a human proteomics study analyzing four independent cohorts of individuals with type 1 and type 2 diabetes and early and late DKD to be associated with progression to kidney failure.[27] Of the 46 proteins that Kobayashi et al. reported to be strongly associated with progression to kidney failure,[27] eight were included in our Olink panel. Interestingly, the levels of all eight proteins (100%) were significantly higher in diabetic ZSF1 rats (Data S1), again underscoring the similarities of the ZSF1 rat model to human DKD. These proteins had diverse biological functions including development (DLL1, MATN2), inflammation (EDA2R, IL17F, CCL5, TNFSF12), and transforming growth factor β (TGFβ) signaling (FSTL3, TGFBR3). Functional impairment in diabetic ZSF1 rats was mirrored by renal histopathological changes such as increased interstitial fibrosis, tubular degeneration, hyaline cast formation, and glomerulopathy (Figures 1F and 1G).[28,29]

Next, we aimed to characterize the effects of sGCact and sGCstim on renal and metabolic parameters of ZSF1 rats. While sGCact significantly alleviated metabolic changes, sGCstim did not (Figure 1C). The degree of renal function improvement was similar (as measured by urea) or greater (as measured by

Figure 1. Diabetic ZSF1 rats recapitulate phenotypic changes of DKD with marked disease improvement by sGC activators

(A) Representation of the importance of heme-containing (native) sGC and heme-free (dysfunctional) form of sGC and its redox equilibrium. sGC stimulator efficacy depends on the ferrous, Fe(II), state of the heme group at the β subunit of sGC, while sGC activators bind directly to oxidized, Fe(III), or heme-free apo form of sGC. Similar to other cardiovascular disorders, DKD is associated with reduced NO bioavailability, increased oxidative stress, and endothelial dysfunction. cGMP, cyclic guanosine monophosphate; DKD, diabetic kidney disease; NO, nitric oxide; NOS, nitric oxide synthase; O_2^-, superoxide; $ONOO^-$, peroxynitrite; sGCact, soluble guanylate cyclase activator; sGCstim, soluble guanylate cyclase stimulator. Adapted from Sandner et al.[9]

(B) Experimental ZSF1 rat model setup. sGCact, soluble guanylate cyclase activator; sGCstim, sGC stimulator.

(C–E) Metabolic (serum cholesterol, glucose, and plasma HbA1c) (C), kidney function (D), and kidney injury markers (E) after 12 study weeks; p values are given for either one-way ANOVA or Kruskal-Wallis test (both Benjamini, Krieger, Yekutieli corrected). Ob, obese; ns, not significant. Color legend as in (B).

(F) Histopathology changes in hematoxylin/eosin (left) and Sirius red/fast green (right) stained kidney sections. Scale bars, 500 μm.

(G) Histopathology scoring; p values are given for Kruskal-Wallis test (Benjamini, Krieger, Yekutieli corrected). Color legend as in (B).

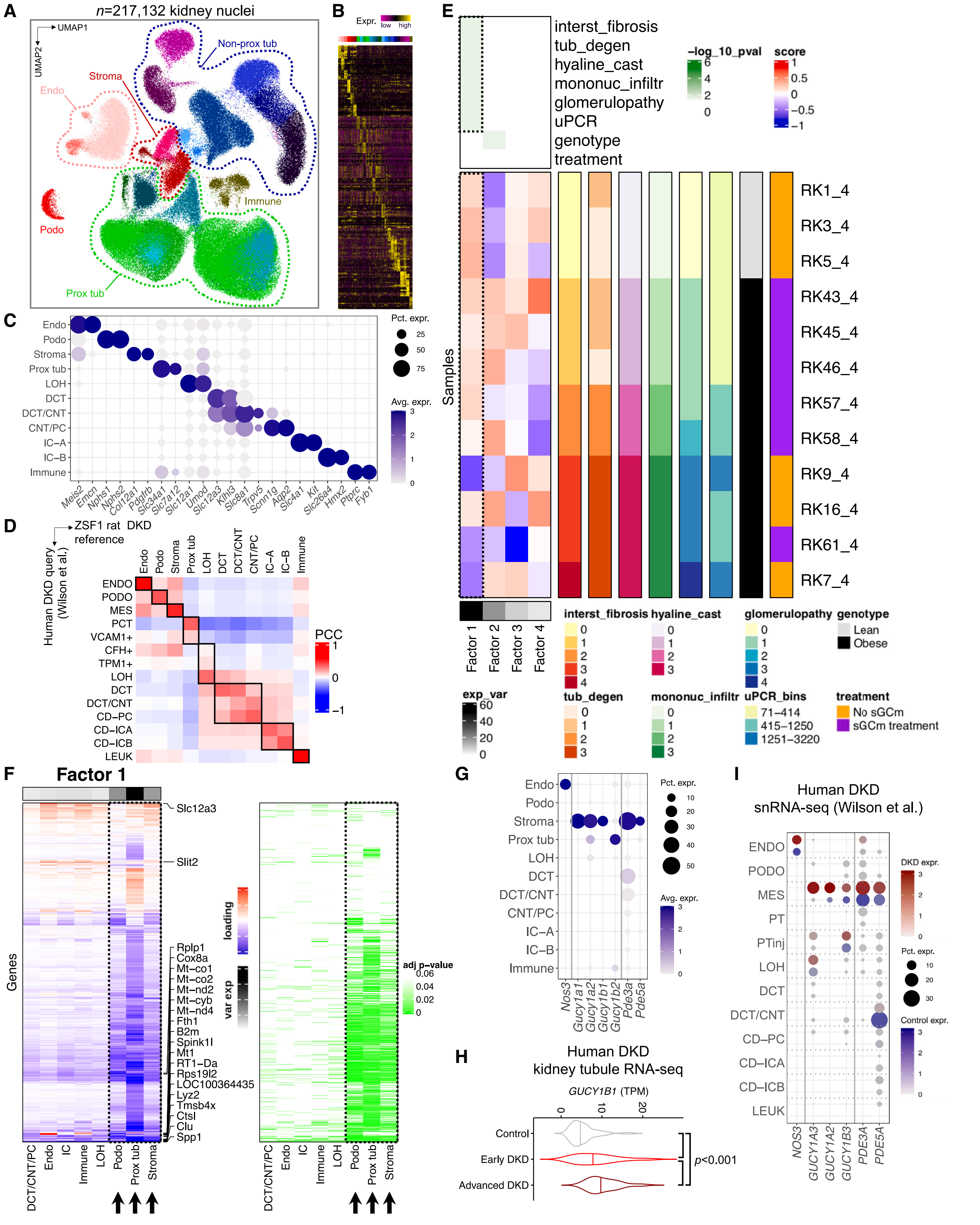

(legend on next page)

creatinine, albuminuria, and proteinuria) upon treatment with sGCact than with sGCstim (Figure 1D). Increased kidney injury and kidney disease progression markers were largely rescued by sGCact but not by sGCstim (Figures 1E, 1F, S1B, and S1C). Both tubulointerstitial and glomerular histopathological changes were markedly lower upon sGCact treatment, whereas we only saw a reduction in glomerulopathy upon sGCstim treatment (Figures 1F and 1G).

In summary, we found that obese, diabetic ZSF1 rats recapitulated functional and renal histopathological changes of human DKD. Pharmacological sGCact ameliorated functional and histological changes of DKD, while sGCstim had modest effects on ZSF rats.

Single-cell transcriptomic landscape of the diabetic ZSF1 rat kidney

To elucidate key cell types and DKD driver pathways, we next performed single-nuclei RNA-seq (snRNA-seq) on three rat kidney samples per group. After stringent quality control of each individual sample, including ambient RNA correction, doublet removal, nuclei filtering based on UMI, counts, and mitochondrial percentage (STAR Methods and Figures S2A–S2D), we integrated transcriptomes of high-quality single cells into a single dataset following batch correction (STAR Methods) and retained 217,132 high-quality single kidney nuclei (Figures 2A and S2E). Unsupervised clustering indicated 25 cell clusters (Figures 2B and S2E). After cluster-specific differential gene-expression analysis (Figure S2f, Data S2), we grouped clusters into coarse-grained, high-level cell types: podocytes (Podo), endothelial cells (Endo), stroma cells (Stroma), proximal tubule cells (Prox tub, PT), non-proximal tubule cells (Non-prox tub) such as loop of Henle (LOH), distal convoluted tubule (DCT), connecting tubule (CNT), collecting duct principal cells (PC), and collecting duct intercalated cells (IC), as well as immune cells (Immune) (Figures 2A and 2C). Each cell type was present in every sample and in all groups, indicating the lack of major batch effect and negligible within-group heterogeneity (Figures S3A–S3C). Differential proportion analysis showed significant differences in cell fractions between groups for almost all cell types ($p < 0.001$ for obese vs. lean comparisons in Endo, Stroma, Prox tub, Non-prox tub, and IC; $p < 0.05$ for Immune; not significant for Podo) (Figure S3D). Next, we identified differentially expressed genes (DEGs) between disease states and treatment groups. PT and stromal cells showed the highest number of DEGs between treatment groups (Figures S4A–S4C; Data S3, S4, and S5). Importantly, individual cell-cluster transcriptomes in our ZSF1 rat DKD model demonstrated strong correlation with corresponding cell-cluster transcriptomes in two independent human single-cell DKD datasets[30] including the Kidney Precision Medicine Project (KPMP)[31] and served as an excellent reference on which all cell types present in the human DKD query dataset could be projected with high prediction accuracy (Figures 2D and S5A–S5D).

As the disease state was associated with important differences in both cell fractions and cell-type-specific gene expression, we used tensor decomposition analysis on our single-nuclei dataset (Figure 2E; Data S6, S7, and S8) for an unbiased determination of critically important cell types associated with phenotypic changes. High-level cell-type identity, histopathological, and proteinuria metadata served as input for this unsupervised analysis that allowed us to retrieve main factors associated with phenotypic outcomes of the respective samples in an unbiased manner. Factor 1 explained by far the most (48.7%) transcriptomic variation across all samples and was significantly associated with interstitial fibrosis, tubular degeneration, hyaline cast formation, glomerulopathy, and proteinuria. Consistently, untreated ZSF1 obese samples had the lowest factor 1 scores, the highest proteinuria levels, and most severe histological damage. Factor 2 explained 19.0% of variation and was associated with rat genotype (ZSF1 lean vs. ZSF1 obese), suggesting that

Figure 2. Single-cell transcriptomic landscape of the diabetic ZSF1 rat

(A) Integrated UMAP of 217,132 high-quality nuclei from twelve rat kidney samples; Endo, endothelial cells; Immune, immune cells; Non-prox tub, non-proximal tubule; Podo, podocytes; Prox tub, proximal tubule; Stroma, stromal cells.

(B) Heatmap of top ten differentially expressed genes for low-level clustering.

(C) Marker gene expression for high-level clustering. Dot size denotes percentage of cells expressing the marker. Color scale represents average gene-expression values.

(D) Pearson correlation coefficient (PCC) matrix of average cell type gene expression between ZSF1 rats (lean and obese samples only) and a corresponding human snRNA-seq dataset with control and DKD kidney samples. CD-ICA/IC-A, collecting duct intercalated cell type A; CD-ICB/IC-B, collecting duct intercalated cell type B; CD-PC/PC, collecting duct principal cell; CNT, connecting tubule; DCT, distal convoluted tubule; Endo/ENDO, endothelial cell; LEUK, leukocyte; LOH, loop of Henle; MES, mesangial cells; PCT, proximal convoluted tubule; Podo/PODO, podocytes; Prox tub, proximal tubule.

(E) Tensor decomposition analysis heatmap (center left) representing factor loading score of rat kidney (RK) samples (rows) onto tensor factors (rows). Degree of explained variance (exp_var) in the whole dataset is displayed on the bottom left. Significance level ($-\log_{10}$(p value)) of tensor factor association with clinical (uPCR, urinary protein/creatinine ratio in mg/mmol) and histopathology outcome measures (interst_fibrosis, interstitial fibrosis; tub_degen, tubular degeneration; mononuc_infiltr, mononuclear infiltration, glomerulopathy, each scored from 0 to 4) is displayed on the top left. Sample rows are color-annotated by outcome data, genotype (lean vs. obese), and treatment status (sGCm, sGC modulator treatment, or no treatment).

(F) Heatmap representing factor 1 loading scores by cell type (columns) and genes (rows) (left). Explained variance is colored in shades of gray (top left), significance levels are shown on the right. The top five significant genes for every cell cluster are annotated.

(G) Expression dot plot for NO/sGC/cGAMP pathway genes. Dot size denotes percentage of cells expressing the marker. Color scale represents average gene-expression values.

(H) Expression of *GUCY1B1* in human microdissected kidney tubule bulk RNA-seq samples, stratified by control, early DKD, and advanced DKD cases; p value is given for one-way ANOVA (Tukey corrected). TPM, transcripts per million.

(I) Expression dot plot for NO/sGC/cGAMP pathway genes in a human DKD snRNA-seq dataset. Dot size denotes percentage of cells expressing the marker. Red and blue color scales represent average gene-expression values in DKD and control samples, respectively; PTinj, injured PT (composite of VCAM1$^+$, CFH$^+$, TPM1$^+$ PT cells).

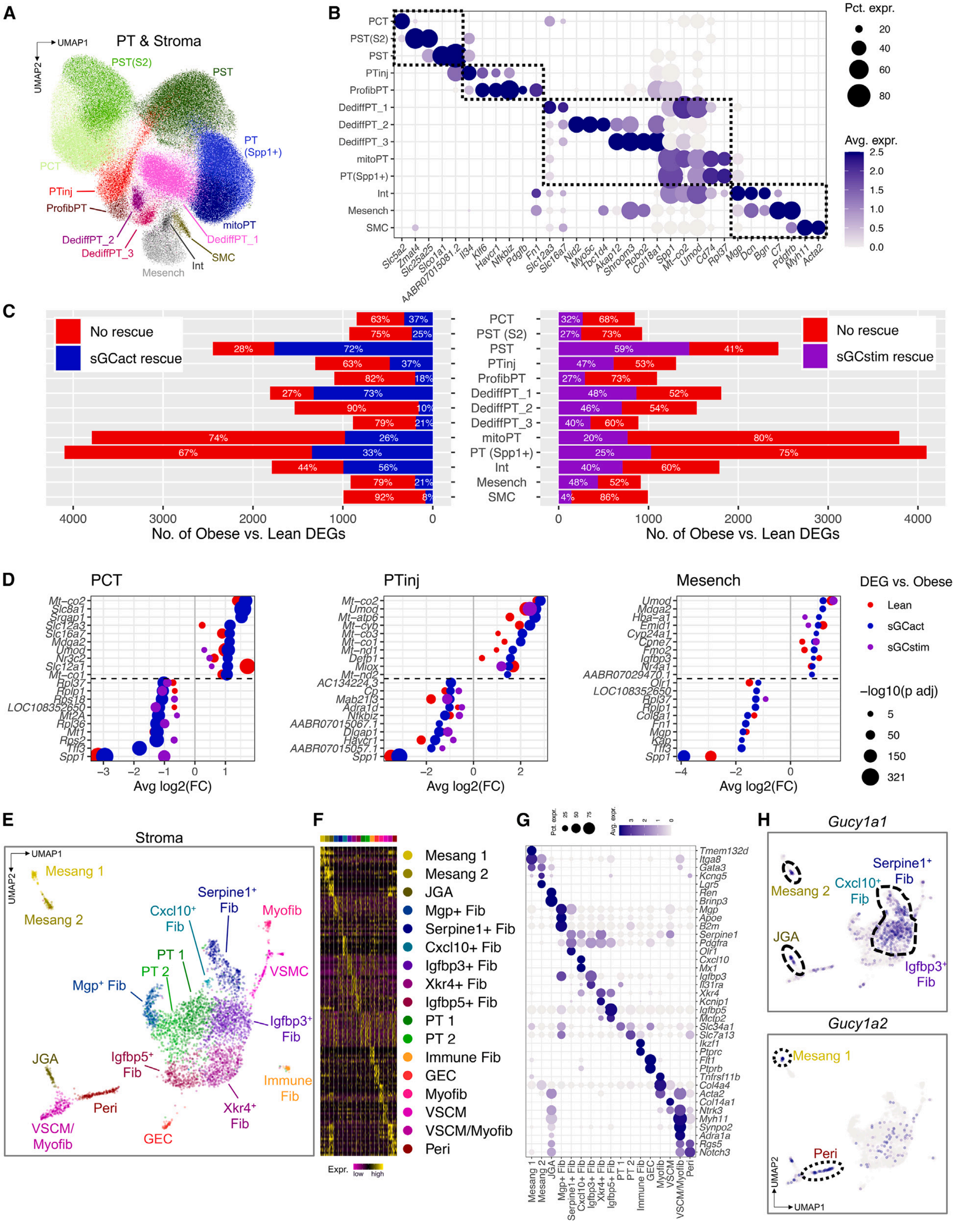

(legend on next page)

the phenotype was a more important determinant of gene expression than the genotype. More detailed analysis of factor 1 loadings revealed that the majority of genes loading onto factor 1 were specific to three cell types: podocytes, PT, and stromal cells (Figure 2F). Gene ontology (GO) analysis of factor 1-loading genes in PT and stromal cells revealed that repair (e.g., wound healing, regeneration, negative regulation of cell adhesion) and electron transport processes (e.g., mitochondrial respiratory chain complex assembly, electron transport chain, ATP metabolic process, proton transmembrane transport) were the top enriched pathways (Figure S6A).

As we sought to study effects of pharmacological sGC modulation, we were reassured to find sGC genes (*Gucy1a1*, *Gucy1a2*, *Gucy1b1*, *Gucy1b2*) to be expressed almost exclusively in PT and stromal cells (Figures 2G, S6B, and S6C). PT and stroma specificity for sGC and downstream cGMP effectors such as *PDE3A* and *PDE5A* was also observed in recent human and mouse DKD snRNA-seq datasets[30,32] (Figure S6D), suggesting conservation across species. In addition, microdissected kidney tubule RNA-seq samples from human individuals with advanced DKD showed higher sGC mRNA expression (Figure 2H). Finally, reanalysis of a human DKD snRNA-seq dataset[30] confirmed increased sGC expression in DKD stroma compared with control stroma (annotated as "MES," mesangial, by the authors) as well as expression in PTinj (Figure 2I).

Pharmacological sGC modulation improves gene expression in multiple cell types

As our unsupervised tensor decomposition analysis prioritized podocytes, PT, and stromal cells as key cell types for improved structural and functional outcome, we focused on these cells. After three iterative rounds of clustering, we subset 2,065 podocyte nuclei that formed five clusters, establishing a continuous trajectory (Figures S7A and S7B). Pathway enrichment analysis demonstrated that the start of the trajectory (Podo1–Podo3) was defined by nephrin, glomerular epithelium, glomerular development, or actin filament pathways, which is typical for healthy podocytes. Cluster Podo4 was specifically enriched for, e.g., FAK, p53, and apical junction pathways, whereas cluster Podo5 (at the end of the trajectory) was enriched specifically for, e.g., oxidative phosphorylation, ribosomal, and glutathione metabolism pathways (Figure S7C). Clusters Podo1–Podo4 positively correlated with each other (Figure S7D). Diabetic ZSF1 obese rats showed considerably lower fractions of differentiated Podo1 nuclei, which was rescued by sGCact but not by sGCstim (Figure S7E). Vice versa, Podo5 was lowest in sGCact-treated rats and was enriched for oxidative phosphorylation (Figures S7E and S7F).

Next, we turned to PT and stromal cells, which demonstrated proximity in uniform manifold approximation and projection (UMAP) space (Figure 2A), suggesting a close and potentially continuous transcriptomic relationship. We therefore chose to analyze these two cell types together. After subclustering, we retrieved 13 cell clusters (Figures 3A and S8A–S8G). Based on the high expression of their cluster-specific DEGs (Figure 3B and Data S9) we annotated them as proximal convoluted tubule (PCT, *Slc5a2*), proximal straight tubule S2 segment (PST S2, *Zmat4*, *Slc25a25*), and proximal straight tubule (PST, *Slc1a1*), which represented healthy PT cells with typical marker gene expression. Other clusters included injured PT (PTinj, *Il34*, *Klf6*, *Havcr1*) and profibrotic PT (ProfibPT, *Havcr1*, *Nfkbiz*, *Pdgfb*, *Fn1*), which shared most of the typical injury signature from previous literature.[33–35] Other clusters showed features of cellular dedifferentiation with low or absent expression of typical PT markers (DediffPT_1, *Slc12a3*, *Umod*; DediffPT_2, *Nid2*, *Myo5c*, *Tbc1d4*; DediffPT_3, *Akap12*, *Shroom3*, *Robo2*), high mitochondrial gene content (mitoPT, *Cd74*), or high osteopontin (PT Spp1$^+$, *Spp1*). A fourth group of cells represented interstitial (Int, *Mgp*, *Dcn*, *Bgn*), mesenchymal (Mesench, *C7*, *Pdgfrb*), and smooth muscle cells (SMC, *Myh11*, *Acta2*), respectively.

Next, we analyzed the number of DEGs "normalized" (their expression changed to healthy level) by sGCact and sGCstim treatment (Data S10, S11, and S12). We found that the fractions of DEGs normalized by sGC modulation were highly variable and cell-type specific (Figure 3C). The expression of a larger number of genes returned to baseline (healthy state) upon sGCact (n = 8,240) compared with sGCstim treatment (n = 7,885), which was consistent with the improved structural and functional outcome upon sGCact treatment (Figures 1C–1G). Obese ZSF1 rats (compared with lean) showed the highest numbers of DEGs in mitoPT, PT(Spp1$^+$), PST, and DediffPT_1. The largest numbers of genes returning to healthy control level by sGCact treatment were observed in DediffPT_1 (73% rescue), PST (72%), and Int (56%). For sGCstim treatment the highest percentages of rescue were observed in PST (59%), Mesench

Figure 3. Pharmacological sGC modulation improves gene expression in multiple cell types

(A and B) Integrated UMAP (A) and marker gene expression (B) for PT and stromal cell subclusters. PCT, proximal convoluted tubule; PST(S2), proximal straight tubule (segment 2); PST, proximal straight tubule; PTinj, injured PT; ProfibPT, profibrotic PT; DediffPT, dedifferentiated PT; mitoPT, high mitochondrial gene PT; PT(Spp1+), Spp1$^+$ PT; Int, interstitial cell; Mesench, mesenchymal cell; SMC, smooth muscle cell. Dot size (B) denotes percentage of cells expressing the marker. Color scale (B) represents average gene-expression values.

(C) Bar graphs representing the number of genes differentially expressed (DEGs) between obese and lean samples in PT and stroma subclusters. Percentages indicate absent or present rescue effect (normalization) for DEG comparison between sGC modulator-treated rats (sGCstim, sGCact) and vehicle-treated rats.

(D) Dot plots representing the effect size of DEG normalization by sGCact (blue) and sGCstim (purple) for proximal convoluted tubule (PCT), injured PT (PTinj), and mesenchymal cells (Mesench). The top ten upregulated and top ten downregulated genes are shown. x axis denotes the effect size of DEG rescue/normalization, dot size denotes significance level, color represents the effect of genotype (lean vs. obese) and pharmacological treatment (sGCact, sGCstim vs. obese), respectively.

(E–G) UMAP (E), top ten DEGs per cluster (F), and marker gene expression (G) for stromal cell subclusters. Mesang, mesangial cell; JGA, juxtaglomerular apparatus cell; Fib, fibroblast; PT, proximal tubule; GEC, glomerular endothelial cell; Myofib, myofibroblast; VSCM, vascular smooth muscle cell; Peri, pericyte. Dot size (G) denotes percentage of cells expressing the marker. Color scale (G) represents average gene-expression values.

(H) Feature plots for *Gucy1a1* and *Gucy1a2* in UMAP space.

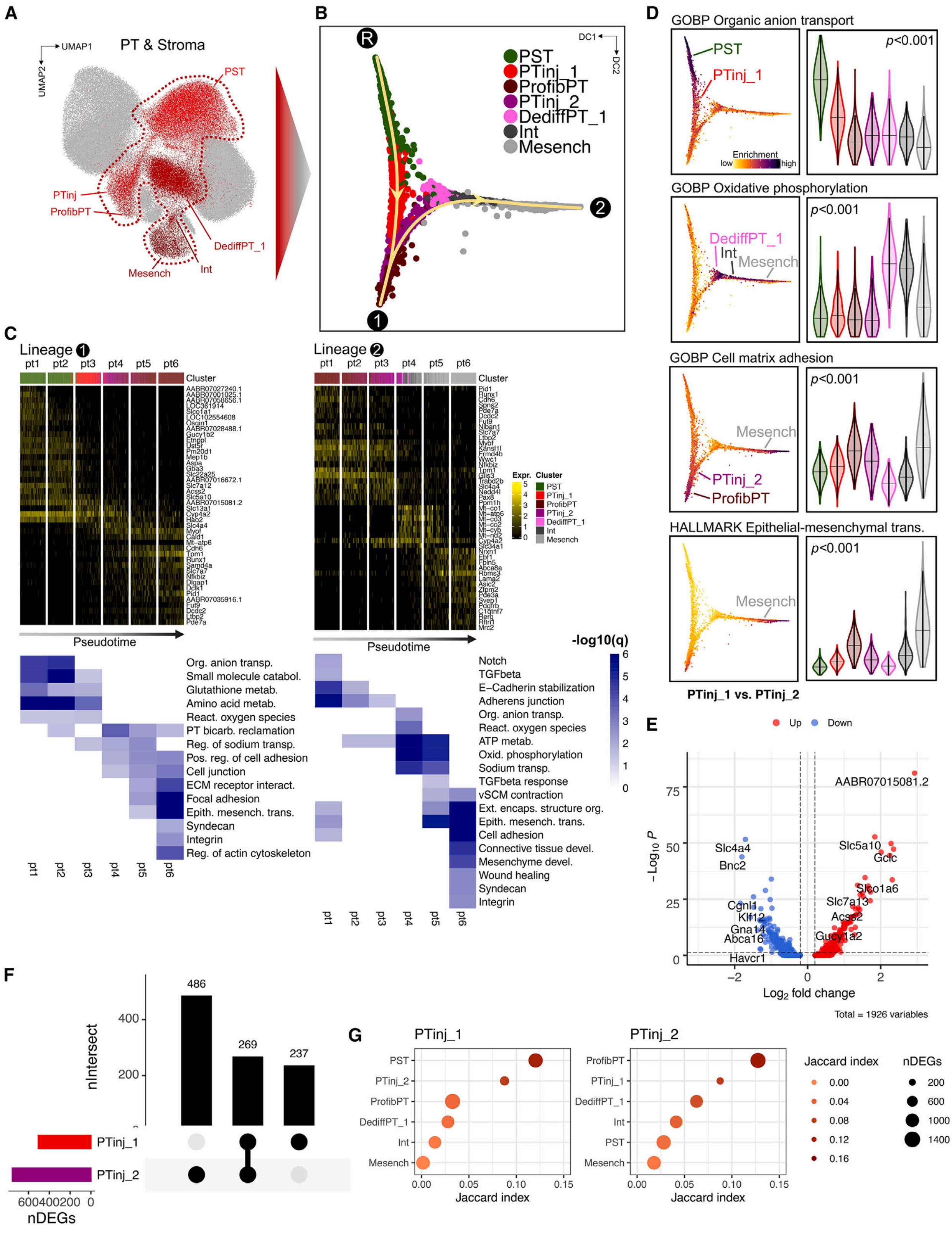

(legend on next page)

(48%), DediffPT_1 (48%), and PTinj (47%). While sGCact treatment was associated with a larger number of genes returning to baseline level than sGCstim treatment, we did not observe strong cell-type-specific differences between the two drugs, suggesting a class effect of action (Figures 3C and S8H). Finally, the effect size of top DEG normalization via sGCact was similar to that of a control (lean) genotype (Figure 3D), which again underlined the high effectiveness of sGCact.

We noticed a marked reduction of cGMP signaling in obese rats (compared with lean), which was restored more successfully by sGCact than sGCstim (Figure S9A). DKD is associated with endothelial dysfunction and increased oxidative stress due to NO depletion, leading to oxidized and heme-free sGC, which neither NO nor sGCstim can target.[9] We were therefore intrigued to find that sGCact preserved gene expression of markers associated with negative regulation of oxidative stress better than sGCstim treatment (Figure S9B). This could potentially explain—at least in part—the observed treatment benefits of sGCact over sGCstim. Along those lines, the negative oxidative stress regulation and cGMP effect sizes correlated positively (Figure S9C). We further validated these findings in an external bulk kidney RNA-seq dataset:[36] advanced DKD kidneys had significantly lower cGMP effects scores than early DKD kidneys ($p = 0.019$), while controls and early DKD kidneys were not different ($p = 0.085$) (Figure S9D). Similarly, kidneys from advanced DKD patients exhibited the lowest scores for negative regulation of oxidative stress compared with early DKD and control cases (Figure S9E). Again we found a positive correlation between oxidative stress response and cGMP effect size (Figure S9F) in human kidneys.

Our single-cell gene-expression data, consistent with prior immunostaining and *in situ* hybridization studies,[37] indicated sGC expression in stromal cells. To better understand sGC expression in the renal stroma, we subclustered the stromal cells. Unbiased clustering revealed two mesangial cell clusters (Mesang, *Itga8*, *Gata3*); juxtaglomerular apparatus (JGA, *Ren*); multiple fibroblast (Fib) clusters with previously described marker genes, such as *Mgp*, *Apoe*, *B2m*, *Serpine1*, *Pdgfra*, *Cxcl10*, *Igfbp3*, *Xkr4*, *Igfbp5* or with an immune cell signature (Immune Fib, *Ikzf1*, *Ptprc*); clusters with PT marker genes (PT1, PT2); glomerular endothelial cells (GEC, *Flt1*, *Ptprb*); myofibroblasts (Myofib, *Tnfrsf11b*, *Acta2*); vascular smooth muscle cells (VSMC, *Col14a1*, *Ntrk3*); a mixture of the latter two (VSMC/Myofib, *Ntrk3*, *Myh11*, *Synpo2*); and pericytes (Peri, *Rgs5*, *Notch3*) (Figures 3E–3G and Data S13). We noted the following patterns of expression of sGC pathway genes (Figures 3H, S9G, and S9H). Serpine1^{+} Fib, Cxcl10^{+} Fib, and Igfbp3^{+} Fib expressed both *Gucy1a1* and *Gucy1b1*, Mesang 2 and JGA enriched mainly for *Gucy1a1*, while *Gucy1a2* was mainly expressed in Mesang 1 and Peri. sGC expression was largely absent from VSMC and Myofib, although downstream effectors such as *Pde3a* and *Pde5a* were expressed in these cell types. This is largely consistent with prior analyses that have highlighted mesangial cells, JGA, Fib throughout the cortical labyrinth, and Peri as main sites of sGC expression.[37]

Taken together, DEG analysis with a focus on the proportion of rescued genes suggested high variability between cell types but with a larger number of genes returning to baseline with sGCact in comparison with sGCstim. Negative regulation of oxidative stress and downstream cGMP effects were better preserved upon sGCact treatment compared with sGCstim. We found that oxidative stress and cGMP effects correlated with clinical outcomes in both ZSF1 rats and DKD patients. Finally, we showed that sGC genes were mainly expressed in Mesang, JGA, Peri, and different Fib subsets, underscoring the importance of multiple stromal cells for sGC.

Trajectory analysis highlights dynamic changes of PT cells toward profibrotic and mesenchymal cell states

Our analysis consistently highlighted PT and stromal cells as potential disease-driving cell types (Figure 4A). Dimension reduction after diffusion mapping revealed two consecutive trajectories. Lineage 1 originated from the healthy root state (PST), via PTinj toward ProfibPT (Figure 4B). DEGs specifically higher along this trajectory (Data S14) enriched for typical PT functions such as organic anion transport, small molecule, and amino acid metabolism at the start of the lineage (PST) toward pathways associated with adherens junctions, extracellular matrix (ECM)-receptor interaction, focal adhesion, and epithelial-to-mesenchymal transition (EMT) at the end of the lineage (ProfibPT) (Figures 4C and 4D; Data S15). The second trajectory followed a path from ProfibPT, enriching for tight junction, TGFβ signaling, and adherens junction signaling, via a second PTinj cluster and DediffPT toward Int and Mesench, enriched, e.g., for EMT, cell adhesion, collagen fibril organization, and wound healing (Figures 4B–4D and Data S15). To understand the stability and reproducibility of this trajectory, we used two different orthogonal methods (monocle2, monocle3) and obtained similar cell-trajectory profiles (Figures S9I–S9N). Interestingly, DEG analysis revealed that PTinj cells separated into two

Figure 4. Trajectory analysis highlights dynamic changes of PT cells toward profibrotic and mesenchymal cell states

(A and B) Representative healthy and injured PT as well as stroma cell clusters subjected to trajectory analysis in UMAP (A) and diffusion map space (B). R, root state; 1, endpoint of lineage 1; 2, endpoint of lineage 2; PST, proximal straight tubule; PTinj, injured PT; ProfibPT, profibrotic PT; DediffPT, dedifferentiated PT; Int, interstitial cell; Mesench, mesenchymal cell.

(C) Top heatmaps showing generalized additive modeling (GAM)-derived DEGs along lineage 1 (R→1) and lineage 2 (1→2). Rows represent DEGs, columns represent individual PT cells in bins along pseudotime. Color legend at the top corresponds to clusters from (B). Bottom heatmaps show corresponding enrichment of top pseudotime-specific GO biological processes and KEGG pathways.

(D) Scoring of gene sets corresponding to representative pathways from (C). Left panels show pathway enrichment along the trajectory. Right panels show gene set scores by cell type. p values are given for one-way ANOVA (Tukey corrected); violin colors correspond to cell clusters in (B) along the trajectory.

(E) DEGs between PTinj_1 (lineage 1) and PTinj_2 (lineage 2).

(F) Upset plot of DEGs for PTinj_1 vs. PTinj_2.

(G) Similarity measured by Jaccard index. PTinj_1 was most similar to healthy PST, while PTinj_2 was most similar to ProfibPT. Dot size denotes the number of DEGs, color denotes degree of similarity.

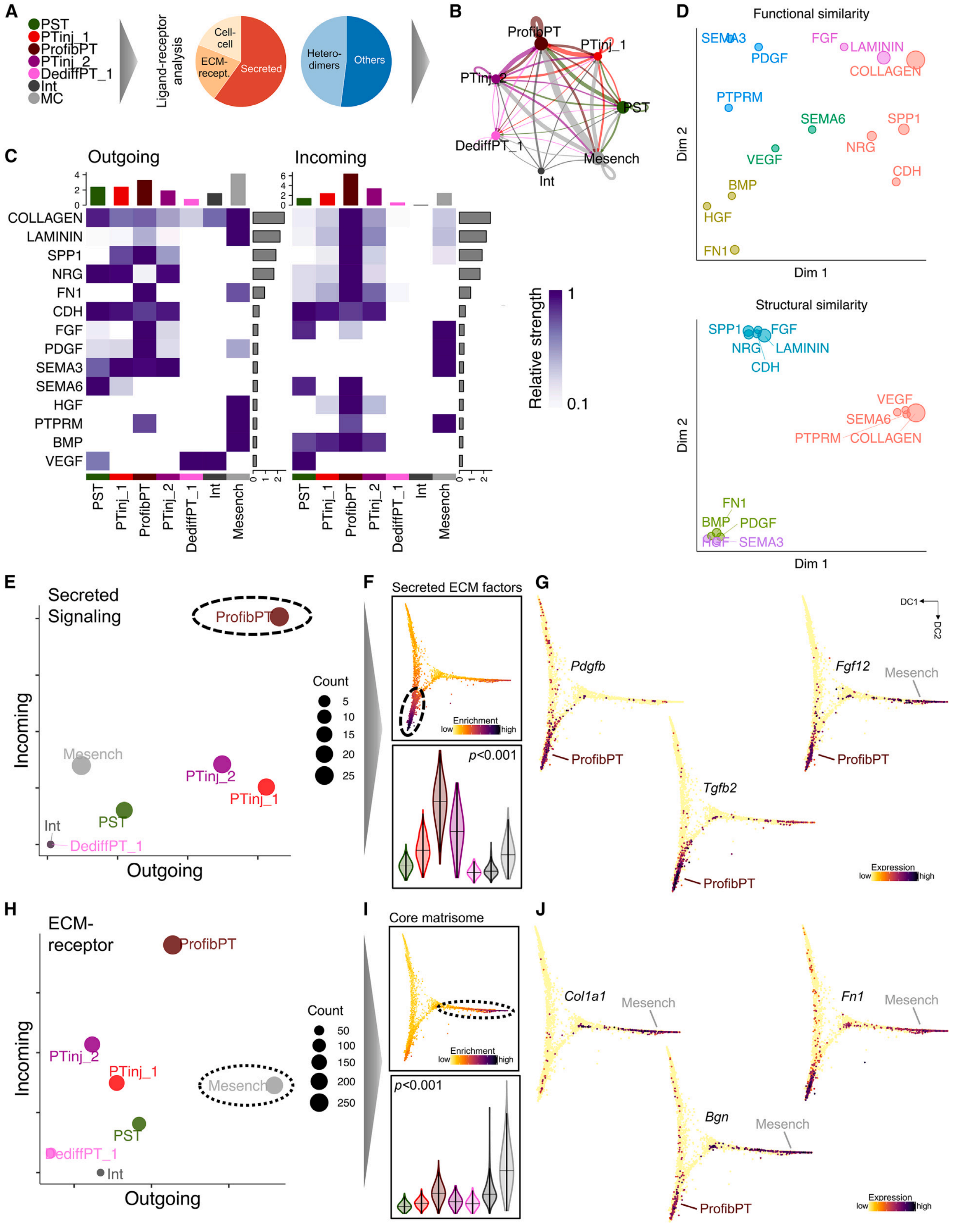

(legend on next page)

CellPress
OPEN ACCESS

clusters on either lineage 1 or lineage 2 (Figure 4E). These two subgroups showed a large number of non-overlapping, i.e., individually unique, DEGs (Figure 4F and Data S16), indicating separate transcriptomic states. Genes in the transcriptomic state of PTinj_1 were enriched for pathways such as cellular amino acid metabolism, oxidative phosphorylation, and organic anion transport (Figure S9O). These genes are essential for healthy PT function. PTinj_2 was enriched for VSMC migration, positive regulation of cell adhesion, and external encapsulating structure organization, and hence was more similar to a stromal identity of lineage 2 (Figure S9P). Jaccard similarity analyses of cluster-specific DEGs confirmed the highest overlap of PTinj_1 with healthy PST, whereas overlap of PTinj_2 was highest with ProfibPT, respectively (Figure 4G), again highlighting stark transcriptional differences between these two PTinj states. This was validated by clear separation of GO terms enriched in PTinj_1 and PTinj_2, respectively, in latent semantic space (Figure S9Q and Data S17).

In summary, we demonstrate the close transcriptional relationship of PT and stromal cell types. Upon injury in the diabetic ZSF1 rat model, PT cells adopted a profibrotic and mesenchymal transcriptome.

Cell-cell communication analysis identifies a secretory phenotype of profibrotic PT

As epithelial/stromal interplay has previously been shown to be implicated in kidney disease development, we next performed ligand-receptor analysis in PT and stromal subclusters (Figure 5A). ProfibPT and Mesench clusters presented with the highest interaction weights (Figure 5B) and showed the highest ECM signaling ligand expression (Figure 5C). ProfibPT and PTinj_2 expressed the most ECM receptors (Figures 5C, S10A, and S10B). The captured ligand-receptor network was functionally diverse (Figure 5D) and we could attribute separate patterns (Figure S10C): ProfibPT exhibited a strong secretory phenotype (Figure 5E) and scored highly for secreted ECM factors (Figure 5F), such as *Pdgfb*, *Tgfb2*, *Fgf12*, *Hbegf*, *Il19*, and *Il24* (Figures 5G, S11A, and S11B). Moreover, Mesench was associated with the strongest ECM-associated outgoing signal (ligand expression) (Figure 5H) and scored highest for the core matrisome (Figure 5I), as reflected by high expression of *Col1a1*, *Col3a1*, *Bgn*, *Prelp*, *Fbln5*, and *Fn1* (Figures 5J, S11C, and S11D).

Gene-regulatory network analysis highlights cell-type-specific transcription factors driving the PT-to-Mesench trajectory and prioritizes cell types of action for sGC modulation

To gain insight into putative driver transcription factors (TFs) of the PT-to-Mesench cell trajectory, we performed gene-regulatory network (GRN) analysis (Figure 6A). The root state and endpoints of lineages 1 and 2 showed the highest regulon density (Figures 6B, S12A, and S12B), again underscoring the richness within the transcriptomic states of differentiated PST, ProfibPT, and Mesench, respectively. The GRN logic that we inferred from *cis*-regulatory motif analysis clearly demonstrated that binarized regulon activity was able to independently differentiate and cluster all cells along the trajectory (Figure 6C), suggesting high data quality and validating our prior clustering and trajectory analysis results. For example, we found highly specific regulons for PST (*Gcm1*, *Stat5a*, *Bcl6*, *Lmx1b*, *Trps1*), ProfibPT (*Tead2*, *Bach2*, *Stat3*, *Gli3*, *Fosl2*), and Mesench (*Gli2*, *Gata6*, *Fli1*, *Tcf7l2*, *Hoxc6*) (Figures 6D, S12C, and S12D; Data S18). Taken together, our GRN analysis confirmed many known key TFs important for kidney disease development and attributed specific cell types to them. We also found novel cell-type-specific TFs, such as *Nr1h4* for PTinj_1, *Nfyc* for DediffPT_1, and *Foxn3* for PTinj_2, which are interesting candidates for studying their roles in renal disease development and warrant validation in future studies.

Finally, we asked whether we could infer specific cell types of action for sGC modulator treatment from our GRN. To this effect, we filtered for TFs that were predicted to target sGC genes (Figure 6E). We found marked enrichment of these regulons in PST, ProfibPT, and Mesench, with the highest regulon specificity scores for TFs *Gcm1*, *Zmiz1*, and *Srebf2*, respectively, targeting either *Gucy1a2* or *Gucy1b2* (Figure 6F). Reassuringly, *Zmiz1* was the top specific TF for ProfibPT. We have shown in a recent expression quantitative trait loci meta-analysis in human microdissected kidneys that *ZMIZ1* is an eGene associated with several genome-wide association study variants significantly associated with kidney function.[38] ZMIZ1 has already been identified to be strongly associated with ESRD attributed to type 1[39] and type 2 diabetes.[40] Along those lines, regulon cell-type specificity tracked TF expression: *Gcm1* enriched only in healthy PST, *Zmiz1* in ProfibPT, and *Srebf2* in Mesench (Figures 6G and S12E). We found sGC genes to be expressed in PST, PTinj_1,

Figure 5. Cell-cell communication analysis identifies a secretory phenotype of profibrotic PT

(A) PT-Mesench trajectory clusters from Figure 4 were subjected to ligand-receptor analysis. Cell-cell interactions comprised secreted, ECM-receptor, and direct cell-cell interactions.

(B) Weighted total interaction strength. Line size denotes interaction strength, color represents cell clusters from (A).

(C) Relative strength of outgoing and incoming interaction signaling is summed up for clusters along the trajectory (columns) as well as summed up and ranked by contributing pathway (rows).

(D) Dimension reduction visualizing functional and structural similarity of contributing signaling pathways in all clusters of the trajectory.

(E) The number of incoming and outgoing secreted signaling connections indicates the secretory phenotype of ProfibPT.

(F) Scoring of gene sets corresponding to secreted ECM factors. Top panel shows pathway enrichment along the trajectory. Bottom panel shows secreted ECM factor scores by cell type. p value is given for one-way ANOVA (Tukey corrected).

(G) Feature plots for representative secreted ECM factor genes (*Pdgfb*, *Tgfb2*, *Fgf12*) along the trajectory in diffusion map space.

(H) The number of incoming and outgoing ECM-receptor connections indicates the strong matrisome signature of Mesench.

(I) Scoring of gene sets corresponding to the core matrisome. Top panel shows pathway enrichment along the trajectory. Bottom panel shows core matrisome scores by cell type. p value is given for one-way ANOVA (Tukey corrected).

(J) Feature plots for representative core matrisome genes (*Col1a1*, *Bgn*, *Fn1*) along the trajectory in diffusion map space.

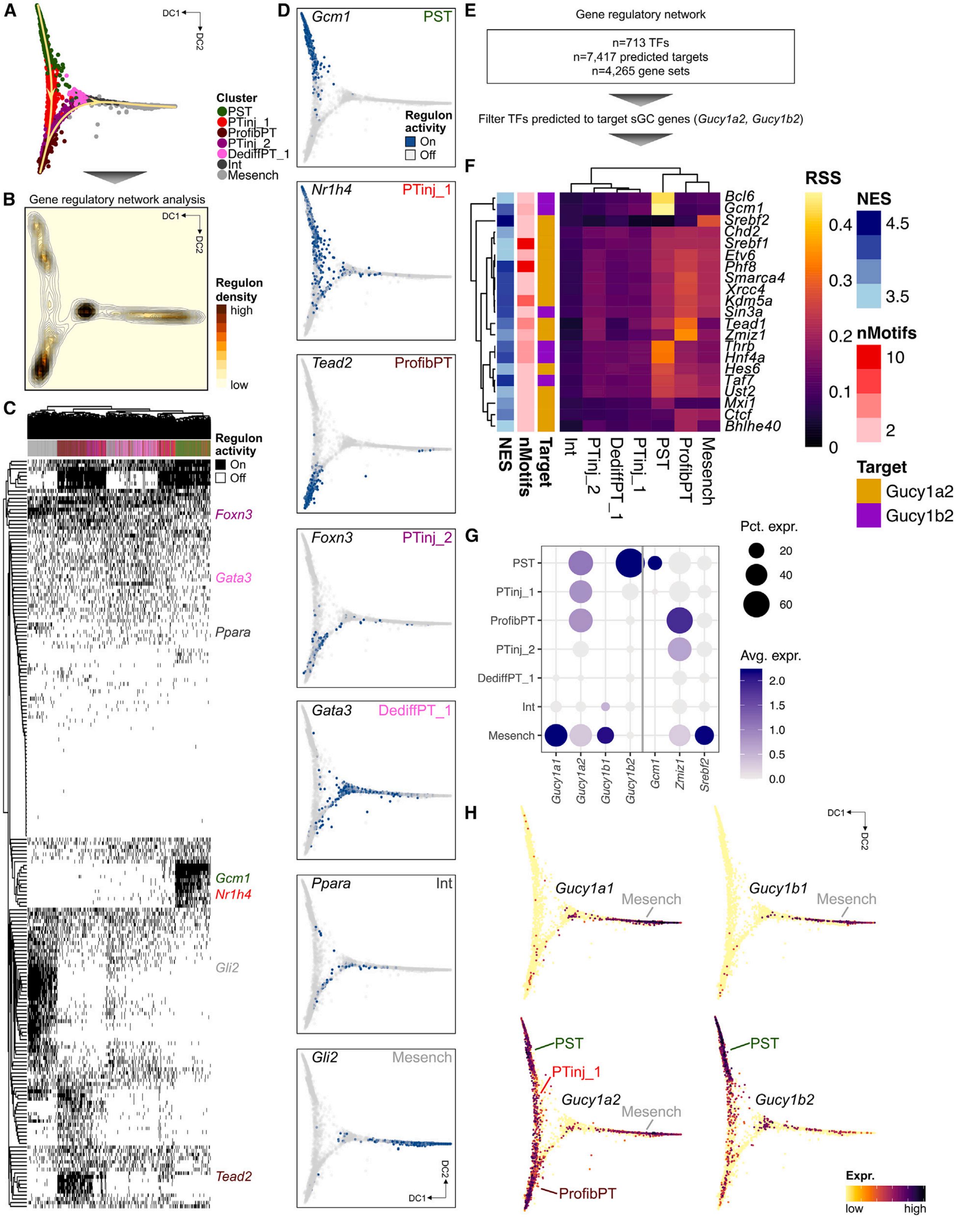

(legend on next page)

ProfibPT, and Mesench (Figure 6H), further highlighting these probable cell types of action for pharmacological sGC modulation. To our knowledge, our report is the first to attribute cell-type specificity of ZMIZ1 to ProfibPT, linking genetic discoveries with functional studies, and warrants validation in future studies.

WGCNA-derived sGC co-expression modules correlate with human DKD outcome

Next, we sought to understand cell-type-specific changes in gene groups. To this end, we used WGCNA to identify modules correlating with sGC expression. We first created a WGCNA-compatible metanuclei dataset (Figure 7A and STAR Methods), from which we retrieved seven gene modules (Figures 7B and S13A; Data S19). Some of these modules showed high kME values for sGC genes (Figures 7C and S13B), indicating sGC genes as important hub genes for their corresponding co-expression modules. We noticed overall high specificity of modules for cell clusters along the PT-Mesench trajectory (Figures 7D, S13C, and S13D). For example, the turquoise module was enriched in healthy PST. Blue, yellow, and black modules were enriched in ProfibPT. Green and red modules were enriched in Mesench. Module-specific phenotypes were consistent with Kyoto Encyclopedia of Genes and Genomes (KEGG) pathway and GO term analysis, suggesting pathway enrichment representing healthy PT function in the turquoise model, profibrotic processes in blue, yellow, and black models, dedifferentiation to non-proximal tubule in the brown model, and ECM/Mesench processes in green and red modules, respectively (Figure S14A and Data S20). Based on cell-type-specific sGC expression, we created a composite sGC co-expression WGCNA score of those gene modules demonstrating the highest co-expression with sGC in non-healthy PT and stromal cells along the trajectory (Figure S14B). This WGCNA score showed highest enrichment in ProfibPT and Mesench (Figure 7E). We were reassured that ProfibPT and Mesench clusters had the highest overlap of this composite WGCNA score with cluster-specific DEGs (Figure 7F), suggesting that these two cell identities were most associated with sGC gene expression in non-healthy injury states.

Finally, we wanted to understand the relevance of sGC-associated changes to patient samples. We asked whether we could leverage this unbiased orthogonal dataset to a group with 991 microdissected kidney tubule RNA-seq samples from human individuals with and without DKD to infer disease-relevant parameters (Figure 7G and Data S21). Indeed, WGCNA scores were significantly higher in individuals with high albuminuria (Figure 7H), low glomerular filtration rate (GFR) (Figure 7I), and high degrees of fibrosis on histopathological examination (Figure 7J). Furthermore, unsupervised clustering analysis of 991 patient samples was able to clearly group patients into two clusters based on sGC co-expression-derived WGCNA score (Figure 7K). Reassuringly, although these two groups were matched with respect to clinical characteristics such as age, gender, race, blood pressure, and—most importantly—prevalence of diabetes, samples with high WGCNA score had significantly higher prevalence of DKD, albuminuria, fibrosis, and glomerulosclerosis than samples with low WGCNA score, as well as significantly lower GFR (Figure 7L). These results suggest that the WGCNA sGC co-expression score was able to stratify subjects by clinical disease-relevant parameters. Accordingly, principal component analysis plots stratifying human samples into control, early DKD, and advanced DKD largely overlapped with WGCNA scoring (Figures S14C and S14D). We also validated these results in an independent human kidney bulk RNA-seq dataset including individuals with early and advanced DKD[36] (Figure 7M).

Next, we built several multiple regression models to estimate the relative contribution of WGCNA score to disease-relevant parameters. Multiple linear regression models demonstrated that the WGCNA score estimated fibrosis ($\beta = 1.144$, $p < 0.001$), glomerulosclerosis ($\beta = 0.616$, $p < 0.001$), and GFR ($\beta = -0.546$, $p < 0.001$) independent of other clinical variables (Data S22). Ordinal logistic regression showed the WGCNA score to independently estimate albuminuria (odds ratio = 1.045, $p < 0.001$) (Data S22), such that a high WGCNA score was associated with albuminuria (Figure S14E).

Having established the sGC co-expression WGCNA score as a valuable tool for assessing kidney outcomes relevant to DKD, we finally asked how pharmacological sGC modulation in the ZSF1 rat model would influence WGCNA score. Indeed, we observed lower WGCNA scores for rats treated with sGCact compared with untreated obese diabetic rats, while sGCstim had little effect (Figure 7N), and we observed a negative correlation between WGCNA score and cGMP effects (Figure S14F), suggesting that WGCNA score was a useful measure for estimating treatment effect size following sGC modulation. Our observations might also partially explain the improved kidney functional and structural outcome seen following sGCact treatment when compared with sGCstim.

Figure 6. Gene-regulatory network analysis highlights cell-type-specific transcription factors driving the PT-to-mesenchymal trajectory and prioritizes cell types of action for sGC modulation

(A) Cell clusters from the PT-Mesench trajectory were subjected to gene-regulatory network (GRN) analysis.

(B) Regulon density as a surrogate for stability of regulon states along the trajectory in diffusion map space.

(C) Heatmap of cell-type-specific binarized regulon activity. Rows represent regulons of transcription factors (TFs) and their predicted targets, columns represent cells along the trajectory, colored by cell clusters as in (A). Top specific TFs per cluster are annotated.

(D) Binarized regulon activity for top cluster-specific TFs along the trajectory in diffusion map space.

(E) The GRN dataset was filtered for TFs predicted to target sGC genes (*Gucy1a2*, *Gucy1b2*).

(F) Heatmap visualizing specificity of regulons (rows) for cell clusters along the trajectory (columns). Color denotes regulon specificity score (RSS). Regulons are color-annotated for normalized enrichment score (NES), number of motifs, and their predicted sGC target gene (*Gucy1a2* or *Gucy1b2*). Top cell-cluster-specific regulons are annotated.

(G) Expression dot plot for sGC genes and top cell-cluster-specific TFs from (F). Dot size denotes percentage of cells expressing the marker. Color scale represents average gene-expression values.

(H) Feature plots for sGC genes (*Gucy1a1*, *Gucy1a2*, *Gucy1b1*, *Gucy1b2*) along the trajectory in diffusion map space.

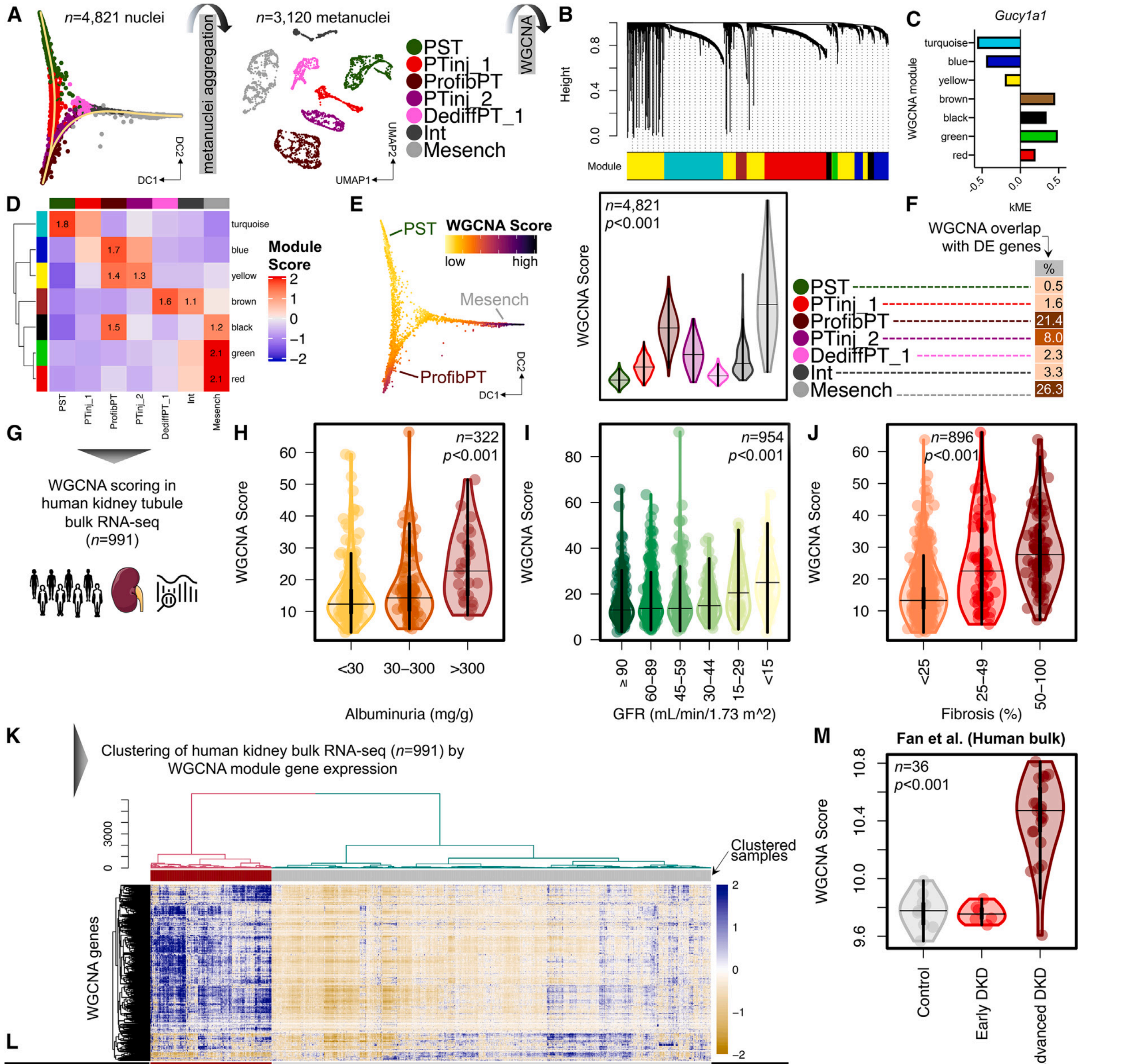

L

Variables	n=214	n=777	p
Age (yrs)	61.4 ± 14.0	61.7 ± 12.6	0.917
Male (%)	62.2	61.0	0.727
Afric. Am. (%)	22.9	18.3	0.156
HTN (%)	73.2	68.1	0.175
SBP (mmHg)	138 ± 22	136 ± 20	0.576
DBP (mmHg)	76 ± 13	77 ± 12	0.182
T1D/T2D (%)	6.2/34.6	5.1/27.9	0.106
**DKD (%)**	68.7	23.9	**<0.001**
**uACR >300 (%)**	21.5	4.7	**<0.001**
**eGFR (mL/min/1.73m²)**	54.9 ± 31.2	69.3 ± 24.0	**<0.001**
**Fibrosis (%)**	33.5 ± 34.5	8.7 ± 13.7	**<0.001**
**Glomeruloscl. (%)**	24.9 ± 31.5	10.2 ± 13.2	**<0.001**

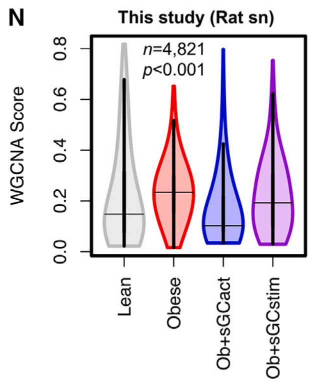

(legend on next page)

DISCUSSION

Here we present the first comprehensive single-cell resolution atlas of DKD in the ZSF1 rat model. Not only does the ZSF1 rat recapitulate human DKD phenotypically, it also exhibits excellent correlation of cell-type-specific transcriptomes with that of human DKD,[30,31] underscoring the value of the ZSF1 rat for human DKD translational and pharmacological studies. To the best of our knowledge, we are the first to present a single-cell resolution head-to-head comparison of a sGCstim and sGCact treatment. In our model, we find superiority of sGCact over sGCstim in attenuating functional and structural DKD. We highlight key cell types; podocytes, PT, and mesenchymal cells showed the largest changes in the single-cell data, coinciding with the expression of sGC pathway genes. We demonstrate that sGC co-expression gene modules can be successfully used to stratify patient kidney samples by DKD renal outcome measures such as GFR, albuminuria, glomerulosclerosis, and interstitial fibrosis, indicating the relevance of the sGC pathway to patients.

Animal models play a key role in human disease understanding. While recently gene and pathway discovery approaches have heavily focused on patient samples, animal models remain critical for pharmacological gene and pathway modulation and proof-of-concept studies. Here we provide a comprehensive phenotypic, histological, biochemical, and single-cell gene-expression description of the ZSF1 rat model. Comparison of rat samples with human DKD shows very strong similarities but also differences, indicating that the model is useful to analyze specific disease manifestations. Detailed single-cell and omics analysis of animal models will be critical for therapeutics discovery. We present our data for our users via an easy-to-use interface at http://www.susztaklab.com/ZSF1_sGC_snRNA/.

Furthermore, we present here an important tool for examining therapeutic effectiveness, target cell types, and mechanism of action via single-cell sequencing. We have been lacking a detailed understanding of individual sGC modulation effects on a cellular level, despite consistent kidney phenotypic improvement by sGC agonists in preclinical DKD models.[8,15–18] Single-cell transcriptomics with an unbiased tensor decomposition approach highlighted PT and stromal cells as key target cell types of sGC-cGMP-mediated effects. This is mostly consistent with the cell-type expression of sGC pathway genes. Furthermore, we robustly demonstrate the close transcriptomic relationship between PT and stromal cells: During diabetic injury, formerly healthy PT cells transition via several cell states (PTinj, ProfibPT) toward a Mesench phenotype. Numerous studies have implicated EMT in renal fibrosis;[41–44] however, a potential connection to NO-sGC-cGMP signaling has not been described so far and mechanistic animal studies will be needed for future validation. In summary, while multiple cell types show changes in disease state and following drug treatment, novel single-cell tools are still able to identify key disease-driving cell types.

Furthermore, we identified important differences between sGCstim and sGCact. Studies have established the role of reactive oxygen species production, oxidative stress coupled with compromised NO bioactivity, and endothelial dysfunction.[45–47] To this effect, it is important to note that sGCstim and sGCact differ in their ability to generate cGMP under pathophysiological conditions such as the high oxidative stress state in DKD.[9] While sGCstim depend on a reduced iron (Fe^{2+}) state of sGC, sGCact preferentially target sGC at the heme-free or oxidized, NO-unresponsive sGC enzyme, explaining their higher pharmacological activity under conditions of high oxidative stress and NO depletion.[14] Our ZSF1 rat model results corroborated this hypothesis, demonstrating stronger attenuation of the DKD phenotype, such as increased kidney function, reduced kidney injury markers, glomerulosclerosis, proteinuria, and interstitial fibrosis, upon sGCact treatment compared with sGCstim. Moreover, we confirmed (in kidneys from both diabetic ZSF1 rats and human subjects with advanced DKD) that lower cGMP effects, a proxy of decreased downstream sGC action, correlated positively with the inability to negatively regulate oxidative stress. Most importantly, cGMP effects measured on a transcriptomic level were restored to a larger extent by sGCact than sGCstim. These results indicate that single-cell gene-expression analysis is able

Figure 7. WGCNA-derived sGC co-expression modules correlate with human DKD outcome

(A) Metanuclei aggregation of cell clusters from the PT-Mesench trajectory as a prerequisite for performing weighted gene correlation network analysis (WGCNA).

(B) Hierarchical cluster tree showing gene co-expression modules identified by WGCNA in cells along the PT-Mesench trajectory revealed seven modules (color-coded).

(C) Intramodular connectivity (kME) values show *Gucy1a1* as a hub gene for brown, black, green, and red modules.

(D) Heatmap demonstrating high specificity of WGCNA modules (rows) for cell clusters along the trajectory (columns).

(E) Composite sGC co-expression WGCNA score along the trajectory in diffusion map space (left) and per cell cluster (right). p value is given for one-way ANOVA (Tukey corrected).

(F) Percentage overlap of composite sGC co-expression WGCNA genes with cluster-specific DEGs.

(G) The composite sGC co-expression WGCNA module gene set was used to score 991 bulk microdissected kidney tubule RNA-seq samples from human individuals with and without DKD. WGCNA scores were then correlated with clinical and histopathology outcome variables.

(H–J) WGCNA score in human kidney tubules by degree of albuminuria (H), glomerular filtration rate (GFR) (I), and percentage kidney fibrosis (J). p values are given for one-way ANOVA (Tukey corrected).

(K) Dendrogram (top) representing hierarchical clustering of 991 human kidney tubule samples (columns) based on their expression of composite WGCNA module genes (rows) displayed in the corresponding heatmap (below).

(L) Clinical and kidney functional and structural outcome characteristics of patients clustered by composite WGCNA module gene expression in (K). p values are given for either Student's t, Wilcoxon-Mann-Whitney (for continuous variables), or Fisher's exact test (for categorical variables). HTN, hypertension; SBP, systolic blood pressure; DBP, diastolic blood pressure; T1D/T2D, type 1/type2 diabetes; DKD, diabetic kidney disease; uACR, urinary albumin-to-creatinine ratio; eGFR, estimated glomerular filtration rate.

(M and N) WGCNA score in human kidney bulk RNA-seq samples by degree of disease severity (M) as well as in ZSF1 rat kidney cells from the PT-Mesench trajectory by treatment group (N). p values are given for one-way ANOVA (Tukey corrected).

to identify not only disease-driving cell types but also disease-critical pathways and drug mechanisms of action.

In summary, we present the first single-cell resolution atlas for the ZSF1 rat DKD model and a head-to-head comparison of sGCact and sGCstim effects in DKD. Our single-cell analysis was able to highlight key disease-driving cell types (podocyte, PT, and stromal cells) and disease-driving mechanisms. Finally, we show the potential relevance of animal model observations to patient samples and show that sGC co-expression can be used to stratify human DKD kidney samples by parameters relevant for kidney functional and structural outcomes.

Limitations of the study

Our study has some limitations. We provide consistent data demonstrating higher efficacy of sGCact compared with sGCstim on several readouts, but we lack data on dose-response relationships of sGCact and sGCstim, which might be important for fine-tuning of the efficacy parameters. To avoid potential bias by blood pressure reduction through sGCact, we used doses that had no or minimal effects on blood pressure, while kidney-protective effects of sGCstim in ZSF1 rats are known to require higher dosages that are active on blood pressure. Future studies should address this limitation by comparing sGCact and sGCstim with a wider dose range and a more extensive set of physiological readouts. Additionally, the renoprotective effects in our ZSF1 model may be partially attributed to improvements in glycemia, but it is difficult to estimate the extent of this contribution.[48] Furthermore, given the specific expression of sGC subunit mRNAs in stromal and PT cells, it is difficult to distinguish direct sGC agonism in specific kidney cells from indirect effects such as changes in renal hemodynamics and glucose/lipid metabolism, which warrant further mechanistic studies.

STAR★METHODS

Detailed methods are provided in the online version of this paper and include the following:

- KEY RESOURCES TABLE
- RESOURCE AVAILABILITY
  - Lead contact
  - Materials availability
  - Data and code availability
- EXPERIMENTAL MODEL AND SUBJECT DETAILS
  - ZSF1 rat model
  - Human sample procurement
- METHOD DETAILS
  - ZSF1 rat model
  - Preparation of rat single-nuclei suspension
  - Rat single-nuclei RNA-seq
  - Human kidney microdissection and bulk RNA-sequencing
- QUANTIFICATION AND STATISTICAL ANALYSIS
- ADDITIONAL RESOURCES

SUPPLEMENTAL INFORMATION

Supplemental information can be found online at https://doi.org/10.1016/j.xcrm.2023.100992.

ACKNOWLEDGMENTS

This work was supported by Bayer AG. However, the funders had no direct involvement in the experiments, analysis, and data presentation. Work in the Susztak lab is supported by NIH grants DK076077, DK087635, DK105821, and DK132630. M.S.B. is supported by grants from the German Research Foundation (Deutsche Forschungsgemeinschaft, DFG, BA 6205/2-1), Else Kroner-Fresenius Foundation, and the Berlin Institute of Health at Charité – Universitätsmedizin Berlin Clinician Scientist Program. We thank the University of Pennsylvania Diabetes Research Center (DRC) for the use of the next-generation sequencing core (P30-DK19525).

AUTHOR CONTRIBUTIONS

M.S.B., K. Susztak, M.P., and M.G. designed and conceived the study. A.F., J.V., I.M., K. Siudak, M.G., and M.P. conducted animal studies and histopathological, biochemical, and proteomics analyses. F.E. and P.S. oversaw animal studies. M.S.B. and J.F. conducted single-cell RNA-seq experiments; A.A. conducted bulk RNA-seq experiments. M.S.B. conducted bioinformatics analysis. All authors discussed and commented on the results. M.S.B. and K. Susztak wrote the manuscript, and all authors edited and approved of the final manuscript.

DECLARATION OF INTERESTS

A.F., J.V., I.M., K. Siudak, F.E., P.S., M.G., and M.P. are employees of Bayer AG. M.S.B. reports consultancy: Boehringer Ingelheim; editorial board membership: *Journal of the American Society of Nephrology*. K. Susztak reports research support: AstraZeneca, Bayer, Boehringer Ingelheim, Calico, Genentech, Gilead, GSK, Jnana, Lilly, Maze, Merck, Novartis, Novo Nordisk, Regeneron, Variant Bio, and Ventus; advisory board membership: Jnana Therapeutics and Pfizer; consultancy: AstraZeneca, Bayer, GSK, Jnana Therapeutics, Maze, Novo Nordisk, Pfizer, and Ventus; patents: Jag1- and Notch-based targeting of chronic kidney disease; editorial board membership: *Cell Metabolism*, *eBioMedicine*, *Journal of the American Society of Nephrology*, *Journal of Clinical Investigation*, *Kidney International*, and *Med*.

Received: October 17, 2022
Revised: January 29, 2023
Accepted: March 14, 2023
Published: April 5, 2023

REFERENCES

1. Jager, K.J., Kovesdy, C., Langham, R., Rosenberg, M., Jha, V., and Zoccali, C. (2019). A single number for advocacy and communication-worldwide more than 850 million individuals have kidney diseases. Kidney Int. *96*, 1048–1050. https://doi.org/10.1016/j.kint.2019.07.012.
2. U.S. Renal Data System (2014). USRDS 2014 Annual Data Report: Atlas of End-Stage Renal Disease in the United States, National Institutes of Health (Bethesda, MD: National Institute of Diabetes and Digestive and Kidney Diseases).
3. Naylor, K.L., Kim, S.J., McArthur, E., Garg, A.X., McCallum, M.K., and Knoll, G.A. (2019). Mortality in incident maintenance dialysis patients versus incident solid organ cancer patients: a population-based cohort. Am. J. Kidney Dis. *73*, 765–776. https://doi.org/10.1053/j.ajkd.2018.12.011.
4. Tervaert, T.W.C., Mooyaart, A.L., Amann, K., Cohen, A.H., Cook, H.T., Drachenberg, C.B., Ferrario, F., Fogo, A.B., Haas, M., de Heer, E., et al. (2010). Pathologic classification of diabetic nephropathy. J. Am. Soc. Nephrol. *21*, 556–563. https://doi.org/10.1681/asn.2010010010.

CellPress
OPEN ACCESS

5. Singh, D.K., Winocour, P., and Farrington, K. (2011). Oxidative stress in early diabetic nephropathy: fueling the fire. Nat. Rev. Endocrinol. *7*, 176–184. https://doi.org/10.1038/nrendo.2010.212.

6. Cheng, H., and Harris, R.C. (2014). Renal endothelial dysfunction in diabetic nephropathy. Cardiovasc. Hematol. Disord.: Drug Targets *14*, 22–33. https://doi.org/10.2174/1871529x14666140401110841.

7. Krishnan, S.M., Kraehling, J.R., Eitner, F., Bénardeau, A., and Sandner, P. (2018). The impact of the nitric oxide (NO)/Soluble guanylyl cyclase (sGC) signaling cascade on kidney Health and disease: a preclinical perspective. Int. J. Mol. Sci. *19*, 1712. https://doi.org/10.3390/ijms19061712.

8. Stasch, J.-P., Schlossmann, J., and Hocher, B. (2015). Renal effects of soluble guanylate cyclase stimulators and activators: a review of the preclinical evidence. Curr. Opin. Pharmacol. *21*, 95–104. https://doi.org/10.1016/j.coph.2014.12.014.

9. Sandner, P., Zimmer, D.P., Milne, G.T., Follmann, M., Hobbs, A., and Stasch, J.P. (2021). Soluble guanylate cyclase stimulators and activators. Handb. Exp. Pharmacol. *264*, 355–394. https://doi.org/10.1007/164_2018_197.

10. Stasch, J.-P., and Hobbs, A.J. (2009). NO-independent, haem-dependent soluble guanylate cyclase stimulators. In cGMP: Generators, Effectors and Therapeutic Implications, H.H.H.W. Schmidt, F. Hofmann, and J.-P. Stasch, eds. (Springer Berlin Heidelberg), pp. 277–308. https://doi.org/10.1007/978-3-540-68964-5_13.

11. Pan, J., Zhang, X., Yuan, H., Xu, Q., Zhang, H., Zhou, Y., Huang, Z.-X., and Tan, X. (2016). The molecular mechanism of heme loss from oxidized soluble guanylate cyclase induced by conformational change. Biochim. Biophys. Acta *1864*, 488–500. https://doi.org/10.1016/j.bbapap.2016.02.012.

12. Sharina, I.G., and Martin, E. (2017). The role of reactive oxygen and nitrogen species in the expression and splicing of nitric oxide receptor. Antioxidants Redox Signal. *26*, 122–136. https://doi.org/10.1089/ars.2016.6687.

13. Schmidt, H.H.H.W., Schmidt, P.M., and Stasch, J.P. (2009). NO- and haem-independent soluble guanylate cyclase activators. Handb. Exp. Pharmacol. *309*, 309–339. https://doi.org/10.1007/978-3-540-68964-5_14.

14. Thoonen, R., Cauwels, A., Decaluwe, K., Geschka, S., Tainsh, R.E., Delanghe, J., Hochepied, T., De Cauwer, L., Rogge, E., Voet, S., et al. (2015). Cardiovascular and pharmacological implications of haem-deficient NO-unresponsive soluble guanylate cyclase knock-in mice. Nat. Commun. *6*, 8482. https://doi.org/10.1038/ncomms9482.

15. Boustany-Kari, C.M., Harrison, P.C., Chen, H., Lincoln, K.A., Qian, H.S., Clifford, H., Wang, H., Zhang, X., Gueneva-Boucheva, K., Bosanac, T., et al. (2016). A soluble guanylate cyclase activator inhibits the progression of diabetic nephropathy in the ZSF1 rat. J. Pharmacol. Exp. Therapeut. *356*, 712–719. https://doi.org/10.1124/jpet.115.230706.

16. Liu, G., Shea, C.M., Jones, J.E., Price, G.M., Warren, W., Lonie, E., Yan, S., Currie, M.G., Profy, A.T., Masferrer, J.L., and Zimmer, D.P. (2020). Praliciguat inhibits progression of diabetic nephropathy in ZSF1 rats and suppresses inflammation and apoptosis in human renal proximal tubular cells. Am. J. Physiol. Ren. Physiol. *319*, F697–F711. https://doi.org/10.1152/ajprenal.00003.2020.

17. Bénardeau, A., Kahnert, A., Schomber, T., Meyer, J., Pavkovic, M., Kretschmer, A., Lawrenz, B., Hartmann, E., Mathar, I., Hueser, J., et al. (2021). Runcaciguat, a novel soluble guanylate cyclase activator, shows renoprotection in hypertensive, diabetic, and metabolic preclinical models of chronic kidney disease. Naunyn-Schmiedeberg's Arch. Pharmacol. *394*, 2363–2379. https://doi.org/10.1007/s00210-021-02149-4.

18. Hu, L., Chen, Y., Zhou, X., Hoek, M., Cox, J., Lin, K., Liu, Y., Blumenschein, W., Grein, J., and Swaminath, G. (2022). Effects of soluble guanylate cyclase stimulator on renal function in ZSF-1 model of diabetic nephropathy. PLoS One *17*, e0261000. https://doi.org/10.1371/journal.pone.0261000.

19. Liu, G., Shea, C.M., Jones, J.E., Price, G.M., Warren, W., Lonie, E., Yan, S., Currie, M.G., Profy, A.T., Masferrer, J.L., and Zimmer, D.P. (2020). Praliciguat inhibits progression of diabetic nephropathy in ZSF1 rats and suppresses inflammation and apoptosis in human renal proximal tubular cells. Am. J. Physiol. Ren. Physiol. *319*, F697–F711. https://doi.org/10.1152/ajprenal.00003.2020.

20. Bilan, V.P., Salah, E.M., Bastacky, S., Jones, H.B., Mayers, R.M., Zinker, B., Poucher, S.M., and Tofovic, S.P. (2011). Diabetic nephropathy and long-term treatment effects of rosiglitazone and enalapril in obese ZSF1 rats. J. Endocrinol. *210*, 293–308. https://doi.org/10.1530/JOE-11-0122.

21. Tofovic, S.P., Salah, E.M., Jackson, E.K., and Melhem, M. (2007). Early renal injury induced by caffeine consumption in obese, diabetic ZSF1 rats. Renal Fail. *29*, 891–902. https://doi.org/10.1080/08860220701569846.

22. Prabhakar, S., Starnes, J., Shi, S., Lonis, B., and Tran, R. (2007). Diabetic nephropathy is associated with oxidative stress and decreased renal nitric oxide production. J. Am. Soc. Nephrol. *18*, 2945–2952. https://doi.org/10.1681/ASN.2006080895.

23. Isomaa, B., Almgren, P., Tuomi, T., Forsén, B., Lahti, K., Nissén, M., Taskinen, M.R., and Groop, L. (2001). Cardiovascular morbidity and mortality associated with the metabolic syndrome. Diabetes Care *24*, 683–689. https://doi.org/10.2337/diacare.24.4.683.

24. Sperling, L.S., Mechanick, J.I., Neeland, I.J., Herrick, C.J., Després, J.P., Ndumele, C.E., Vijayaraghavan, K., Handelsman, Y., Puckrein, G.A., Araneta, M.R.G., et al. (2015). The CardioMetabolic Health alliance: working toward a new care model for the metabolic syndrome. J. Am. Coll. Cardiol. *66*, 1050–1067. https://doi.org/10.1016/j.jacc.2015.06.1328.

25. Han, W.K., Bailly, V., Abichandani, R., Thadhani, R., and Bonventre, J.V. (2002). Kidney Injury Molecule-1 (KIM-1): a novel biomarker for human renal proximal tubule injury. Kidney Int. *62*, 237–244. https://doi.org/10.1046/j.1523-1755.2002.00433.x.

26. Bolignano, D., Donato, V., Coppolino, G., Campo, S., Buemi, A., Lacquaniti, A., and Buemi, M. (2008). Neutrophil gelatinase–associated lipocalin (NGAL) as a marker of kidney damage. Am. J. Kidney Dis. *52*, 595–605. https://doi.org/10.1053/j.ajkd.2008.01.020.

27. Kobayashi, H., Looker, H.C., Satake, E., Saulnier, P.J., Md Dom, Z.I., O'Neil, K., Ihara, K., Krolewski, B., Galecki, A.T., Niewczas, M.A., et al. (2022). Results of untargeted analysis using the SOMAscan proteomics platform indicates novel associations of circulating proteins with risk of progression to kidney failure in diabetes. Kidney Int. *102*, 370–381. https://doi.org/10.1016/j.kint.2022.04.022.

28. Tofovic, S.P., Kusaka, H., Kost, C.K., Jr., and Bastacky, S. (2000). Renal function and structure in diabetic, hypertensive, obese ZDFxSHHF-hybrid rats. Ren. Fail. *22*, 387–406. https://doi.org/10.1081/jdi-100100882.

29. Rafikova, O., Salah, E.M., and Tofovic, S.P. (2008). Renal and metabolic effects of tempol in obese ZSF1 rats–distinct role for superoxide and hydrogen peroxide in diabetic renal injury. Metabolism *57*, 1434–1444. https://doi.org/10.1016/j.metabol.2008.05.014.

30. Wilson, P.C., Wu, H., Kirita, Y., Uchimura, K., Ledru, N., Rennke, H.G., Welling, P.A., Waikar, S.S., and Humphreys, B.D. (2019). The single-cell transcriptomic landscape of early human diabetic nephropathy. Proc. Natl. Acad. Sci. USA *116*, 19619–19625. https://doi.org/10.1073/pnas.1908706116.

31. Lake, B.B., Menon, R., Winfree, S., Hu, Q., Ferreira, R.M., Kalhor, K., Barwinska, D., Otto, E.A., Ferkowicz, M., Diep, D., et al. (2021). An atlas of healthy and injured cell states and niches in the human kidney. Preprint at bioRxiv. https://doi.org/10.1101/2021.07.28.454201.

32. Wu, J., Sun, Z., Yang, S., Fu, J., Fan, Y., Wang, N., Hu, J., Ma, L., Peng, C., Wang, Z., et al. (2022). Kidney single-cell transcriptome profile reveals distinct response of proximal tubule cells to SGLT2i and ARB treatment in diabetic mice. Mol. Ther. *30*, 1741–1753. https://doi.org/10.1016/j.ymthe.2021.10.013.

33. Balzer, M.S., Doke, T., Yang, Y.-W., Aldridge, D.L., Hu, H., Mai, H., Mukhi, D., Ma, Z., Shrestha, R., Palmer, M.B., et al. (2022). Single-cell analysis highlights differences in druggable pathways underlying adaptive or fibrotic kidney regeneration. Nat. Commun. *13*, 4018. https://doi.org/10.1038/s41467-022-31772-9.

34. Doke, T., Abedini, A., Aldridge, D.L., Yang, Y.W., Park, J., Hernandez, C.M., Balzer, M.S., Shrestra, R., Coppock, G., Rico, J.M.I., et al. (2022). Single-cell analysis identifies the interaction of altered renal tubules with basophils orchestrating kidney fibrosis. Nat. Immunol. *23*, 947–959. https://doi.org/10.1038/s41590-022-01200-7.

35. Kirita, Y., Wu, H., Uchimura, K., Wilson, P.C., and Humphreys, B.D. (2020). Cell profiling of mouse acute kidney injury reveals conserved cellular responses to injury. Proc. Natl. Acad. Sci. USA *117*, 15874–15883. https://doi.org/10.1073/pnas.2005477117.

36. Fan, Y., Yi, Z., D'Agati, V.D., Sun, Z., Zhong, F., Zhang, W., Wen, J., Zhou, T., Li, Z., He, L., et al. (2019). Comparison of kidney transcriptomic profiles of early and advanced diabetic nephropathy reveals potential new mechanisms for disease progression. Diabetes *68*, 2301–2314. https://doi.org/10.2337/db19-0204.

37. Theilig, F., Bostanjoglo, M., Pavenstädt, H., Grupp, C., Holland, G., Slosarek, I., Gressner, A.M., Russwurm, M., Koesling, D., and Bachmann, S. (2001). Cellular distribution and function of soluble guanylyl cyclase in rat kidney and liver. J. Am. Soc. Nephrol. *12*, 2209–2220. https://doi.org/10.1681/asn.V12112209.

38. Liu, H., Doke, T., Guo, D., Sheng, X., Ma, Z., Park, J., Vy, H.M.T., Nadkarni, G.N., Abedini, A., Miao, Z., et al. (2022). Epigenomic and transcriptomic analyses define core cell types, genes and targetable mechanisms for kidney disease. Nat. Genet. *54*, 950–962. https://doi.org/10.1038/s41588-022-01097-w.

39. Craig, D.W., Millis, M.P., and DiStefano, J.K. (2009). Genome-wide SNP genotyping study using pooled DNA to identify candidate markers mediating susceptibility to end-stage renal disease attributed to Type 1 diabetes. Diabet. Med. *26*, 1090–1098. https://doi.org/10.1111/j.1464-5491.2009.02846.x.

40. Matsuba, R., Sakai, K., Imamura, M., Tanaka, Y., Iwata, M., Hirose, H., Kaku, K., Maegawa, H., Watada, H., Tobe, K., et al. (2015). Replication study in a Japanese population to evaluate the association between 10 SNP loci, identified in European genome-wide association studies, and type 2 diabetes. PLoS One *10*, e0126363. https://doi.org/10.1371/journal.pone.0126363.

41. Grande, M.T., Sánchez-Laorden, B., López-Blau, C., De Frutos, C.A., Boutet, A., Arévalo, M., Rowe, R.G., Weiss, S.J., López-Novoa, J.M., and Nieto, M.A. (2015). Snail1-induced partial epithelial-to-mesenchymal transition drives renal fibrosis in mice and can be targeted to reverse established disease. Nat. Med. *21*, 989–997. https://doi.org/10.1038/nm.3901.

42. Carew, R.M., Wang, B., and Kantharidis, P. (2012). The role of EMT in renal fibrosis. Cell Tissue Res. *347*, 103–116. https://doi.org/10.1007/s00441-011-1227-1.

43. Lovisa, S., LeBleu, V.S., Tampe, B., Sugimoto, H., Vadnagara, K., Carstens, J.L., Wu, C.C., Hagos, Y., Burckhardt, B.C., Pentcheva-Hoang, T., et al. (2015). Epithelial-to-mesenchymal transition induces cell cycle arrest and parenchymal damage in renal fibrosis. Nat. Med. *21*, 998–1009. https://doi.org/10.1038/nm.3902.

44. LeBleu, V.S., Taduri, G., O'Connell, J., Teng, Y., Cooke, V.G., Woda, C., Sugimoto, H., and Kalluri, R. (2013). Origin and function of myofibroblasts in kidney fibrosis. Nat. Med. *19*, 1047–1053. https://doi.org/10.1038/nm.3218.

45. Lundberg, J.O., Gladwin, M.T., and Weitzberg, E. (2015). Strategies to increase nitric oxide signalling in cardiovascular disease. Nat. Rev. Drug Discov. *14*, 623–641. https://doi.org/10.1038/nrd4623.

46. Schiffer, T.A., Lundberg, J.O., Weitzberg, E., and Carlström, M. (2020). Modulation of mitochondria and NADPH oxidase function by the nitrate-nitrite-NO pathway in metabolic disease with focus on type 2 diabetes. Biochim. Biophys. Acta, Mol. Basis Dis. *1866*, 165811. https://doi.org/10.1016/j.bbadis.2020.165811.

47. Carlstrom, M., and Montenegro, M.F. (2019). Therapeutic value of stimulating the nitrate-nitrite-nitric oxide pathway to attenuate oxidative stress and restore nitric oxide bioavailability in cardiorenal disease. J. Intern. Med. *285*, 2–18. https://doi.org/10.1111/joim.12818.

48. Hanrahan, J.P., de Boer, I.H., Bakris, G.L., Wilson, P.J., Wakefield, J.D., Seferovic, J.P., Chickering, J.G., Chien, Y.T., Carlson, K., Cressman, M.D., et al. (2020). Effects of the soluble guanylate cyclase stimulator praliciguat in diabetic kidney disease: a randomized placebo-controlled clinical trial. Clin. J. Am. Soc. Nephrol. *16*, 59–69. https://doi.org/10.2215/CJN.08410520.

49. Balzer, M.S., and Susztak, K. (2022). Suppl. Data for Defining the effects of soluble guanylate cyclase modulation in ZSF1 rat diabetic kidney disease at single-cell resolution. Preprint at Zenodo, 10.5281/zenodo.6950236. https://doi.org/10.5281/zenodo.6950236.

50. Balzer, M.S., and Susztak, K. (2022). Codes for Defining the effects of soluble guanylate cyclase modulation in ZSF1 rat diabetic kidney disease at single-cell resolution. Preprint at Zenodo, 10.5281/zenodo.6959731. https://doi.org/10.5281/zenodo.6959731.

51. Assarsson, E., Lundberg, M., Holmquist, G., Björkesten, J., Thorsen, S.B., Ekman, D., Eriksson, A., Rennel Dickens, E., Ohlsson, S., Edfeldt, G., et al. (2014). Homogenous 96-plex PEA immunoassay exhibiting high sensitivity, specificity, and excellent scalability. PLoS One *9*, e95192. https://doi.org/10.1371/journal.pone.0095192.

52. McGinnis, C.S., Murrow, L.M., and Gartner, Z.J. (2019). DoubletFinder: doublet detection in single-cell RNA sequencing data using artificial nearest neighbors. Cell Syst. *8*, 329–337.e4. https://doi.org/10.1016/j.cels.2019.03.003.

53. Korsunsky, I., Millard, N., Fan, J., Slowikowski, K., Zhang, F., Wei, K., Baglaenko, Y., Brenner, M., Loh, P.R., and Raychaudhuri, S. (2019). Fast, sensitive and accurate integration of single-cell data with Harmony. Nat. Methods *16*, 1289–1296. https://doi.org/10.1038/s41592-019-0619-0.

54. Park, J., Shrestha, R., Qiu, C., Kondo, A., Huang, S., Werth, M., Li, M., Barasch, J., and Suszták, K. (2018). Single-cell transcriptomics of the mouse kidney reveals potential cellular targets of kidney disease. Science *360*, 758–763. https://doi.org/10.1126/science.aar2131.

55. Abedini, A., Zhu, Y.O., Chatterjee, S., Halasz, G., Devalaraja-Narashimha, K., Shrestha, R., S Balzer, M., Park, J., Zhou, T., Ma, Z., et al. (2021). Urinary single-cell profiling captures the cellular diversity of the kidney. J. Am. Soc. Nephrol. *32*, 614–627. https://doi.org/10.1681/ASN.2020050757.

56. Dhillon, P., Park, J., Hurtado Del Pozo, C., Li, L., Doke, T., Huang, S., Zhao, J., Kang, H.M., Shrestra, R., Balzer, M.S., et al. (2021). The nuclear receptor ESRRA protects from kidney disease by coupling metabolism and differentiation. Cell Metabol. *33*, 379–394.e8. https://doi.org/10.1016/j.cmet.2020.11.011.

57. Miao, Z., Balzer, M.S., Ma, Z., Liu, H., Wu, J., Shrestha, R., Aranyi, T., Kwan, A., Kondo, A., Pontoglio, M., et al. (2021). Single cell regulatory landscape of the mouse kidney highlights cellular differentiation programs and disease targets. Nat. Commun. *12*, 2277. https://doi.org/10.1038/s41467-021-22266-1.

58. Balzer, M.S., Rohacs, T., and Susztak, K. (2022). How many cell types are in the kidney and what do they do? Annu. Rev. Physiol. *84*, 507–531. https://doi.org/10.1146/annurev-physiol-052521-121841.

59. Barry, D.M., McMillan, E.A., Kunar, B., Lis, R., Zhang, T., Lu, T., Daniel, E., Yokoyama, M., Gomez-Salinero, J.M., Sureshbabu, A., et al. (2019). Molecular determinants of nephron vascular specialization in the kidney. Nat. Commun. *10*, 5705. https://doi.org/10.1038/s41467-019-12872-5.

60. Chung, J.J., Goldstein, L., Chen, Y.J.J., Lee, J., Webster, J.D., Roose-Girma, M., Paudyal, S.C., Modrusan, Z., Dey, A., and Shaw, A.S. (2020). Single-cell transcriptome profiling of the kidney glomerulus identifies key cell types and reactions to injury. J. Am. Soc. Nephrol. *31*, 2341–2354. https://doi.org/10.1681/asn.2020020220.

61. Mitchel, J., Gordon, M.G., Perez, R.K., Biederstedt, E., Bueno, R., Ye, C.J., and Kharchenko, P.V. (2022). Tensor decomposition reveals coordinated multicellular patterns of transcriptional variation that distinguish and stratify disease individuals. Preprint at bioRxiv. https://doi.org/10.1101/2022.02.16.480703.

62. Wu, T., Hu, E., Xu, S., Chen, M., Guo, P., Dai, Z., Feng, T., Zhou, L., Tang, W., Zhan, L., et al. (2021). clusterProfiler 4.0: a universal enrichment tool for interpreting omics data. Innovation *2*, 100141. https://doi.org/10.1016/j.xinn.2021.100141.

63. Street, K., Risso, D., Fletcher, R.B., Das, D., Ngai, J., Yosef, N., Purdom, E., and Dudoit, S. (2018). Slingshot: cell lineage and pseudotime inference for single-cell transcriptomics. BMC Genom. *19*, 477. https://doi.org/10.1186/s12864-018-4772-0.

64. Angerer, P., Haghverdi, L., Büttner, M., Theis, F.J., Marr, C., and Buettner, F. (2016). destiny: diffusion maps for large-scale single-cell data in R. Bioinformatics *32*, 1241–1243. https://doi.org/10.1093/bioinformatics/btv715.

65. Trapnell, C., Cacchiarelli, D., Grimsby, J., Pokharel, P., Li, S., Morse, M., Lennon, N.J., Livak, K.J., Mikkelsen, T.S., and Rinn, J.L. (2014). The dynamics and regulators of cell fate decisions are revealed by pseudotemporal ordering of single cells. Nat. Biotechnol. *32*, 381–386. https://doi.org/10.1038/nbt.2859.

66. Cao, J., Spielmann, M., Qiu, X., Huang, X., Ibrahim, D.M., Hill, A.J., Zhang, F., Mundlos, S., Christiansen, L., Steemers, F.J., et al. (2019). The single-cell transcriptional landscape of mammalian organogenesis. Nature *566*, 496–502. https://doi.org/10.1038/s41586-019-0969-x.

67. Macosko, E.Z., Basu, A., Satija, R., Nemesh, J., Shekhar, K., Goldman, M., Tirosh, I., Bialas, A.R., Kamitaki, N., Martersteck, E.M., et al. (2015). Highly parallel genome-wide expression profiling of individual cells using nanoliter droplets. Cell *161*, 1202–1214. https://doi.org/10.1016/j.cell.2015.05.002.

68. Kuppe, C., Ibrahim, M.M., Kranz, J., Zhang, X., Ziegler, S., Perales-Patón, J., Jansen, J., Reimer, K.C., Smith, J.R., Dobie, R., et al. (2021). Decoding myofibroblast origins in human kidney fibrosis. Nature *589*, 281–286. https://doi.org/10.1038/s41586-020-2941-1.

69. Jin, S., Guerrero-Juarez, C.F., Zhang, L., Chang, I., Ramos, R., Kuan, C.-H., Myung, P., Plikus, M.V., and Nie, Q. (2021). Inference and analysis of cell-cell communication using CellChat. Nat. Commun. *12*, 1088. https://doi.org/10.1038/s41467-021-21246-9.

70. Aibar, S., González-Blas, C.B., Moerman, T., Huynh-Thu, V.A., Imrichova, H., Hulselmans, G., Rambow, F., Marine, J.C., Geurts, P., Aerts, J., et al. (2017). SCENIC: single-cell regulatory network inference and clustering. Nat. Methods *14*, 1083–1086. https://doi.org/10.1038/nmeth.4463.

71. Langfelder, P., and Horvath, S. (2008). WGCNA: an R package for weighted correlation network analysis. BMC Bioinf. *9*, 559. https://doi.org/10.1186/1471-2105-9-559.

72. Morabito, S., Miyoshi, E., Michael, N., Shahin, S., Martini, A.C., Head, E., Silva, J., Leavy, K., Perez-Rosendahl, M., and Swarup, V. (2021). Single-nucleus chromatin accessibility and transcriptomic characterization of Alzheimer's disease. Nat. Genet. *53*, 1143–1155. https://doi.org/10.1038/s41588-021-00894-z.

STAR★METHODS

KEY RESOURCES TABLE

REAGENT or RESOURCE	SOURCE	IDENTIFIER
Chemicals, peptides, and recombinant proteins		
Nonidet^{TM} P40 Substitute	Sigma	74385
Magnesium chloride	Sigma	M1028
Ultrapure BSA (50 mg/mL)	Thermo Fisher	AM2616
Protector RNase inhibitor	Sigma	3335399001
RNAlater	Ambion	AM7020
RNeasy RNA tissue lysis buffer	Qiagen	74106
Critical commercial assays		
Chromium Next GEM chip G Single Cell Kit	10X Genomics	PN-1000120
Chromium Next GEM Single Cell 3′ GEM Kit v3.1	10X Genomics	PN-1000121
Chromium Controller	10X Genomics	PN-120223
Chromium Single Index Kit T Set A	10X Genomics	PN-120262
RNeasy kit	Qiagen	74106
Bioanalyzer RNA 6000 Pico kit	Agilent Technologies	5067-1513
Bioanalyzer High Sensitivity DNA kit	Agilent Technologies	5067-4626
TruSeq RNA library prep kit v2	Illumina	RS-122-2001
Kidney Injury Panel 1 Rat Kit	Meso Scale Discovery	K15162C
Target 96 Mouse Exploratory Reagent Kit	Olink	95380
96.96 Integrated Fluid Circuit for Protein Expression	Olink	95007
Deposited data		
Human diabetic kidney disease bulk RNA-seq data	Fan et al.[36]	GSE128736; https://www.ncbi.nlm.nih.gov/geo/query/acc.cgi?acc=GSE128736
Human diabetic kidney disease snRNA-seq data	Lake et al.[31]	https://atlas.kpmp.org/repository/
Human diabetic kidney disease snRNA-seq data	Wilson et al.[30]	GSE131882; https://www.ncbi.nlm.nih.gov/geo/query/acc.cgi?acc=GSE131882
Software and algorithms		
CellChat v1.1.3	open source	https://github.com/sqjin/CellChat
Cell Ranger v5.0.1	10X Genomics	https://support.10xgenomics.com/single-cell-gene-expression/software/downloads/latest
cluster v2.1.0	open source	https://cran.r-project.org/web/packages/cluster/index.html
clusterProfiler v3.16.1	open source	https://guangchuangyu.github.io/software/clusterProfiler/
destiny v3.1.1	open source	https://github.com/theislab/destiny
DoubletFinder v2.0	open source	https://github.com/chris-mcginnis-ucsf/DoubletFinder
EnhancedVolcano v1.6.0	open source	https://github.com/kevinblighe/EnhancedVolcano
gam v1.20	open source	https://cran.r-project.org/web/packages/gam/index.html
genesorteR v0.4.3	open source	https://github.com/mahmoudibrahim/genesorteR
GOFigure v1.0.1	open source	https://gitlab.com/evogenlab/GO-Figure#installation
Harmony v0.1.0	open source	https://github.com/immunogenomics/harmony
MASS v7.3-51.6	open source	https://cran.r-project.org/web/packages/MASS/index.html
monocle2 v2.14.0	open source	http://cole-trapnell-lab.github.io/monocle-release/
monocle3 v0.1.3	open source	https://cole-trapnell-lab.github.io/monocle3/
RSEM v1.3.0	open source	https://github.com/deweylab/RSEM
rrvgo v1.0.2	open source	https://ssayols.github.io/rrvgo/
SCENIC v1.2.4	open source	https://aertslab.org/#scenic

(*Continued on next page*)

CellPress
OPEN ACCESS

Continued

REAGENT or RESOURCE	SOURCE	IDENTIFIER
scITD v1.0.2	open source	https://github.com/kharchenkolab/scITD
Seurat v4.0.3	open source	https://satijalab.org/seurat/
SeuratObject v4.0.2	open source	https://cran.r-project.org/web/packages/SeuratObject/index.html
SingleCellExperiment v1.10.1	open source	https://bioconductor.org/packages/release/bioc/html/SingleCellExperiment.html
Slingshot v1.6.1	open source	https://bioconductor.org/packages/release/bioc/html/slingshot.html
SoupX v1.4.5	open source	https://github.com/constantAmateur/SoupX
STAR v2.7.3a	open source	https://github.com/alexdobin/STAR
WGCNA v1.70-3	open source	https://github.com/cran/WGCNA

RESOURCE AVAILABILITY

Lead contact

Further information and requests for resources and reagents should be directed to and will be fulfilled by the lead contact, Katalin Susztak (ksusztak@pennmedicine.upenn.edu).

Materials availability

This study did not generate new unique reagents.

Data and code availability

- Raw and metadata are available at GEO accession number GSE209821.
- Processed data are available via an interactive website (http://www.susztaklab.com/ZSF1_sGC_snRNA/).
- Suppl. data files are deposited in Zenodo.[49]
- Code to reproduce all parts of the analysis is deposited in GitHub (https://github.com/ms-balzer/ZSF1_sGC/).[50]
- Any additional information required to reanalyze the data reported in this work is available from the lead contact upon request.

EXPERIMENTAL MODEL AND SUBJECT DETAILS

ZSF1 rat model

Male ZSF1 lean and ZSF1 obese rats (ZSF1-$Lepr^{fa}Lepr^{cp}$/Crl) were obtained from Charles River Laboratories Inc. (251 Ballardvale St, Wilmington, Massachusetts). The animals were housed in a temperature- (22 ± 2°C) and humidity-controlled environment with a 12h light/dark cycle. Access to water and high energy rodent chow Purina 5008 was provided *ad libitum*. Animal studies were conducted at the Wuppertal Research Center of Bayer AG. The protocol was approved by the institutional animal care and use committee of Bayer AG and was in compliance with the guidelines of the local animal welfare authorities for the German state of North-Rhine Westphalia (Landesamt für Natur, Umwelt und Verbraucherschutz (LANUV) Nordrhein-Westfalen; N0400a022). At the age of 13-14 weeks, ZSF1 obese rats were randomly assigned to a 12-week daily treatment with vehicle (10% ethanol, 40% Kolliphor® HS15, and 50% water), 3 mg/kg BID sGCact = BAY 1101042 = Runcaciguat, or 3 mg/kg QD sGCstim = BAY-747); n=10 each. ZSF1 lean rats (n=6) were not treated and served as controls. In week 12, urine collection was performed in metabolic cages for 6–8 h. At the end of the study, animals were kept in deep anesthesia (isoflurane, 5–10%) and first, blood was collected from peripheral veins to obtain serum and plasma. Then, animals were sacrificed by exsanguination via a cut of axillary vessels. Kidneys were harvested, weighed, rinsed, and then fixed for histological evaluation or immediately snap frozen for single nuclei sequencing.

Human sample procurement

The collection of human kidney tissue was approved by the University of Pennsylvania institutional review board. Un-affected portions of nephrectomies mostly due to malignancy were obtained. Consent was exempted because the samples collected were considered as medical discard. An honest broker collected the related clinical information from chart reviews. Part of the collected tissues was formalin-fixed and paraffin-embedded and sectioned and stained with periodic acid–Schiff. Unbiased pathological scoring of glomerular, interstitial, and vascular parameters was done by a local renal pathologist.

METHOD DETAILS

ZSF1 rat model

Functional parameters and biomarkers

Using the ADVIA Chemistry XPT Systems (Siemens Healthineers), the following parameters were measured: total protein, albumin, and creatinine in urine from day 77; creatinine, urea, and plasma HbA1c from serum and plasma taken on day 84. Total cholesterol also from day 84 serum was measured by Cobas 6000 analyzer series module c501 (Roche Diagnostics). All urinary parameters and biomarkers were normalized to corresponding urinary creatinine values. With regard to biomarkers, all assays were performed according to manufacturers' instructions: Plasma KIM-1 and neutrophil gelatinase-associated lipocalin (NGAL) were measured using a customized Rat Kidney Injury Panel (Meso Scale Discovery).

Histopathological analysis. Kidney samples for histology were fixed in Davidson's solution and embedded in paraffin. Paraffin sections (5 μm) were prepared and stained with hematoxylin and eosin (HE), periodic acid–Schiff (PAS) and Sirius Red/Fast Green (SR/FG). The slides were analyzed using a semiquantitative scoring, ranging from grade 1 to 5 (grade 1, minimal/very few; grade 2, slight/few/small; grade 3, moderate; grade 4, marked/many; grade 5, massive). The grading was applied for each of the predominant kidney lesions like glomerulopathy, tubular degeneration, protein casts, and interstitial fibrosis by a certified pathologist, who conducted the histopathologic examination without the knowledge of treatment assignment but with the overall knowledge of the study design. For the grading of glomerulopathy, altered glomeruli were counted on the PAS slide and the fraction of altered glomeruli was calculated. The mean glomeruli count was determined upfront by counting the glomeruli in 8 of 10 obese ZSF1 rats and averaging the total number. The severity scores represent the percentage of altered glomeruli (up to 5% = grade 1; 5-10% = grade 2; 10-20% = grade 3; 20-30% = grade 4; >30% = grade 5). All other predominant kidney lesions (tubular degeneration, protein casts, and interstitial fibrosis) were graded according to the described scoring system (grades 1-5; grade 1 = minimal/very few lesions; grade 2 = slight/few/small lesions; grade 3 = moderate lesions; grade 4 = marked/many lesions; grade 5 = massive lesions) without any further counting/measuring.

Olink proteomic analyses. The proximity extension assay (PEA) system from Olink (Uppsala, Sweden) was used to measure the mouse exploratory panel containing 96 protein analytes in plasma of ZSF1 rat samples. A detailed protocol has been described previously.[51] In brief, 1 μL of biosample, negative control or interplate control samples was analyzed with 3 μL of incubation mix in a 96-well plate and incubated with the extension mix in a thermal cycler. The measurement real-time PCR was run using a 96.96 Dynamic Array IFC in the Fluidigm BioMark system (Fluidigm). Data were expressed as normalized protein expression (NPX) values after processing and qualification by normalization using the extension control, interpolate control and a correction faction with the Olink NPX manager.

Preparation of rat single-nuclei suspension

Kidneys were harvested, cut into quarters, snap frozen and stored at -80 °C for further analysis. Nuclei were isolated using lysis buffer containing 50% ST buffer (292 mM NaCl, 20 mM Tris-HCl pH 7.5, 2 mM $CaCl_2$ and 42 mM $MgCl_2$ in ultrapure water), 2% Nonidet P40 Substitute, 0.2% ultrapure BSA (50 mg/mL, AM2616, Thermofisher Scietific) and 1% Protector RNase inhibitor (3335399001, Sigma Aldrich). 10-30 mg of frozen kidney tissue was minced with a razor blade into 1-2 mm pieces in 1 mL of lysis buffer. Then, chopped tissue was transferred to a dounce homogenizer. After adding 1 mL of lysis buffer tissue was homogenized using pestle A and B (10 times each). The homogenized tissue was filtered through a 40 μm strainer (08-771-1, Fisher Scientific) and the strainer was washed with 2 mL wash buffer (containing 50% ST buffer, 0.2% ultrapure BSA 50 mg/mL, and 1% protector RNase inhibitor). The washed content was centrifuged at 500 g for 5 minutes at 4 °C. Next, the pellet was resuspended in wash buffer, filtered through a 40 μm Flowmi cell strainer (BAH136800040-50EA, Sigma Aldrich). Intact nuclei shape was confirmed under a microscope, and nuclei were counted.

Rat single-nuclei RNA-seq

10,000 nuclei were loaded into the Chromium Controller (10X Genomics, PN-120223) on a Chromium Next GEM chip G Single Cell Kit (10X Genomics, PN-1000120) to generate single-cell gel beads in the emulsion (GEM) according to the manufacturer's protocol (10X Genomics, PN-1000121). The cDNA and library were made using the Chromium Next GEM Single Cell 3′ GEM Kit v3.1 (10X Genomics, PN-1000121) and Single Index Kit T Set A (10X Genomics, PN-120262) according to the manufacturer's protocol. Quality control for the libraries was performed using Agilent Bioanalyzer High Sensitivity DNA kit (Agilent Technologies, 5067-4626) for qualitative analysis. Libraries were sequenced on an Illumina Novaseq 6000 system with 2 × 150 paired-end kits using the following read length: 28 bp Read1 for cell barcode and UMI, 8 bp I7 index for sample index and 91 bp Read2 for transcript.

Human kidney microdissection and bulk RNA-sequencing

Kidney tissue biopsies were immersed in RNAlater solution (Ambion AM7020) and stored at -80 °C. Specimens were thawed slowly on ice, put into RNAlater, and microdissection was performed manually under a microscope. For every tissue sample, ~80-100 glomeruli were released from their surrounding capsule and the remaining tissue was considered as tubule and was put in RNeasy RNA tissue lysis buffer solution (Qiagen #74106) as per the manufacturer's instructions. The total RNA of 10 mg samples was isolated using Qiagen RNeasy kit (#74106) according to manufacturer's instructions. Agilent Bioanalyzer RNA 6000 Pico kit (Agilent

CellPress
OPEN ACCESS

Technologies #5067-1513) was used to check RNA quality. All samples with an RNA integrity number (RIN) >6 were used for cDNA preparation. Strand specific RNA-seq libraries were generated using TruSeq RNA library prep kit v2 (#RS-122-2001) following the manufacturer's protocol. RNA-seq libraries were sequenced to a depth of 20 million 2 × 150 pair end reads.

Single-nuclei RNA-seq data analysis

Individual sample alignment, ambient RNA correction, and doublet removal. Raw fastq files were aligned and quantified with CellRanger using a custom pre-mRNA GTF built from the ENSEMBL rn6 genome to include intronic regions.

Seurat was used for data quality control, preprocessing, and dimensional reduction. In short, for every sample, a separate gene-cell data matrix was generated and poor-quality cells with <200 or >3,000 expressed genes and mitochondrial gene percentages >15 were excluded. Remaining barcodes of high-quality nuclei were log-normalized and the top 3,000 highly variable genes were identified with the *vst* method. After data scaling, linear dimension reduction was performed using principal component analysis (PCA). A shared nearest neighbor network was created based on Euclidian distances between cells in multidimensional PC space using the first 15 dimensions before clustering using *FindClusters* and dimension reduction using *RunUMAP* functions, respectively.

Doublet-like cells were identified using DoubletFinder.[52] Assuming no ground truth to facilitate an unbiased approach, pK was identified using *paramSweep_v3* function with PCs=1:15. Homotypic doublet proportion was estimated with function *modelHomotypic* using above clustering information. Finally, function *doubletFinder_v3* was run with pN=0.25, pK and nExp as identified by the functions above and Uniform Manifold Approximation and Projections (UMAPs) were manually inspected for singlet/doublet status.

Sample integration and batch correction. After determining high quality ambient RNA-corrected singlet barcodes for every sample individually, 10X filtered output matrices of all 12 samples were again corrected for ambient RNA and subset to singlet barcodes, as determined above, before merging of Seurat objects. The Seurat preprocessing pipeline was then rerun on the merged object (normalization, identification of highly variable genes, scaling, linear dimension reduction), regressing out nCount_RNA during scaling. Harmony[53] was used to correct for potential batch effects. The first 30 Harmony-corrected principal components were used for nearest neighbor network creation, clustering, and dimension reduction. A clustering resolution of 0.9 was chosen to best reflect separate cell identities without artificial over-clustering.

Identification of marker genes and differentially expressed genes. Differentially expressed genes in cell clusters were identified in Seurat using *FindAllMarkers* function with parameters test.use=MAST, min.pct=0.1 and logfc.threshold=0.2 and a manually curated list of marker genes from prior publications[54–60] was used for manual annotation of the 24 resulting cell clusters in the final dataset including 217,132 rat kidney nuclei. Genes differentially expressed between experimental groups were determined with function *FindMarkers* for each cell type separately with the same thresholds.

Integration of ZSF1 rat DKD with human DKD dataset. The ZSF1 rat DKD snRNA-seq dataset was integrated with two independent external human DKD datasets[30,31] using the *FindTransferAnchors* function in Seurat with n=30 dimensions. Nuclei from the human query dataset were projected onto the unimodal ZSF1 rat UMAP with function *MapQuery*. Before correlation analysis of average cluster expression genes from the human DKD dataset were converted to corresponding rat orthologues.

Tensor decomposition. To study the effects of sample stratification across treatment groups and gain a deeper understanding of multicellular gene expression patterns, we used scITD[61] to employ tensor decomposition analysis on our single-nuclei dataset. The SoupX-corrected merged count matrix of the final dataset was provided as input, along with histopathological (interstitial fibrosis, tubular degeneration, hyaline cast, mononuclear infiltration, glomerulopathy scores), functional (proteinuria, as measured by urine protein-to-creatinine ratio), genotype (ZSF1 lean vs. obese), and pharmacological treatment (no treatment, sGC modulator treatment) metadata. Function *form_tensor* was used with parameters donor_min_cells=5, scale_factor=10,000, vargenes_method=norm_var_pvals, vargenes_thresh=0.1, and var_scale_power=2. The number of factors was determined using function *determine_ranks_tucker* with 10 iterations and stability analysis demonstrated mean donor scores correlation >0.9 for all 4 factors. Tucker tensor decomposition was performed using function *run_tucker_ica* with rotation_type=hybrid. Finally, genes significantly associated with each factor were determined with function *get_lm_pvals*.

Gene ontology and pathway analysis. Gene ontology and pathway analyses for gene lists of interest were performed with package clusterProfiler[62] using functions *enrichGO* and *compareCluster*. HALLMARK, GO:BP, C2:KEGG, and C2:CP:PID C2:CP:BIOCARTA gene sets were retrieved through Molecular Signatures Database (MSigDB) v7. For some analyses, GO terms were reduced using package rrvgo functions *calculateSimMatrix* and *reduceSimMatrix*. Reduction of GO terms in semantic space was performed with package GOFigure.

PT and stroma cell subclustering. The whole Seurat pipeline was repeated with the object subset to those barcodes of cells annotated as PT and stroma cells. The same settings were used for the pipeline as stated above. Differential gene expression analysis and subsequent manual annotation revealed that 3 cell identities represented contamination by scattered endothelial and mixed identity clusters and were thus removed from further analyses.

scRNA-seq trajectory analysis

Slingshot. To construct single-cell pseudotime cell trajectories and to identify genes whose expression changed as the cells underwent transition, package Slingshot[63] was applied to a random sample of the following subclusters from the PT and stroma cell dataset, for which UMAP inspection and differential gene expression analysis suggested close transcriptomic proximity: proximal straight tubule (PST), injured PT (PTinj), profibrotic PT (ProfibPT), dedifferentiated PT (DediffPT), interstitial (Int), and mesenchymal cells (Mesench), resulting in a total of 4,821 cells. After Seurat to SingleCellExperiment object conversion, genes were filtered for cell type markers with at least 3 reads in at least 10 cells. Next, counts were normalized and dimensionality was reduced using

diffusion maps with package destiny.[64] Slingshot functions *getLineages* and *getCurves* were used to calculate trajectories. To identify temporally differentially expressed genes, generalized additive modelling (GAM) was applied with a locally estimated scatterplot smoothing (LOESS) term for pseudotime. The top genes were picked based on p value and their expression over pseudotime was visualized in heatmaps after binning pseudotime into quantiles. Genes differentially expressed over pseudotime were input into pathway analysis using package clusterProfiler and pathways specifically enriched over pseudotime bins, as determined by q value calculation, were visualized in heatmaps.

Monocle2 & Monocle3. Slingshot-derived pseudotime trajectories were validated with Monocle2[65] and Monocle3[66] packages using the same cells as input. Genes for ordering cells were selected if they were expressed in $\geq$10 cells, their mean expression value was $\geq$0.05 and dispersion empirical value was $\geq$2. Highly variable genes along pseudotime were identified using *differentialGeneTest* function of Monocle2 with q<0.01. Individual branches were analyzed using *BEAM* and *plot_genes_branched_heatmap* functions. In Monocle3 cells were re-clustered using a resolution of $3e^{-4}$. The trajectory was produced using default parameters of function *learn_graph*. Cluster centers of samples from differentiated PST cells were set as root node before ordering cells along pseudotime with function *order_cells*.

Gene set/pathway scoring. Gene expression of lists or sets of genes was scored in single-cell data as described previously for the cell cycle[67] and other gene sets,[68] using normalized gene expression of a gene set/pathway of interest as input and setting the gene correlation value to 0.1.

Jaccard similarity index. Single-cell cluster stability of PT and stroma cells was evaluated by comparing cluster-specific DEG lists and calculating Jaccard similarity indices according to the following formula:

$$J(A,B) = |A \cap B| / |A \cup B|$$

where J is the Jaccard similarity index and A and B represent DEG lists of two respective clusters to be compared.

Ligand-receptor interactions. To assess cellular crosstalk between different cell types, we used CellChat[69] to infer cell-cell communication networks from single-cell transcriptome data. For the lack of a rat-specific ligand-receptor interaction database, we used orthologous mapping to facilitate usage of the Cellchat-curated mouse database. We followed the authors' tutorial for comparison analysis of multiple datasets (https://htmlpreview.github.io/?https://github.com/sqjin/CellChat/blob/master/tutorial/Comparison_analysis_of_multiple_datasets.html), filtering communication with parameter min.cells=10. We used all inferred cell-cell communications at the level of ligands/receptors and later repeated the analysis focusing on secreted factors and ECM-receptor interactions. Outgoing and incoming interaction weights of pairs of cell types were inferred using functions *computeCommunProbPathway* and *aggregateNet*. Dominant senders and receivers were visualized using functions *netAnalysis_signalingRole_heatmap* and *netAnalysis_signalingRole_scatter*. Structural and functional similarities of signaling pathways were visualized using function *netVisual_embedding*.

Gene regulatory network inference. To identify TFs and characterize cell states, we employed *cis*-regulatory analysis using SCENIC,[70] which infers the GRN based on co-expression and DNA motif analysis. In short, TFs were identified using GENIE3 and compiled into modules (regulons), which were subsequently subjected to *cis*-regulatory motif analysis using RcisTarget with two gene-motif rankings: 10 kb around the TSS and 500 bp upstream. Regulon activity in every cell was then scored using AUCell. Finally, binarized regulon activity was projected onto diffusion map-embedded trajectories.

Weighted gene coexpression network analysis (WGCNA). We applied WGCNA to our scRNA-seq dataset using the R package WGCNA, as described previously.[71,72] First, to circumvent the sparsity of single-cell data we constructed metanuclei with a bootstrapped aggregation process to single-cell transcriptomes and pooled nuclei within the same cell type to retain these metadata for WGCNA. We then created a similarity matrix, in which the similarity between genes reflects the sign of the correlation of their expression profiles. To emphasize strong correlations and reduce the emphasis of weak correlations on an exponential scale, we raised the signed similarity matrix to power β. The resulting adjacency matrix was transformed into a topological overlap matrix. Modules were defined using the following specific module-cutting parameters: module size=50 genes, deepSplit score=4, threshold of correlation=0.2. Modules with a correlation of >0.8 were . The first principal component of the module, the module eigengene (ME), was used to correlate with cell type. Hub genes were defined using intra-modular connectivity (kME) parameters of the WGCNA package.

Bulk RNA-seq data analysis

Quality control and alignment. Adaptor and lower-quality bases were trimmed with Trim-galore. Reads were aligned to the human genome (hg19/GRCh37) using STAR. Gene and isoform expression levels (TPM) were estimated using RSEM. Principal component analysis was performed to identify outliers.

Hierarchical clustering analysis. To identify potential clustering of microdissected human kidney tubule bulk RNA-seq samples based on gene co-expression with sGC, hierarchical clustering was performed on the scaled TPM matrix of 991 microdissected human kidney tubules with the composite WGCNA score as input. Ward's method with Euclidean distances was used to cluster the datasets using function *hclust*. The optimal number of clusters k was determined by average silhouette method. After clustering, a cluster dendrogram was computed. Clinical and histopathological variables were compared between the clustered samples using Wilcoxon-Mann-Whitney, t or Fisher's exact test, as applicable. To exclude random clustering effects and demonstrate validity of the composite WGCNA score as input for clustering analysis, we repeated the above procedure with n=3 randomly generated gene sets with an equal number of genes. In each instance, patient samples were clustered into 2 main clusters based

on gene expression but failed to demonstrate significant differences of DKD prevalence and outcome measures (e.g., proteinuria, GFR, interstitial fibrosis, glomerulosclerosis).

Multiple linear and ordinal logistic regression. To estimate the relative contribution of WGCNA score to disease-relevant parameters in bulk RNA-seq data from human kidney samples, we built separate multiple regression models with fibrosis, glomerulosclerosis, and GFR as dependent variable. Age, gender, race, systolic blood pressure, prevalence of diabetes, BMI, and HgbA1c were put into the models as independent variables. Independent variables were then reduced depending on whether they were informative for the model or not. In multiple regression models, β coefficients and F statistics were calculated. For albuminuria, we performed ordinal logistic regression using the MASS package with function *polr* and computed odds ratios as well as predicted probabilities. The proportional odds assumption was confirmed.

QUANTIFICATION AND STATISTICAL ANALYSIS

Data are expressed as means ± SEM unless otherwise stated. Statistical analyses are indicated in the respective methods sections and figure legends. Appropriate parametric or non-parametric tests were performed as per normality distribution. $P < 0.05$ was considered to be statistically significant. No statistical method was used to predetermine sample size. No data were excluded from the analyses.

ADDITIONAL RESOURCES

We present our data via an easy-to-use interactive web interface at http://www.susztaklab.com/ZSF1_sGC_snRNA/.

Cell Reports Medicine, Volume *4*

Supplemental information

Treatment effects of soluble guanylate cyclase modulation on diabetic kidney disease at single-cell resolution

Michael S. Balzer, Mira Pavkovic, Julia Frederick, Amin Abedini, Alexius Freyberger, Julia Vienenkötter, Ilka Mathar, Krystyna Siudak, Frank Eitner, Peter Sandner, Manuel Grundmann, and Katalin Susztak

Supplementary Information

Treatment effects of soluble guanylate cyclase modulation on diabetic kidney disease at single-cell resolution

Michael S. Balzer[1,2,3,4], Mira Pavkovic[5], Julia Frederick[1,2], Amin Abedini[1,2], Alexius Freyberger[5], Julia Vienenkötter[5], Ilka Mathar[5], Krystyna Siudak[5], Frank Eitner[5,6], Peter Sandner[5,7], Manuel Grundmann[5], and Katalin Susztak[1,2,8,9]

[1]Renal, Electrolyte, and Hypertension Division, Department of Medicine, Perelman School of Medicine, University of Pennsylvania, Philadelphia, PA 19104, USA
[2]Institute for Diabetes, Obesity and Metabolism, Perelman School of Medicine, University of Pennsylvania, Philadelphia, PA 19104, USA
[3]Department of Nephrology and Medical Intensive Care, Charité – Universitätsmedizin Berlin, 10117 Berlin, Germany
[4]Berlin Institute of Health at Charité – Universitätsmedizin Berlin, BIH Biomedical Innovation Academy, BIH Charité Clinician Scientist Program, 10117 Berlin, Germany
[5]Bayer AG, Research and Early Development, Pharma Research Center, 42096 Wuppertal, Germany
[6]Division of Nephrology and Clinical Immunology, RWTH Aachen University, 52062 Aachen, Germany
[7]Department of Pharmacology, Hannover Medical School, 30625 Hannover, Germany
[8]Department of Genetics, Perelman School of Medicine, University of Pennsylvania, Philadelphia, PA 19104, USA

[9]Lead contact
Katalin Susztak, MD, PhD
Professor of Medicine
University of Pennsylvania
Perelman School of Medicine
3400 Civic Center Blvd
Smilow Translational Research Center 12-123
Philadelphia, PA 19104
Phone: (215) 898-2009
ksusztak@pennmedicine.upenn.edu

Supplementary Table

Page 2

Supplementary Figures

Pages 3-30

Table S1. Blood pressure effects of sGC activation in the ZSF1 rat. Related to Figure 1.

Treatment	Baseline MAP (mmHg)	Post-application MAP (mmHg)
Vehicle	120.4 ± 4.8	112.6 ± 1.9
Runcaciguat (3 mg/kg)	122.4 ± 4.0	110.7 ± 1.8

Mean arterial blood pressure (MAP) measured in mmHg with telemetric implants in ZSF1 rats treated with either vehicle (n=6) or 3 mg/kg runcaciguat (n=5); data are shown as mean ± SEM registered over 1 hour before the application for baseline (hour -1 to 0) and 5 hours after application (hour +1 to +6).

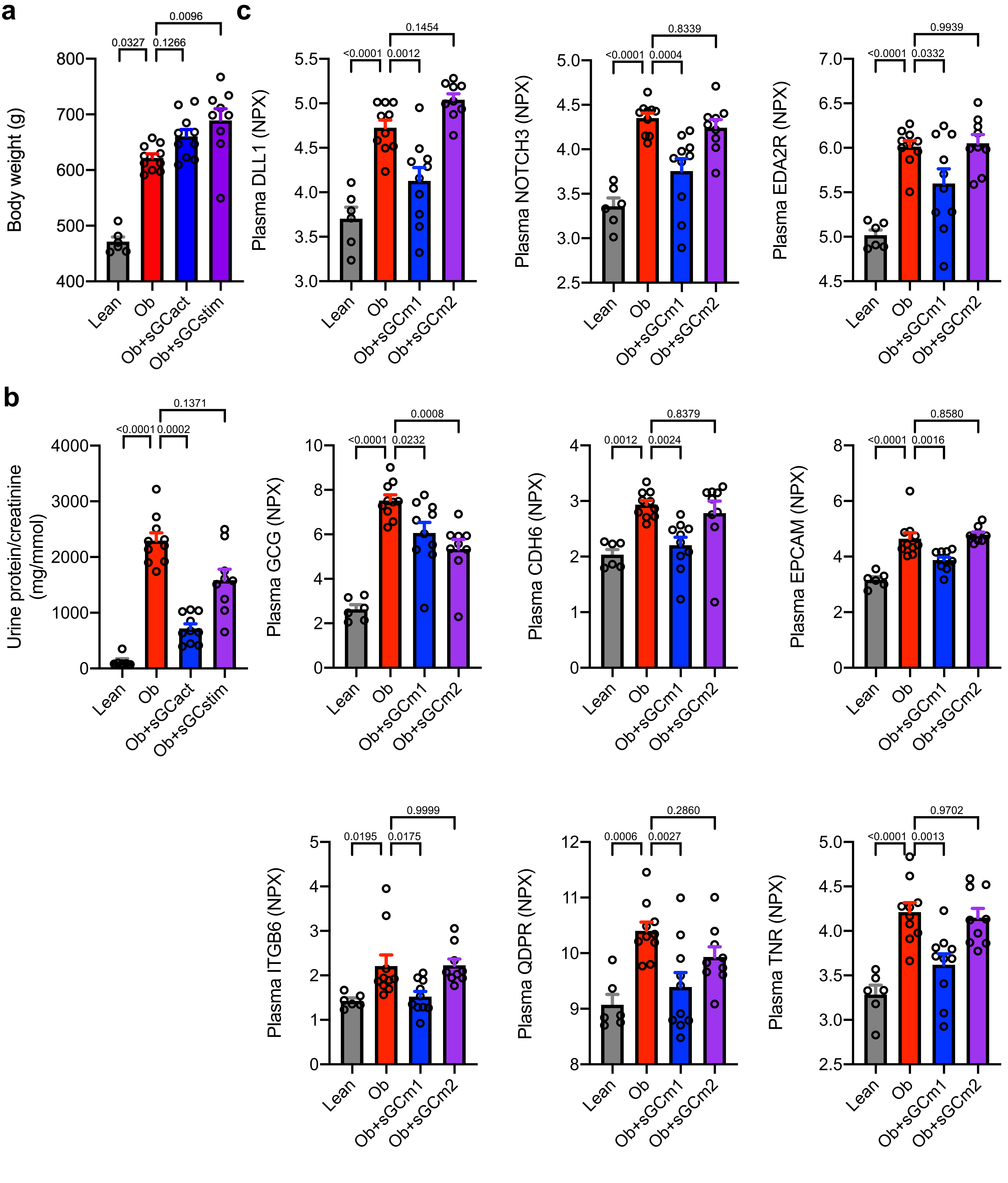

a
b
c
Body weight (g)
Urine protein/creatinine (mg/mmol)
0.0327
0.1266
0.0096
<0.0001
0.0002
0.1371
Lean
Ob
Ob+sGCact
Ob+sGCstim
Plasma DLL1 (NPX)
Plasma NOTCH3 (NPX)
Plasma EDA2R (NPX)
Plasma GCG (NPX)
Plasma CDH6 (NPX)
Plasma EPCAM (NPX)
Plasma ITGB6 (NPX)
Plasma QDPR (NPX)
Plasma TNR (NPX)
<0.0001
0.0012
0.1454
<0.0001
0.0004
0.8339
<0.0001
0.0332
0.9939
<0.0001
0.0232
0.0008
0.0012
0.0024
0.8379
<0.0001
0.0016
0.8580
0.0195
0.0175
0.9999
0.0006
0.0027
0.2860
<0.0001
0.0013
0.9702
Ob+sGCm1
Ob+sGCm2

Figure S1. Diabetic ZSF1 rats recapitulate phenotypic changes of DKD with marked disease improvement by sGC activators. Related to Figure 1.

(a) Body weight; p values are given for Kruskal-Wallis test (Benjamini, Krieger, Yekutieli-corrected); Ob, Obese.

(b) Urinary protein/creatinine ratio; p values are given for Kruskal-Wallis test (Benjamini, Krieger, Yekutieli-corrected); Ob, Obese.

(c) Explorative proteomics analysis of 92 plasma proteins using a multiplexed proximity extension assay (Olink); p values are given for one-way ANOVA (Benjamini, Krieger, Yekutieli-corrected); NPX, normalized protein expression; Ob, Obese.

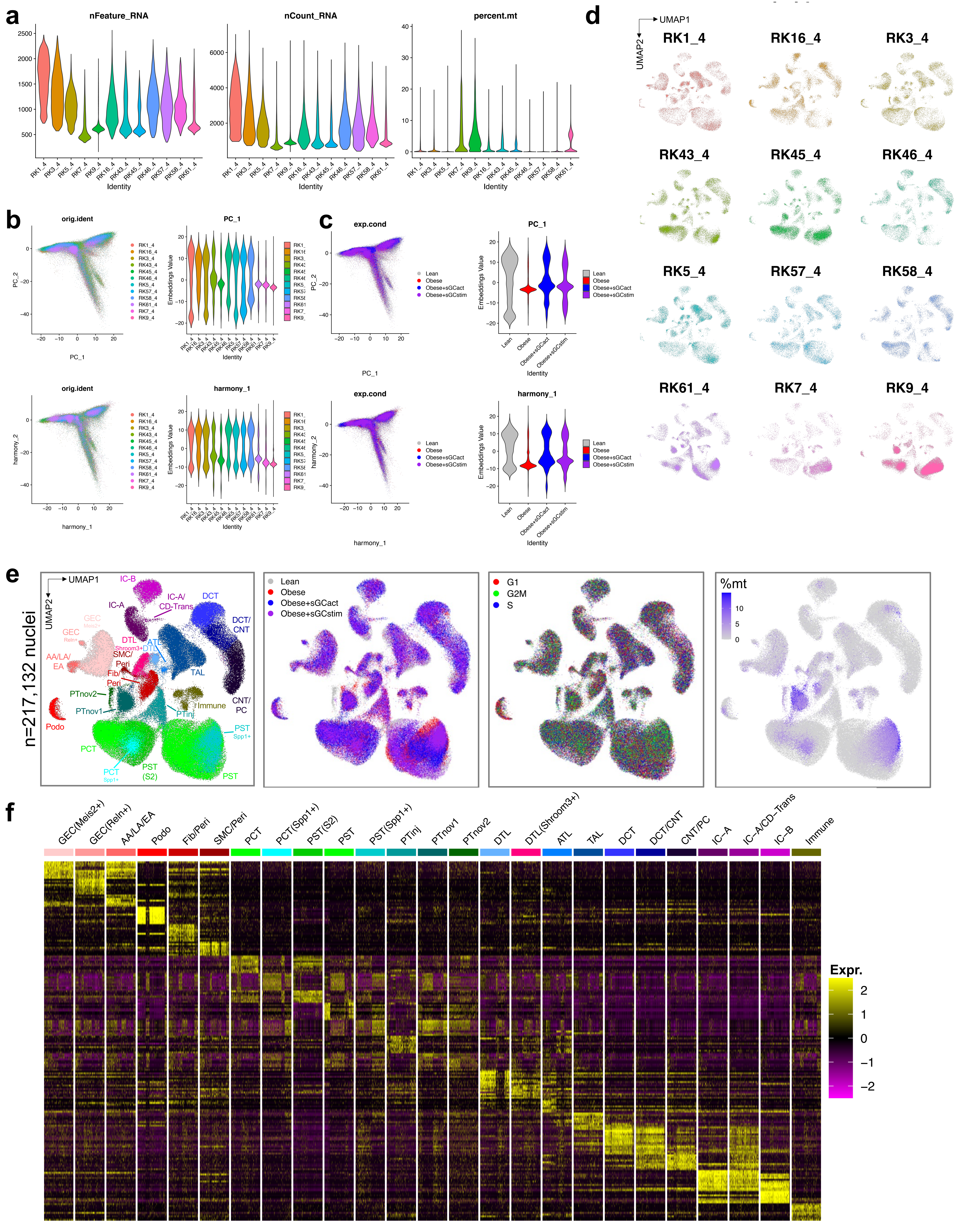
a
nFeature_RNA
nCount_RNA
percent.mt
Identity
b
orig.ident
PC_1
PC_2
Embeddings Value
harmony_1
harmony_2
c
exp.cond
Lean
Obese
Obese+sGCact
Obese+sGCstim
d
UMAP1
UMAP2
RK1_4
RK16_4
RK3_4
RK43_4
RK45_4
RK46_4
RK5_4
RK57_4
RK58_4
RK61_4
RK7_4
RK9_4
e
n=217,132 nuclei
IC-B
IC-A
IC-A/ CD-Trans
DCT
DCT/ CNT
GEC Meis2+
GEC Reln+
AA/LA/ EA
DTL Shroom3+
ATL
DTL
SMC/ Peri
Fib/ Peri
TAL
CNT/ PC
PTnov2
PTnov1
Immune
PTinj
Podo
PST Spp1+
PCT
PCT Spp1+
PST (S2)
PST
G1
G2M
S
%mt
f
GEC(Meis2+)
GEC(Reln+)
AA/LA/EA
Podo
Fib/Peri
SMC/Peri
PCT
PCT(Spp1+)
PST(S2)
PST
PST(Spp1+)
PTinj
PTnov1
PTnov2
DTL
DTL(Shroom3+)
ATL
TAL
DCT
DCT/CNT
CNT/PC
IC-A
IC-A/CD-Trans
IC-B
Immune
Expr.

Figure S2. Single-cell transcriptomic landscape of the diabetic ZSF1 rat. Related to Figure 2.

(a) Violin plots showing number of informative genes (nFeature_RNA), unique molecular identifiers (nCount_RNA), and percentage of mitochondrial genes (percent.mt) per single cell.

(b) Principal component (PC) representation (left) and violin plots of corresponding embeddings values (right) before (top) and after batch correction (bottom) using Harmony, stratified by individual sample.

(c) Same as in **(b)**, stratified by experimental group.

(d) Integrated UMAP corresponding to **Fig. 2a**, stratified by sample.

(e) Integrated UMAP corresponding to **Fig. 2a**, annotated by low-level clustering, experimental group, cell cycle phase, and mitochondrial percentage (%mt), respectively; GEC, glomerular endothelial cell; AA/LA/EA, afferent arteriole/large artery/efferent arteriole; Podo, podocyte; Fib, fibroblast; Peri, pericyte; SMC; smooth muscle cell; PCT, proximal convoluted tubule; PST(S2); proximal straight tubule (segment 2); PTinj, injured proximal tubule; PTnov, novel PT; DTL, descending thin limb; ATL, ascending thin limb; TAL, thick ascending limb; DCT, distal convoluted tubule; CNT, connecting tubule; PC, collecting duct principal cell; IC-A, collecting duct intercalated cell type A; CD-Trans, collecting duct transitional cell; IC-B, collecting duct intercalated cell type B; Immune, immune cell.

(f) Heatmap of top 10 differentially expressed genes for low-level clustering, corresponding to **Fig. 2b**.

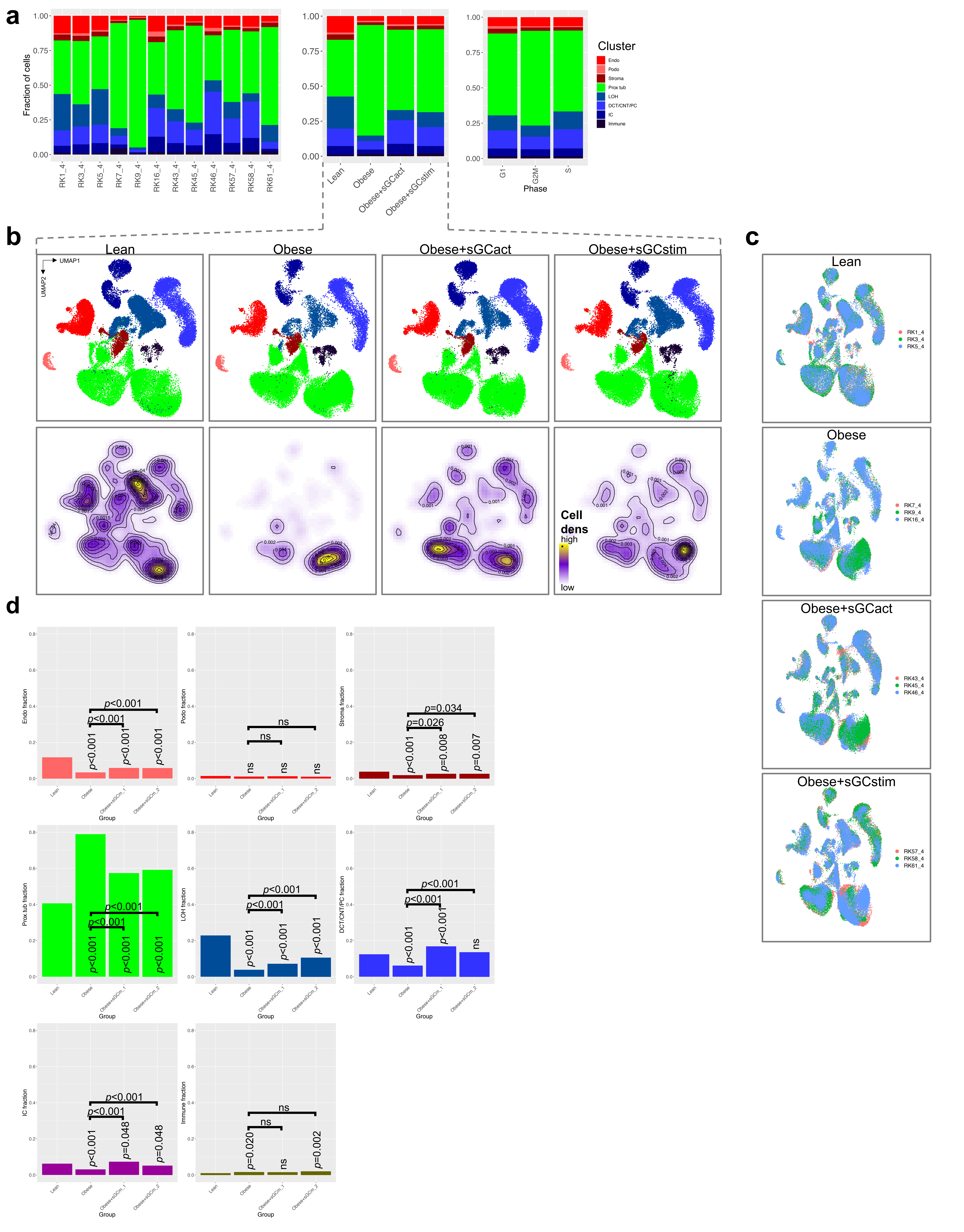
a
b
c
d
Fraction of cells
RK1_4 RK3_4 RK5_4 RK7_4 RK9_4 RK16_4 RK43_4 RK45_4 RK46_4 RK57_4 RK58_4 RK61_4
Lean Obese Obese+sGCact Obese+sGCstim
G1 G2M S
Phase
Cluster
Endo
Podo
Stroma
Prox tub
LOH
DCT/CNT/PC
IC
Immune
Lean
Obese
Obese+sGCact
Obese+sGCstim
UMAP1
UMAP2
Cell dens
high
low
RK1_4 RK3_4 RK5_4
RK7_4 RK9_4 RK16_4
RK43_4 RK45_4 RK46_4
RK57_4 RK58_4 RK61_4
Endo fraction
Podo fraction
Stroma fraction
Prox.tub fraction
LOH fraction
DCT/CNT/PC fraction
IC fraction
Immune fraction
Group
Lean Obese Obese+sGCm_1 Obese+sGCm_2

Figure S3. Cell fraction changes. Related to Figure 2.

(a) Fractions of high-level clustering cell types across samples, experimental groups, and cell cycle phase, respectively; Endo, endothelial cell; Podo, podocyte; Stroma, stromal cell; Prox tub, proximal tubule; LOH, loop of Henle; DCT/CNT/PC, distal convoluted tubule/connecting tubule/collecting duct principal cell; IC, collecting duct intercalated cell; Immune, immune cell.

(b) Integrated UMAP (top) and density plots (bottom) corresponding to **Fig. 2a**, stratified by experimental group.

(c) Integrated UMAP stratified by experimental group and colored by individual sample demonstrates negligible within-group heterogeneity.

(d) Cell fractions of high-level clusters stratified by experimental group. Statistical significance for comparisons was derived using differential proportion analysis, with a mean error of 0.1 over 100,000 iterations; p values are given for comparisons between Lean and (un-)treated Obese groups and between untreated Obese and treated Obese groups, respectively; ns, not significant.

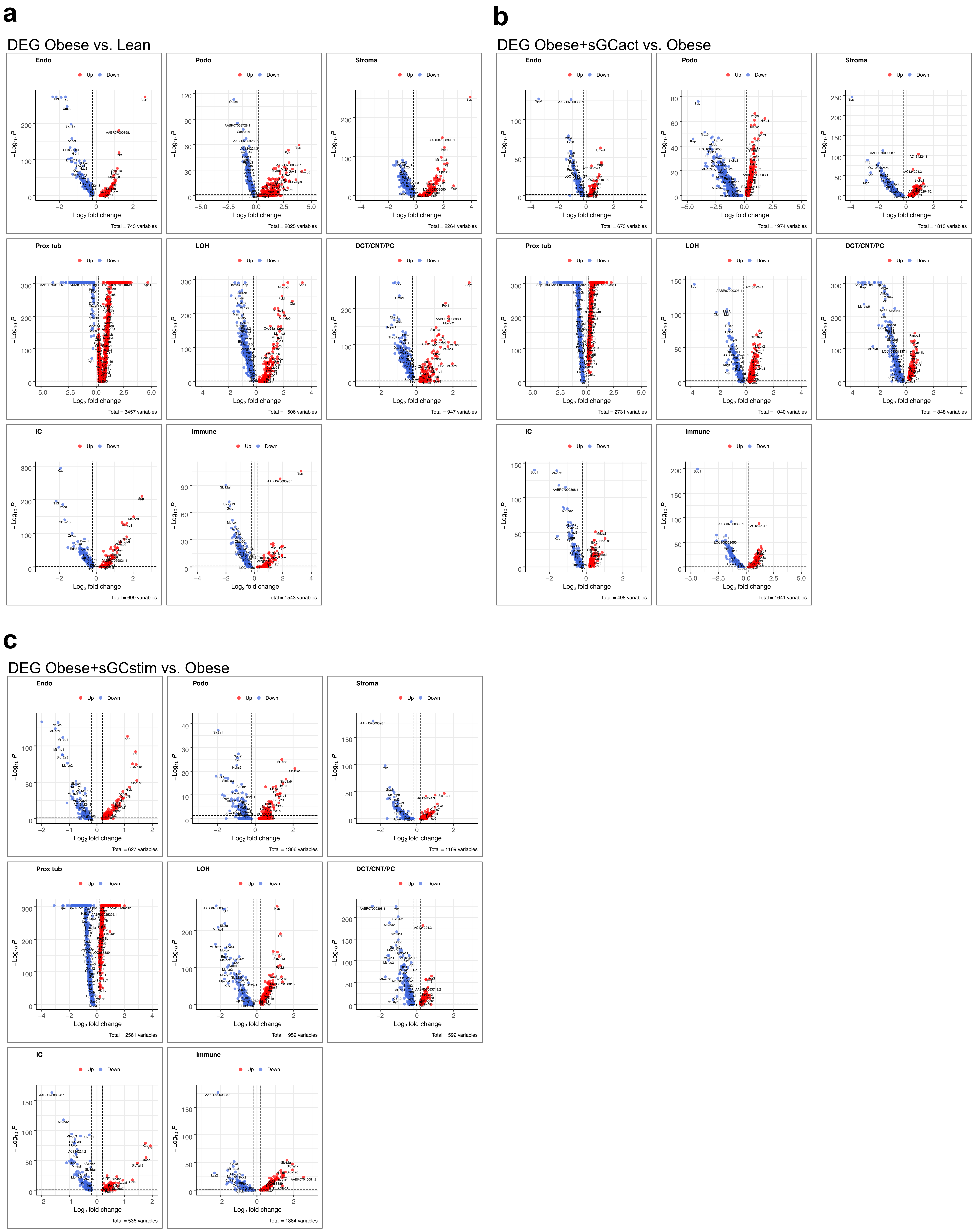
a
DEG Obese vs. Lean
Endo
Podo
Stroma
Prox tub
LOH
DCT/CNT/PC
IC
Immune
Up
Down
Log2 fold change
−Log10 P
Total = 743 variables
Total = 2025 variables
Total = 2264 variables
Total = 3457 variables
Total = 1506 variables
Total = 947 variables
Total = 699 variables
Total = 1543 variables
b
DEG Obese+sGCact vs. Obese
Total = 673 variables
Total = 1974 variables
Total = 1813 variables
Total = 2731 variables
Total = 1040 variables
Total = 848 variables
Total = 498 variables
Total = 1641 variables
c
DEG Obese+sGCstim vs. Obese
Total = 627 variables
Total = 1366 variables
Total = 1169 variables
Total = 2561 variables
Total = 959 variables
Total = 592 variables
Total = 536 variables
Total = 1384 variables

Figure S4. Differentially expressed genes. Related to Figure 2.

(a-c) Volcano plots of differentially expressed genes (DEGs) comparing Obese vs. Lean **(a)**, Obese+sGCact vs. Obese **(b)**, and Obese+sGCstim vs. Obese samples **(c)**, respectively. Comparisons are given separately for high-level cell types. X axes indicate $\log_2$-fold change (Model-based Analysis of Single-cell Transcriptomics, MAST) and y axis indicates statistical significance-adjusted p = $-\log_{10}$. Endo, endothelial cell; Podo, podocyte; Stroma, stromal cell; Prox tub, proximal tubule; LOH, loop of Henle; DCT/CNT/PC, distal convoluted tubule/connecting tubule/collecting duct principal cell; IC, collecting duct intercalated cell; Immune, immune cell.

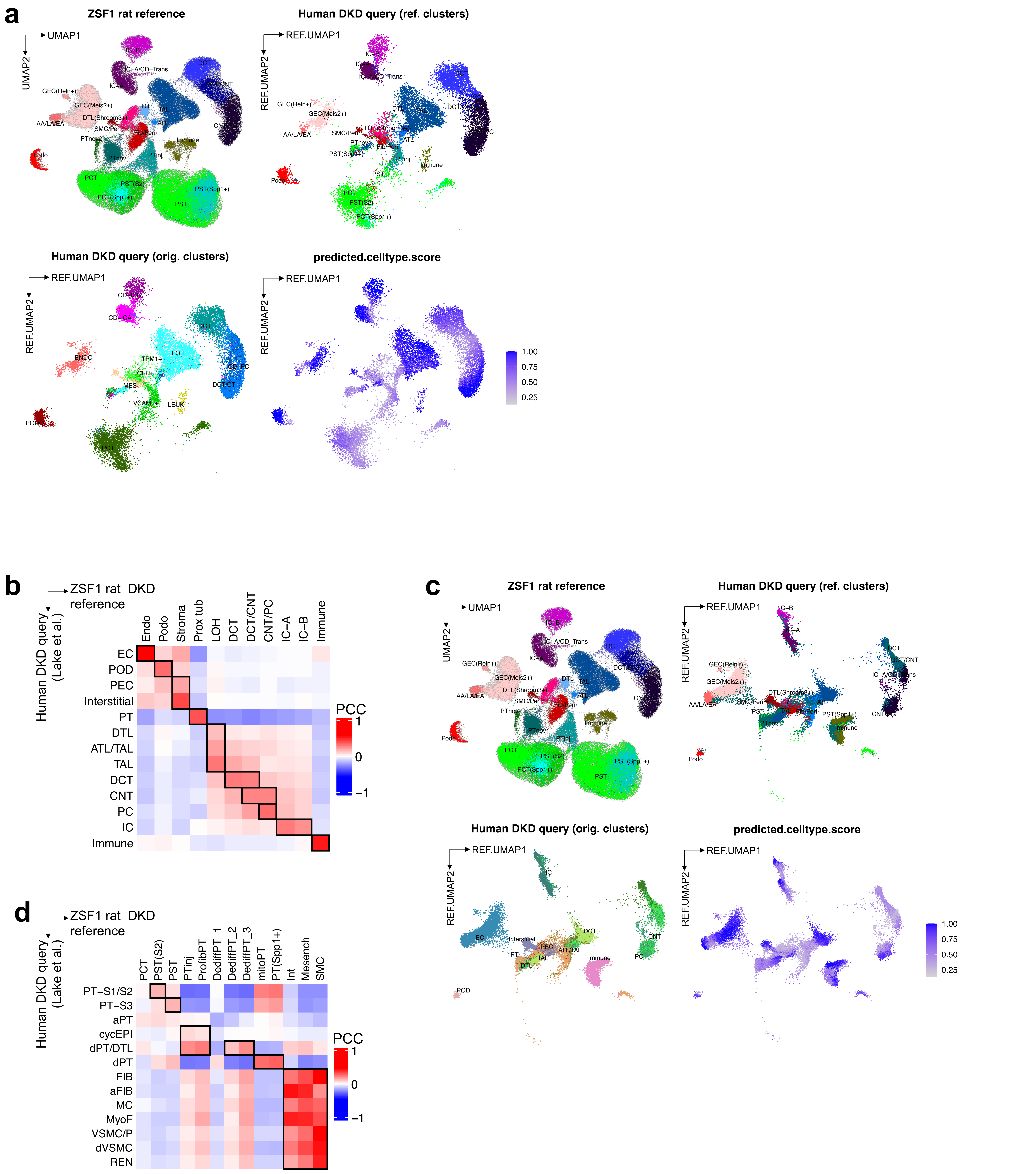
a
ZSF1 rat reference
UMAP1
UMAP2
IC–B
IC–A/CD–Trans
IC–A
DCT
DCT/CNT
CNT
GEC(Reln+)
GEC(Meis2+)
DTL
TAL
ATL
DTL(Shroom3+)
AA/LA/EA
SMC/Peri
Fib/Peri
PTnov2
Immune
Podo
PTnov1
PTinj
PCT
PST(S2)
PCT(Spp1+)
PST(Spp1+)
PST
Human DKD query (ref. clusters)
REF.UMAP1
REF.UMAP2
Human DKD query (orig. clusters)
CD−ICB
CD−ICA
DCT
ENDO
LOH
TPM1+
CFH+
CD−PC
DCT/CT
MES
VCAM1+
LEUK
POD
PCT
predicted.celltype.score
1.00
0.75
0.50
0.25
b
ZSF1 rat DKD reference
Human DKD query (Lake et al.)
Endo
Podo
Stroma
Prox tub
LOH
DCT
DCT/CNT
CNT/PC
IC–A
IC–B
Immune
EC
POD
PEC
Interstitial
PT
DTL
ATL/TAL
TAL
DCT
CNT
PC
IC
Immune
PCC
1
0
−1
c
ZSF1 rat reference
Human DKD query (ref. clusters)
Human DKD query (orig. clusters)
IC
EC
Interstitial
PEC
PT
DTL
TAL
ATL/TAL
DCT
CNT
PC
Immune
POD
predicted.celltype.score
1.00
0.75
0.50
0.25
d
ZSF1 rat DKD reference
Human DKD query (Lake et al.)
PCT
PST(S2)
PST
PTinj
ProfibPT
DediffPT_1
DediffPT_2
DediffPT_3
mitoPT
PT(Spp1+)
Int
Mesench
SMC
PT−S1/S2
PT−S3
aPT
cycEPI
dPT/DTL
dPT
FIB
aFIB
MC
MyoF
VSMC/P
dVSMC
REN
PCC
1
0
−1

Figure S5. ZSF1 rat integration with human DKD. Related to Figure 2.

(a) UMAP representations of ZSF1 rat reference with low-level clustering (top left) and human DKD query dataset (Wilson et al.) projected onto ZSF1 rat reference dataset (other subpanels). Human DKD subpanels are annotated/colored by ZSF1 rat reference clustering (top right), by original clustering (bottom left), and by predicted cell type score (bottom right).

(b) Pearson correlation coefficient (PCC) matrix of average cell type gene expression between ZSF1 rats (Lean and Obese samples only) and a corresponding human snRNA-seq dataset from the Kidney Precision Medicine Project (KPMP, Lake et al.) with Control and DKD kidney samples; ATL, ascending thin limb; CNT, connecting tubule; DCT, distal convoluted tubule; DTL, descending thin limb; Endo/EC, endothelial cell; IC, collecting duct intercalated cell; LEUK, leukocyte; LOH, loop of Henle; MES, mesangial cells; PC, collecting duct principal cell; PEC, parietal epithelial cell; Podo/POD, podocytes; Prox tub/PT, proximal tubule; TAL, thick ascending limb.

(c) Similar UMAP representations as in **(a)**, but with dataset from Kidney Precision Medicine Project (KPMP, Lake et al.).

(d) Similar correlation analysis as in **(b)**, but with PT and stromal cell subclustering. aFIB, "adaptive" fibroblast; aPT, "adaptive epithelial state" proximal tubule; cycEPI, cycling epithelial cells; dPT, "degenerative" proximal tubule; DTL, descending thin limb; dVSCM, "degenerative" vascular smooth muscle cell; FIB, fibroblast; Int, interstitial cell; MC, mesangial cell; Mesench, mesenchymal cell; mitoPT, high mitochondrial gene PT; MyoF, myofibroblast; PCT, proximal convoluted tubule; PST, proximal straight tubule; PT, proximal tubule; PTinj, injured PT; ProfibPT, profibrotic PT; DediffPT, dedifferentiated PT; PT(Spp1+), $Spp1^{+}$ PT; REN, Renin-positive juxtaglomerular granular cell; SMC, smooth muscle cell; VSCM/P, vascular smooth muscle cell/pericyte.

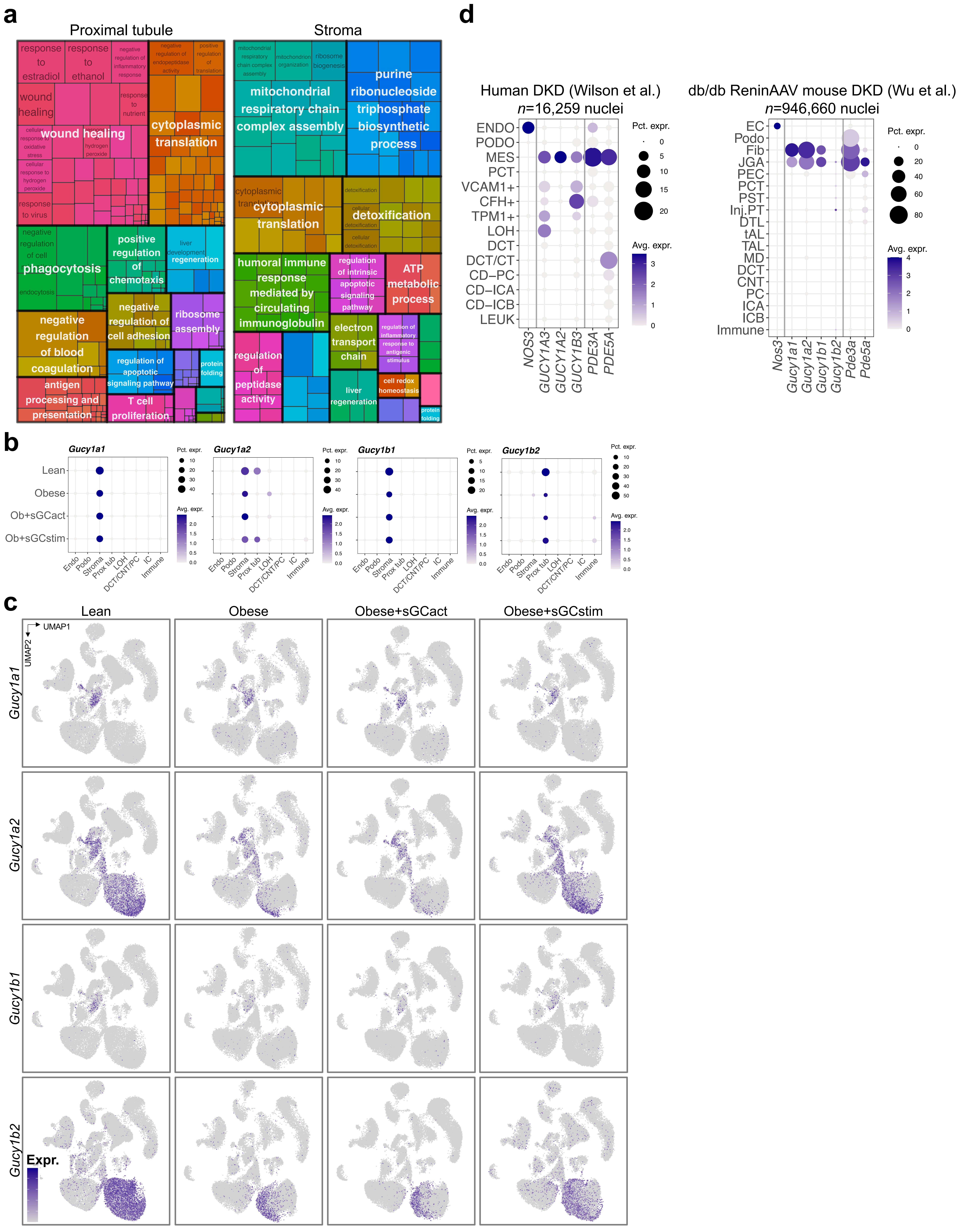
a
Proximal tubule
Stroma
response to estradiol
response to ethanol
wound healing
cytoplasmic translation
phagocytosis
positive regulation of chemotaxis
negative regulation of cell adhesion
ribosome assembly
negative regulation of blood coagulation
antigen processing and presentation
regulation of apoptotic signaling pathway
T cell proliferation
protein folding
liver development regeneration
mitochondrial respiratory chain complex assembly
purine ribonucleoside triphosphate biosynthetic process
detoxification
humoral immune response mediated by circulating immunoglobulin
regulation of intrinsic apoptotic signaling pathway
ATP metabolic process
electron transport chain
regulation of peptidase activity
liver regeneration
cell redox homeostasis
b
Gucy1a1
Gucy1a2
Gucy1b1
Gucy1b2
Lean
Obese
Ob+sGCact
Ob+sGCstim
Endo
Podo
Stroma
Prox tub
LOH
DCT/CNT/PC
IC
Immune
Pct. expr.
Avg. expr.
c
Lean
Obese
Obese+sGCact
Obese+sGCstim
UMAP1
UMAP2
Expr.
d
Human DKD (Wilson et al.)
n=16,259 nuclei
ENDO
PODO
MES
PCT
VCAM1+
CFH+
TPM1+
LOH
DCT
DCT/CT
CD–PC
CD–ICA
CD–ICB
LEUK
NOS3
GUCY1A3
GUCY1A2
GUCY1B3
PDE3A
PDE5A
db/db ReninAAV mouse DKD (Wu et al.)
n=946,660 nuclei
EC
Podo
Fib
JGA
PEC
PCT
PST
Inj.PT
DTL
tAL
TAL
MD
DCT
CNT
PC
ICA
ICB
Immune
Nos3
Gucy1a1
Gucy1a2
Gucy1b1
Gucy1b2
Pde3a
Pde5a

Figure S6. GO term analysis of tensor decomposition factor 1-loading genes and PT and stromal cell type specificity of sGC gene expression. Related to Figure 2.

(a) Gene ontology (GO) terms of factor 1-loading genes in proximal tubule and stromal cells.

(b) Expression dot plots for sGC genes, stratified by experimental group (rows) and high-level clustering (columns); dot size denotes percentage of cells expressing the marker. Color scale represents average gene expression values.

(c) Feature plots corresponding to **(b)**.

(d) Expression dot plots for NO/sGC/cGAMP pathway genes in human DKD (left) and murine DKD (right) datasets, corresponding to **Fig. 2g**; dot size denotes percentage of cells expressing the marker. Color scale represents average gene expression values.

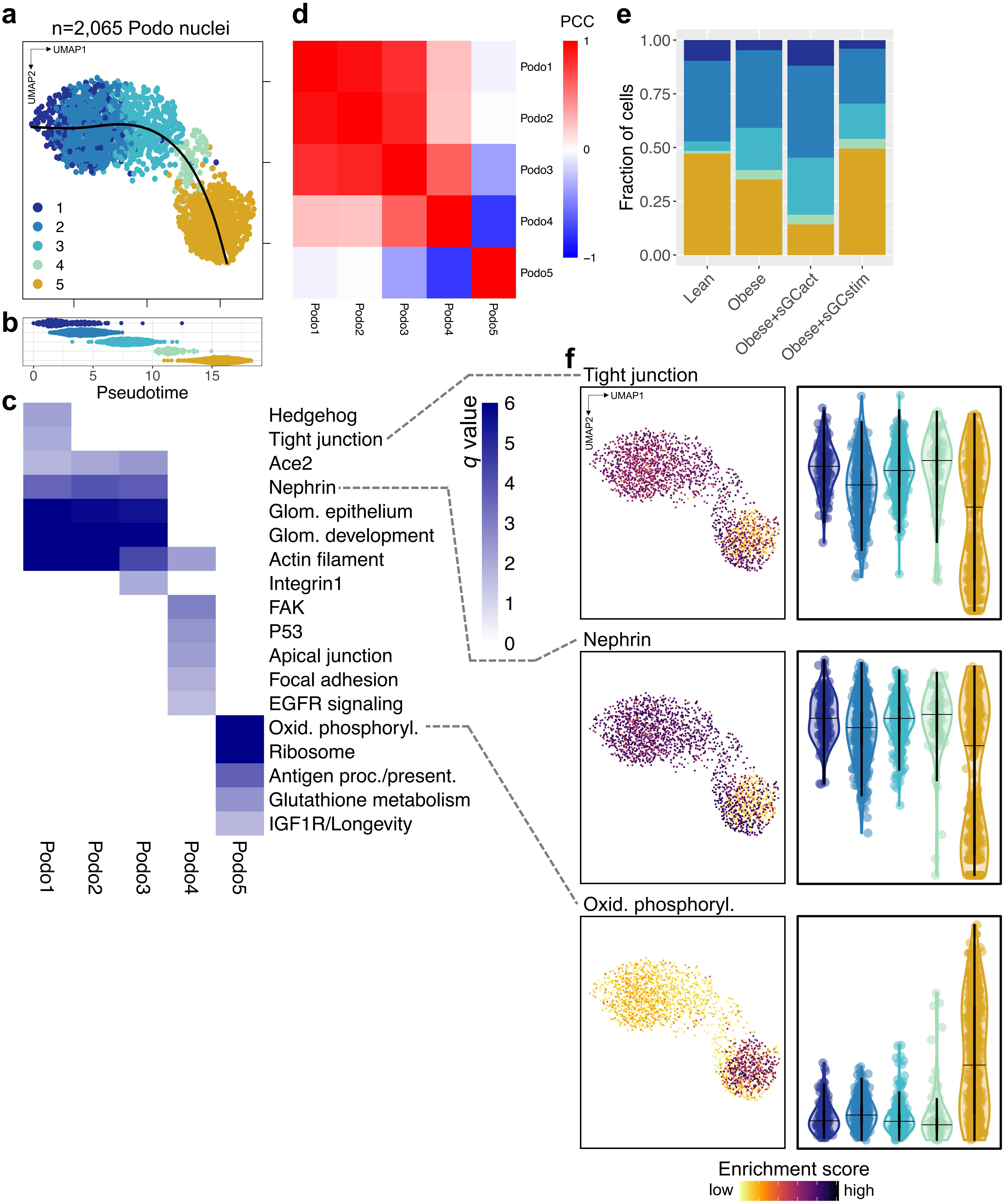

a
n=2,065 Podo nuclei
UMAP1
UMAP2
1
2
3
4
5
b
0
5
10
15
Pseudotime
c
Hedgehog
Tight junction
Ace2
Nephrin
Glom. epithelium
Glom. development
Actin filament
Integrin1
FAK
P53
Apical junction
Focal adhesion
EGFR signaling
Oxid. phosphoryl.
Ribosome
Antigen proc./present.
Glutathione metabolism
IGF1R/Longevity
Podo1
Podo2
Podo3
Podo4
Podo5
q value
6
5
4
3
2
1
0
d
PCC
1
0
−1
Podo1
Podo2
Podo3
Podo4
Podo5
e
Fraction of cells
1.00
0.75
0.50
0.25
0.00
Lean
Obese
Obese+sGCact
Obese+sGCstim
f
Tight junction
Nephrin
Oxid. phosphoryl.
Enrichment score
low
high

Figure S7. Podocyte subclustering. Related to Figure 2.

(a) Integrated UMAP of 2,065 high-quality podocyte nuclei from 12 rat kidney samples. Black line indicates Slingshot-inferred trajectory from clusters 1 through 5.

(b) Pseudotime values across podocyte clusters 1 through 5. Colors correspond to **(a)**.

(c) Heatmaps shows enrichment of top pseudotime-specific GO biological processes and KEGG pathways.

(d) Pearson correlation coefficient (PCC) matrix of average cell type gene expression between podocyte clusters 1 through 5.

(e) Cell fractions of podocyte clusters stratified by experimental group. Colors correspond to **(a)**.

(f) Scoring of gene sets corresponding to representative pathways from **(c)**. Left panels show pathway enrichment along the podocyte trajectory. Right panels show gene set scores by podocyte cluster. Colors correspond to **(a)**.

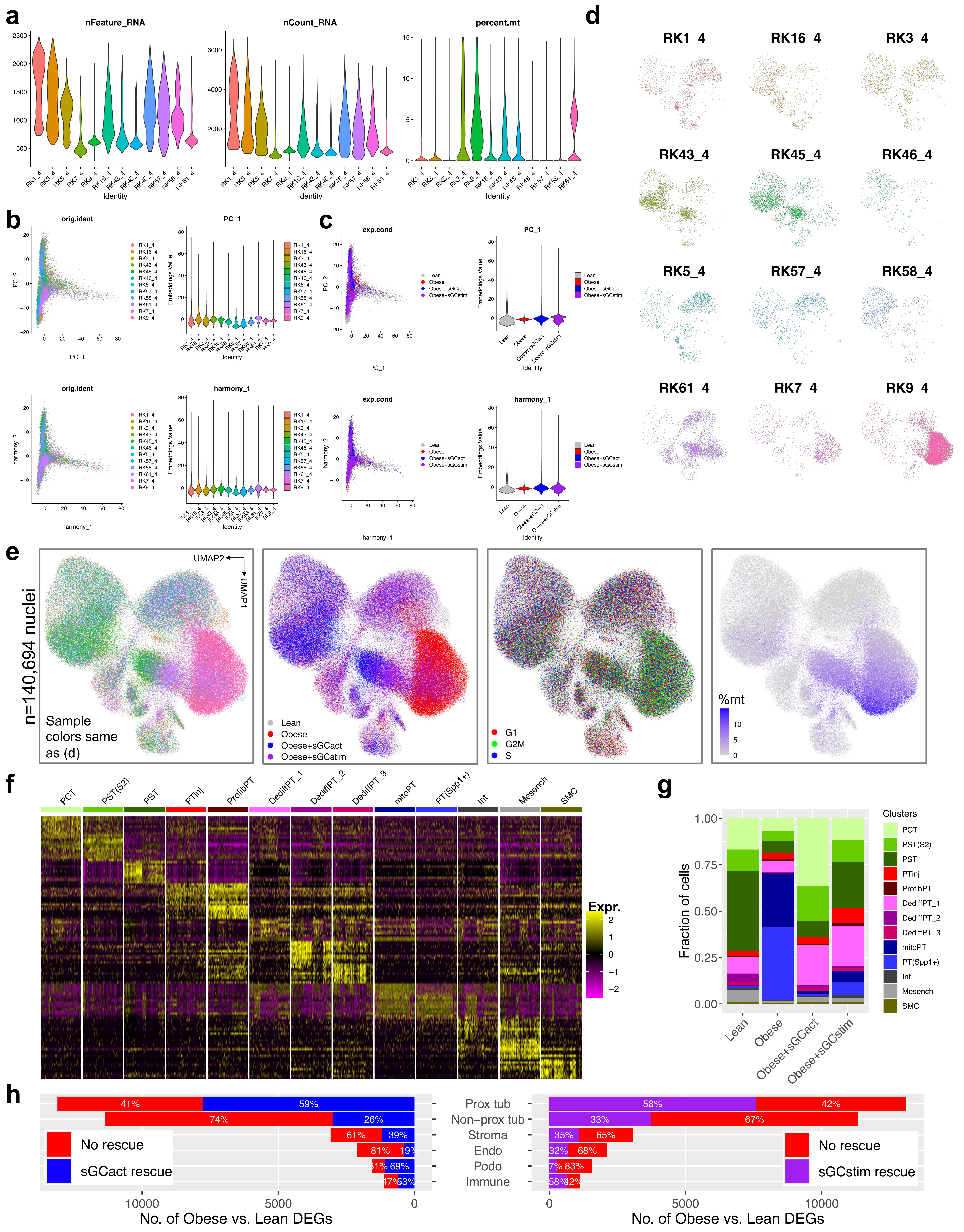

a
nFeature_RNA
nCount_RNA
percent.mt
Identity
RK1_4
RK3_4
RK5_4
RK7_4
RK9_4
RK16_4
RK43_4
RK45_4
RK46_4
RK57_4
RK58_4
RK61_4
b
orig.ident
PC_1
PC_2
Embeddings Value
harmony_1
harmony_2
c
exp.cond
Lean
Obese
Obese+sGCact
Obese+sGCstim
d
RK1_4
RK16_4
RK3_4
RK43_4
RK45_4
RK46_4
RK5_4
RK57_4
RK58_4
RK61_4
RK7_4
RK9_4
e
n=140,694 nuclei
UMAP2
UMAP1
Sample colors same as (d)
Lean
Obese
Obese+sGCact
Obese+sGCstim
G1
G2M
S
%mt
f
PCT
PST(S2)
PST
PTinj
ProfibPT
DediffPT_1
DediffPT_2
DediffPT_3
mitoPT
PT(Spp1+)
Int
Mesench
SMC
Expr.
g
Fraction of cells
Clusters
Lean
Obese
Obese+sGCact
Obese+sGCstim
h
Prox tub
Non−prox tub
Stroma
Endo
Podo
Immune
No rescue
sGCact rescue
sGCstim rescue
No. of Obese vs. Lean DEGs
41%
59%
74%
26%
61%
39%
81%
19%
31%
69%
47%
53%
58%
42%
33%
67%
35%
65%
32%
68%
7%
83%
58%
42%

Figure S8. PT and stromal cell subclustering. Related to Figure 3.

(a) Violin plots showing number of informative genes (nFeature_RNA), unique molecular identifiers (nCount_RNA), and percentage of mitochondrial genes (percent.mt) per single cell.

(b) Principal component (PC) representation (left) and violin plots of corresponding embeddings values (right) before (top) and after batch correction (bottom) using Harmony, stratified by individual sample.

(c) Same as in **(b)**, stratified by experimental group.

(d) Integrated UMAP corresponding to **Fig. 3a**, stratified by sample.

(e) Integrated UMAP corresponding to **Fig. 3a**, annotated by sample, experimental group, cell cycle phase, and mitochondrial percentage (%mt), respectively.

(f) Heatmap of top 10 differentially expressed genes for subclusters. PCT, proximal convoluted tubule; PST(S2), proximal straight tubule (segment 2); PST, proximal straight tubule; PTinj, injured PT; ProfibPT, profibrotic PT; DediffPT, dedifferentiated PT; mitoPT, high mitochondrial gene PT; PT(Spp1+), $Spp1^{+}$ PT; Int, interstitial cell; Mesench, mesenchymal cell; SMC, smooth muscle cell.

(g) Cell fractions of PT and stromal cell subclusters stratified by experimental group. Colors correspond to **(f)**.

(h) Bar graphs representing the number of genes differentially expressed (DEG) between Obese and Lean samples in coarse-grained, high-level cell types. Percentages indicate absent or present rescue effect (normalization) for DEG comparison between sGC modulator-treated rats (sGCstim, sGCact) and vehicle-treated rats.

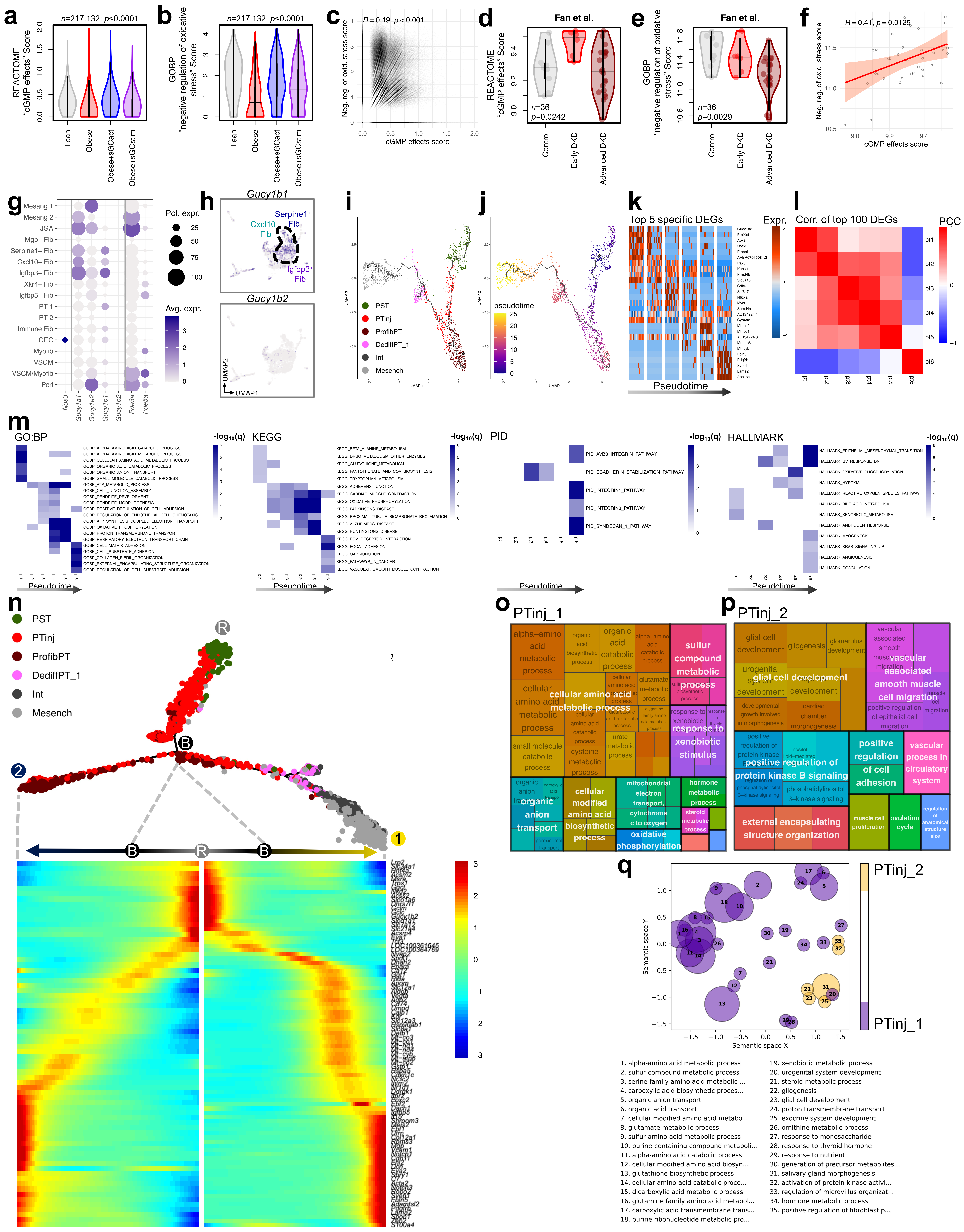

a
n=217,132; p<0.0001
REACTOME "cGMP effects" Score
Lean
Obese
Obese+sGCact
Obese+sGCstim
b
n=217,132; p<0.0001
GOBP "negative regulation of oxidative stress" Score
c
R = 0.19, p < 0.001
Neg. reg. of oxid. stress score
cGMP effects score
d
Fan et al.
REACTOME "cGMP effects" Score
n=36
p=0.0242
Control
Early DKD
Advanced DKD
e
Fan et al.
GOBP "negative regulation of oxidative stress" Score
n=36
p=0.0029
f
R = 0.41, p = 0.0125
Neg. reg. of oxid. stress score
cGMP effects score
g
Mesang 1
Mesang 2
JGA
Mgp+ Fib
Serpine1+ Fib
Cxcl10+ Fib
Igfbp3+ Fib
Xkr4+ Fib
Igfbp5+ Fib
PT 1
PT 2
Immune Fib
GEC
Myofib
VSCM
VSCM/Myofib
Peri
Nos3
Gucy1a1
Gucy1a2
Gucy1b1
Gucy1b2
Pde3a
Pde5a
Pct. expr.
Avg. expr.
h
Gucy1b1
Serpine1+ Fib
Cxcl10+ Fib
Igfbp3+ Fib
Gucy1b2
UMAP2
UMAP1
i
PST
PTinj
ProfibPT
DediffPT_1
Int
Mesench
j
pseudotime
k
Top 5 specific DEGs
Expr.
Pseudotime
l
Corr. of top 100 DEGs
PCC
pt1
pt2
pt3
pt4
pt5
pt6
m
GO:BP
-log10(q)
KEGG
PID
HALLMARK
Pseudotime
n
PST
PTinj
ProfibPT
DediffPT_1
Int
Mesench
o
PTinj_1
sulfur compound metabolic process
cellular amino acid metabolic process
response to xenobiotic stimulus
organic anion transport
cellular modified amino acid biosynthetic process
oxidative phosphorylation
p
PTinj_2
glial cell development
vascular associated smooth muscle cell migration
positive regulation of protein kinase B signaling
positive regulation of cell adhesion
vascular process in circulatory system
external encapsulating structure organization
q
Semantic space X
Semantic space Y
PTinj_2
PTinj_1
1. alpha-amino acid metabolic process
2. sulfur compound metabolic process
3. serine family amino acid metabolic ...
4. carboxylic acid biosynthetic proces...
5. organic anion transport
6. organic acid transport
7. cellular modified amino acid metabo...
8. glutamate metabolic process
9. sulfur amino acid metabolic process
10. purine-containing compound metaboli...
11. alpha-amino acid catabolic process
12. cellular modified amino acid biosyn...
13. glutathione biosynthetic process
14. cellular amino acid catabolic proce...
15. dicarboxylic acid metabolic process
16. glutamine family amino acid metabol...
17. carboxylic acid transmembrane trans...
18. purine ribonucleotide metabolic pro...
19. xenobiotic metabolic process
20. urogenital system development
21. steroid metabolic process
22. gliogenesis
23. glial cell development
24. proton transmembrane transport
25. exocrine system development
26. ornithine metabolic process
27. response to monosaccharide
28. response to thyroid hormone
29. response to nutrient
30. generation of precursor metabolites...
31. salivary gland morphogenesis
32. activation of protein kinase activi...
33. regulation of microvillus organizat...
34. hormone metabolic process
35. positive regulation of fibroblast p...

Figure S9. Trajectory analysis highlights dynamic changes of PT cells towards profibrotic and mesenchymal cell states. Related to Figures 3 and 4.

(a-b) REACTOME "cGMP effects" **(a)** and GOBP "negative regulation of oxidative stress" scores **(b)** in the integrated ZSF1 rat DKD dataset by experimental groups; p values are given for one-way ANOVA (Tukey-corrected).

(c) Corresponding scatterplot for correlation of GOBP "negative regulation of oxidative stress" score (y axis) and REACTOME "cGMP effects" score (x axis); p value is given for linear regression.

(d-e) REACTOME "cGMP effects" **(d)** and GOBP "negative regulation of oxidative stress" scores **(e)** in a human kidney bulk RNA-seq dataset by DKD severity; p values are given for one-way ANOVA (Tukey-corrected).

(f) Corresponding scatterplot for correlation of GOBP "negative regulation of oxidative stress" score (y axis) and REACTOME "cGMP effects" score (x axis); p value is given for linear regression; error bands represent 95% confidence interval.

(g) Expression dot plot for NO/sGC/cGAMP pathway genes in stroma subclusters (rows), corresponding to **Fig. 3e**. Dot size denotes percentage of cells expressing the marker. Color scale represents average gene expression values. Mesang, mesangial cell; JGA, juxtaglomerular apparatus cell; Fib, fibroblast; PT, proximal tubule; GEC, glomerular endothelial cell; Myofib, myofibroblast; VSCM, vascular smooth muscle cell; Peri, pericyte.

(h) Feature plots for *Gucy1b1* and *Gucy1b2* in UMAP space.

(i-j) Monocle3-derived PT-Mesench trajectory, colored by cell clusters along the trajectory **(i)** and by pseudotime **(j)**, respectively. PST, proximal straight tubule; PTinj, injured PT; ProfibPT, profibrotic PT; DediffPT, dedifferentiated PT; Int, interstitial cell; Mesench, mesenchymal cell.

(k) Expression heatmap of top 5 differentially expressed genes (DEGs) along Monocle3-derived pseudotime. Rows represent DEGs, columns represent individual PT cells in bins along pseudotime.

(l) Pearson correlation coefficient (PCC) matrix of top 100 DEG expression between pseudotime bins along Monocle3-derived trajectory.

(m) Corresponding enrichment of top pseudotime-specific GO biological processes and KEGG, Pathway Interaction Database (PID), and HALLMARK pathways, respectively.

(n) Monocle2-derived PT-Mesench trajectory (top) and corresponding heatmap visualizing pseudotiome-dependent gene expression along lineages 1 and 2, respectively. R denotes root node, B denotes branching point, 1 and 2 denote end points of lineages 1 and 2, respectively.

(o-p) Gene ontology (GO) terms derived from top PTinj_1 **(o)** and PTinj_2-specific **(p)** DEGs, corresponding to **Fig. 3e**.

(q) GO terms enriched in PTinj_1 and PTinj_2, respectively, in latent semantic space.

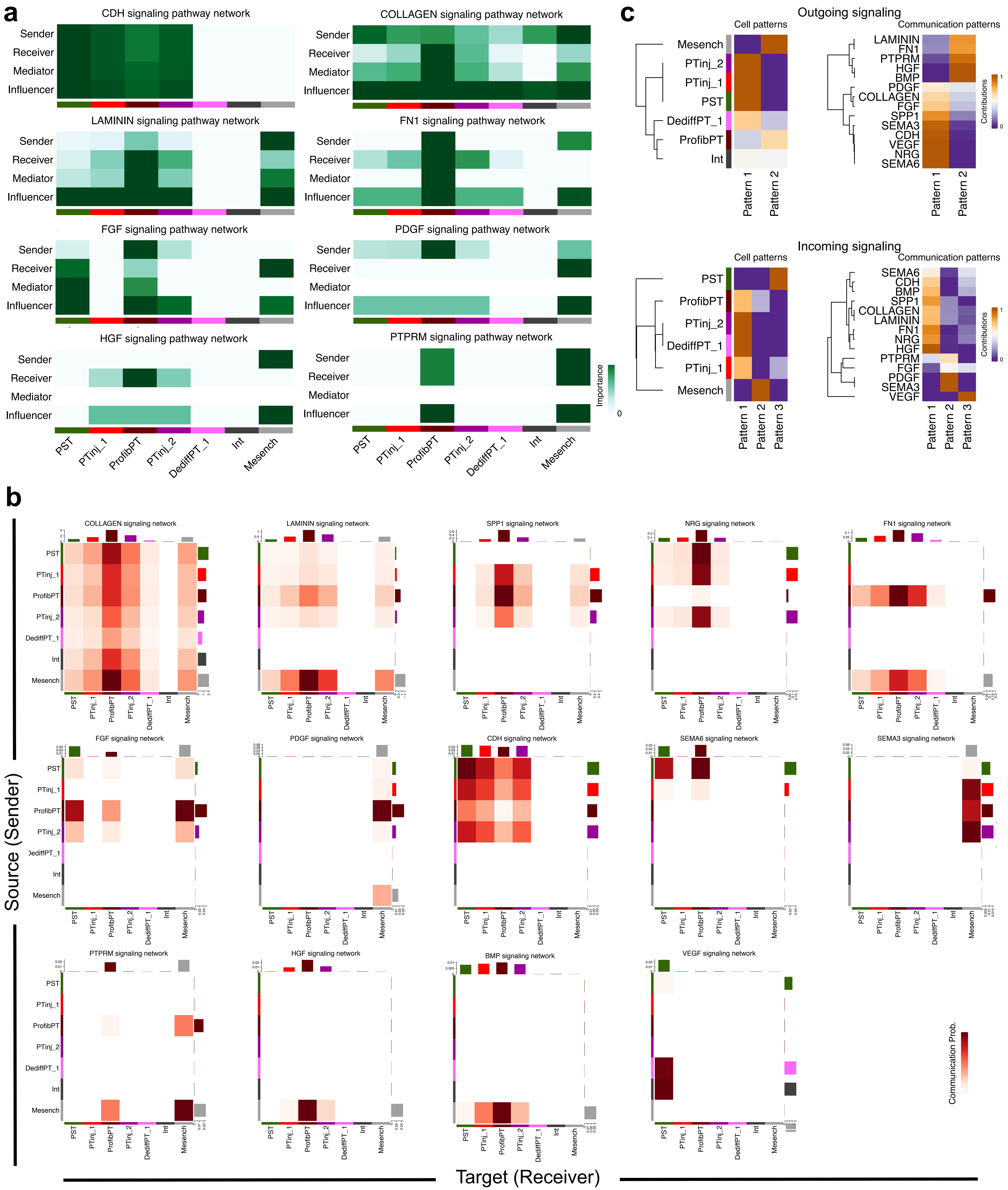
a
CDH signaling pathway network
COLLAGEN signaling pathway network
LAMININ signaling pathway network
FN1 signaling pathway network
FGF signaling pathway network
PDGF signaling pathway network
HGF signaling pathway network
PTPRM signaling pathway network
Sender
Receiver
Mediator
Influencer
Importance
0
PST
PTinj_1
ProfibPT
PTinj_2
DedifPT_1
Int
Mesench
c
Outgoing signaling
Incoming signaling
Cell patterns
Communication patterns
Contributions
Pattern 1
Pattern 2
Pattern 3
LAMININ
FN1
PTPRM
HGF
BMP
PDGF
COLLAGEN
FGF
SPP1
SEMA3
CDH
VEGF
NRG
SEMA6
b
COLLAGEN signaling network
LAMININ signaling network
SPP1 signaling network
NRG signaling network
FN1 signaling network
FGF signaling network
PDGF signaling network
CDH signaling network
SEMA6 signaling network
SEMA3 signaling network
PTPRM signaling network
HGF signaling network
BMP signaling network
VEGF signaling network
Source (Sender)
Target (Receiver)
Communication Prob.

Figure S10. Cell-cell communication analysis identifies a secretory phenotype of profibrotic PT. Related to Figure 5.

(a) Importance of signaling (sender, receiver, mediator, influencer) roles, as computed by CellChat centrality scores, stratified by cell clusters along the PT-Mesench trajectory, corresponding to **Fig. 5**. PST, proximal straight tubule; PTinj, injured PT; ProfibPT, profibrotic PT; DediffPT, dedifferentiated PT; Int, interstitial cell; Mesench, mesenchymal cell.

(b) Communication probability between source/sender (rows) and target/receiver cell clusters (columns) along the PT-Mesench trajectory, displayed individually for various signaling pathways.

(c) Communication patterns for outgoing (top) and incoming (bottom) signaling. Degree of contribution is plotted by cell clusters along the trajectory (left) and by signaling pathways (right), respectively.

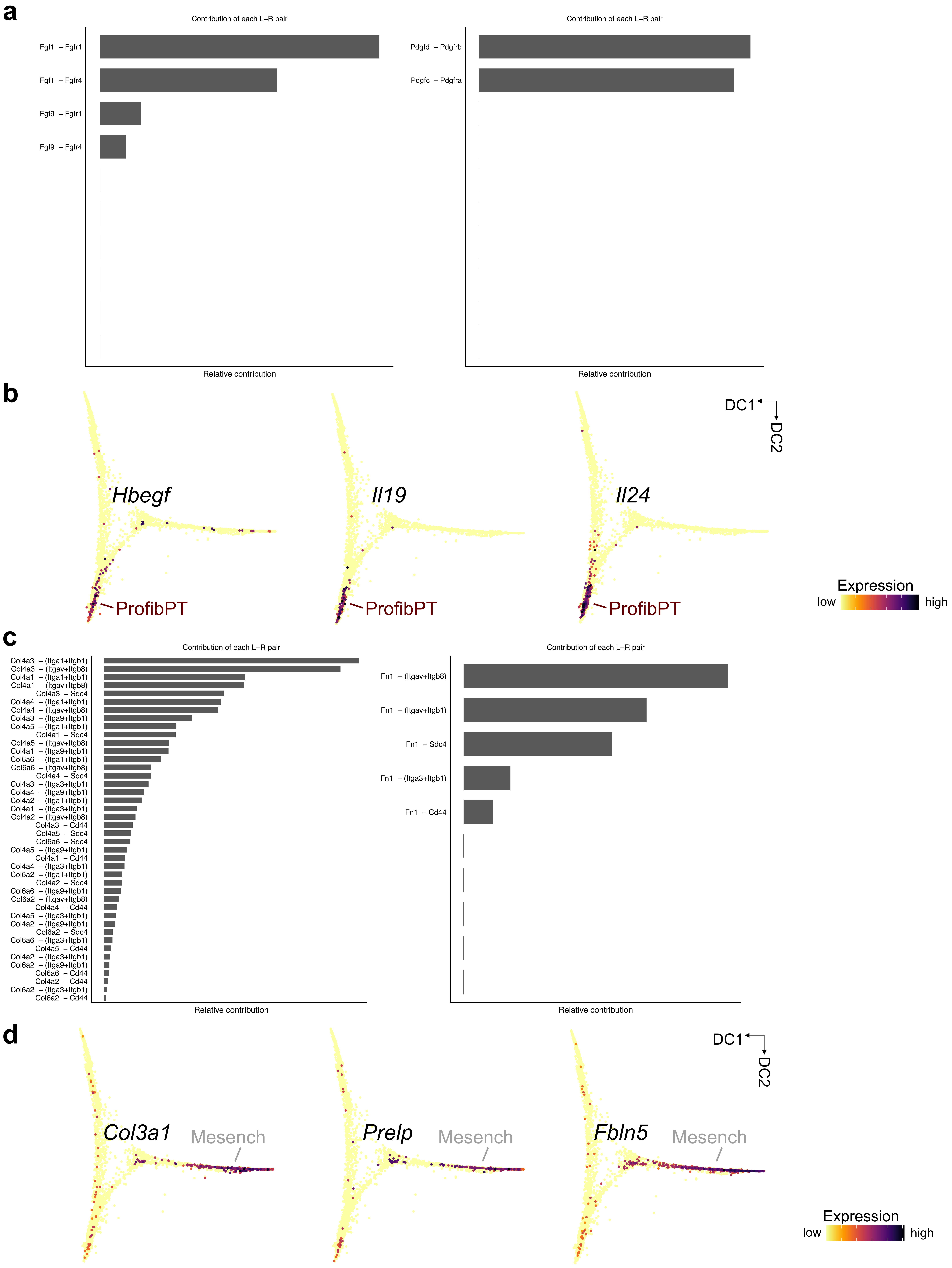
a
Contribution of each L–R pair
Fgf1 – Fgfr1
Fgf1 – Fgfr4
Fgf9 – Fgfr1
Fgf9 – Fgfr4
Relative contribution
Contribution of each L–R pair
Pdgfd – Pdgfrb
Pdgfc – Pdgfra
Relative contribution
b
Hbegf
ProfibPT
Il19
ProfibPT
Il24
ProfibPT
DC1
DC2
Expression
low
high
c
Contribution of each L–R pair
Col4a3 – (Itga1+Itgb1)
Col4a3 – (Itgav+Itgb8)
Col4a1 – (Itga1+Itgb1)
Col4a1 – (Itgav+Itgb8)
Col4a3 – Sdc4
Col4a4 – (Itga1+Itgb1)
Col4a4 – (Itgav+Itgb8)
Col4a3 – (Itga9+Itgb1)
Col4a5 – (Itga1+Itgb1)
Col4a1 – Sdc4
Col4a5 – (Itgav+Itgb8)
Col4a1 – (Itga9+Itgb1)
Col6a6 – (Itga1+Itgb1)
Col6a6 – (Itgav+Itgb8)
Col4a4 – Sdc4
Col4a3 – (Itga3+Itgb1)
Col4a4 – (Itga9+Itgb1)
Col4a2 – (Itga1+Itgb1)
Col4a1 – (Itga3+Itgb1)
Col4a2 – (Itgav+Itgb8)
Col4a3 – Cd44
Col4a5 – Sdc4
Col6a6 – Sdc4
Col4a5 – (Itga9+Itgb1)
Col4a1 – Cd44
Col4a4 – (Itga3+Itgb1)
Col6a2 – (Itga1+Itgb1)
Col4a2 – Sdc4
Col6a6 – (Itga9+Itgb1)
Col6a2 – (Itgav+Itgb8)
Col4a4 – Cd44
Col4a5 – (Itga3+Itgb1)
Col4a2 – (Itga9+Itgb1)
Col6a2 – Sdc4
Col6a6 – (Itga3+Itgb1)
Col4a5 – Cd44
Col4a2 – (Itga3+Itgb1)
Col6a2 – (Itga9+Itgb1)
Col6a6 – Cd44
Col4a2 – Cd44
Col6a2 – (Itga3+Itgb1)
Col6a2 – Cd44
Relative contribution
Contribution of each L–R pair
Fn1 – (Itgav+Itgb8)
Fn1 – (Itgav+Itgb1)
Fn1 – Sdc4
Fn1 – (Itga3+Itgb1)
Fn1 – Cd44
Relative contribution
d
Col3a1
Mesench
Prelp
Mesench
Fbln5
Mesench
DC1
DC2
Expression
low
high

Figure S11. Secretory and ECM-receptor phenotypes of profibrotic PT and mesenchymal cells, respectively. Related to Figure 5.

(a, c) Relative contribution of secreted extracellular matrix (ECM) factors **(a)** and ECM-receptor signaling **(b)**.

(b, d) Feature plots for representative secreted ECM factor genes *(Hbegf, Il19, Il24)* **(b)** and representative core matrisome genes *(Col3a1, Prelp, Fbln5)* **(d)** along the trajectory in diffusion map space, corresponding to **Figs. 5g, j**. ProfibPT, profibrotic PT; Mesench, mesenchymal cell.

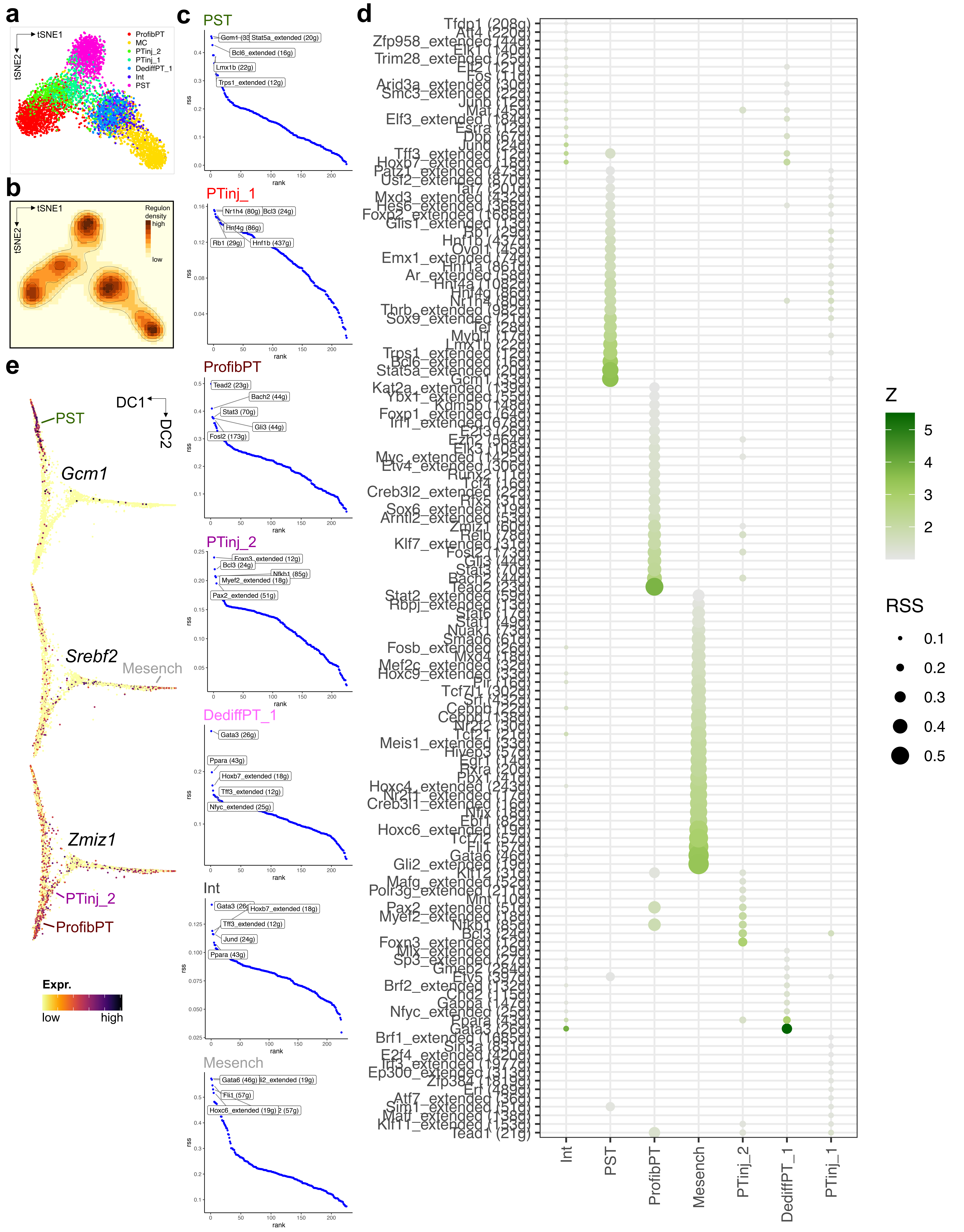
a
tSNE1
tSNE2
ProfibPT
MC
PTinj_2
PTinj_1
DediffPT_1
Int
PST
b
tSNE1
tSNE2
Regulon density
high
low
c
PST
Gcm1 (33
Stat5a_extended (20g)
Bcl6_extended (16g)
Lmx1b (22g)
Trps1_extended (12g)
rss
rank
PTinj_1
Nr1h4 (80g)
Bcl3 (24g)
Hnf4g (86g)
Rb1 (29g)
Hnf1b (437g)
ProfibPT
Tead2 (23g)
Bach2 (44g)
Stat3 (70g)
Gli3 (44g)
Fosl2 (173g)
PTinj_2
Foxn3_extended (12g)
Bcl3 (24g)
Nfkb1 (85g)
Myef2_extended (18g)
Pax2_extended (51g)
DediffPT_1
Gata3 (26g)
Ppara (43g)
Hoxb7_extended (18g)
Tff3_extended (12g)
Nfyc_extended (25g)
Int
Gata3 (26g
Hoxb7_extended (18g)
Tff3_extended (12g)
Jund (24g)
Ppara (43g)
Mesench
Gata6 (46g)
Fli1 (57g)
Hoxc6_extended (19g)
d
Zfp958_extended
Trim28_extended
Arid3a_extended
Elf3_extended
Gli2_extended
Brf2_extended
Nfyc_extended
Int
PST
ProfibPT
Mesench
PTinj_2
DediffPT_1
PTinj_1
Z
5
4
3
2
RSS
0.1
0.2
0.3
0.4
0.5
e
DC1
DC2
PST
Gcm1
Srebf2
Mesench
Zmiz1
PTinj_2
ProfibPT
Expr.
low
high

Figure S12. Gene regulatory network analysis highlights cell type-specific transcription factors driving the PT-to-mesenchymal trajectory and prioritizes cell types of action for sGC modulation. Related to Figure 6.

(a) t-distributed stochastic neighbor embedding (t-SNE) reduction plot of regulon-derived clustering of cell types along the PT-Mesench trajectory. PST, proximal straight tubule; PTinj, injured PT; ProfibPT, profibrotic PT; DediffPT, dedifferentiated PT; Int, interstitial cell; Mesench, mesenchymal cell.

(b) tSNE representation of regulon density as a surrogate for stability of regulon states.

(c) Regulons ranked according to their specificity scores (rss) for individual cell types along the PT-Mesench trajectory. The top 5 specific regulons per cell type are annotated.

(d) Dot plot of regulons (rows) according to cell types along the PT-Mesench trajectory (columns). Dot size denotes regulon specificity score (RSS), color denotes z-scored RSS.

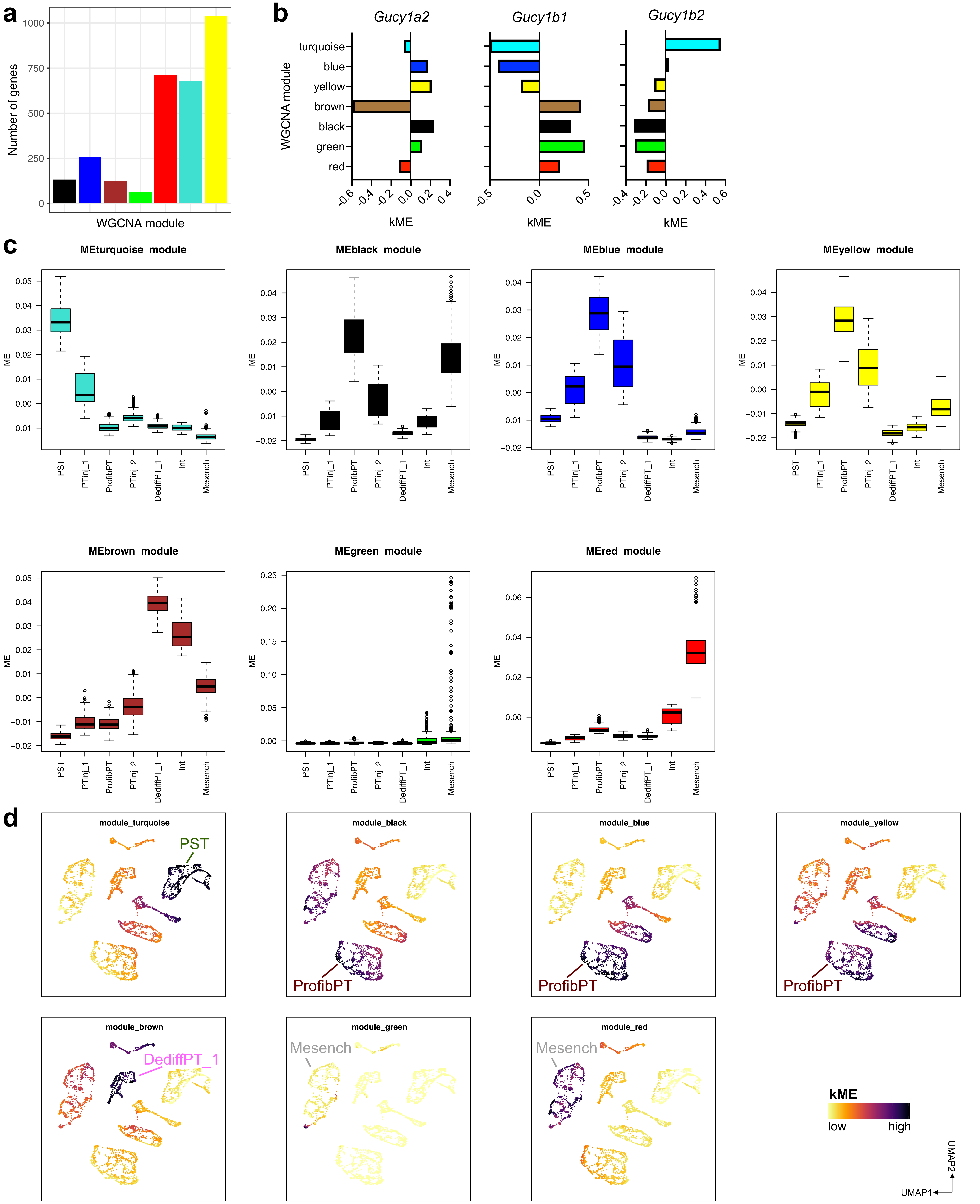

a
Number of genes
WGCNA module
b
Gucy1a2
Gucy1b1
Gucy1b2
WGCNA module
turquoise
blue
yellow
brown
black
green
red
kME
c
MEturquoise module
MEblack module
MEblue module
MEyellow module
MEbrown module
MEgreen module
MEred module
ME
PST
PTinj_1
ProfibPT
PTinj_2
DediffPT_1
Int
Mesench
d
module_turquoise
module_black
module_blue
module_yellow
module_brown
module_green
module_red
PST
ProfibPT
DediffPT_1
Mesench
kME
low
high
UMAP1
UMAP2

Figure S13. WGCNA analysis in PT-Mesench trajectory metanuclei. Related to Figure 7.

(a) Bar graph depicting the number of genes in WGCNA-derived modules.

(b) Intramodular connectivity (kME) values of *Gucy1a2, Gucy1b1*, and *Gucy1b2*.

(c) Module eigengene (ME) scores of WGCNA modules displayed as Tukey box plots (outliers denoted as dots outside box plot whiskers) by cell clusters along the PT-Mesench trajectory (n=3,120 metanuclei). PST, proximal straight tubule; PTinj, injured PT; ProfibPT, profibrotic PT; DediffPT, dedifferentiated PT; Int, interstitial cell; Mesench, mesenchymal cell.

(d) kME values of individual WGCNA modules visualized in UMAP space.

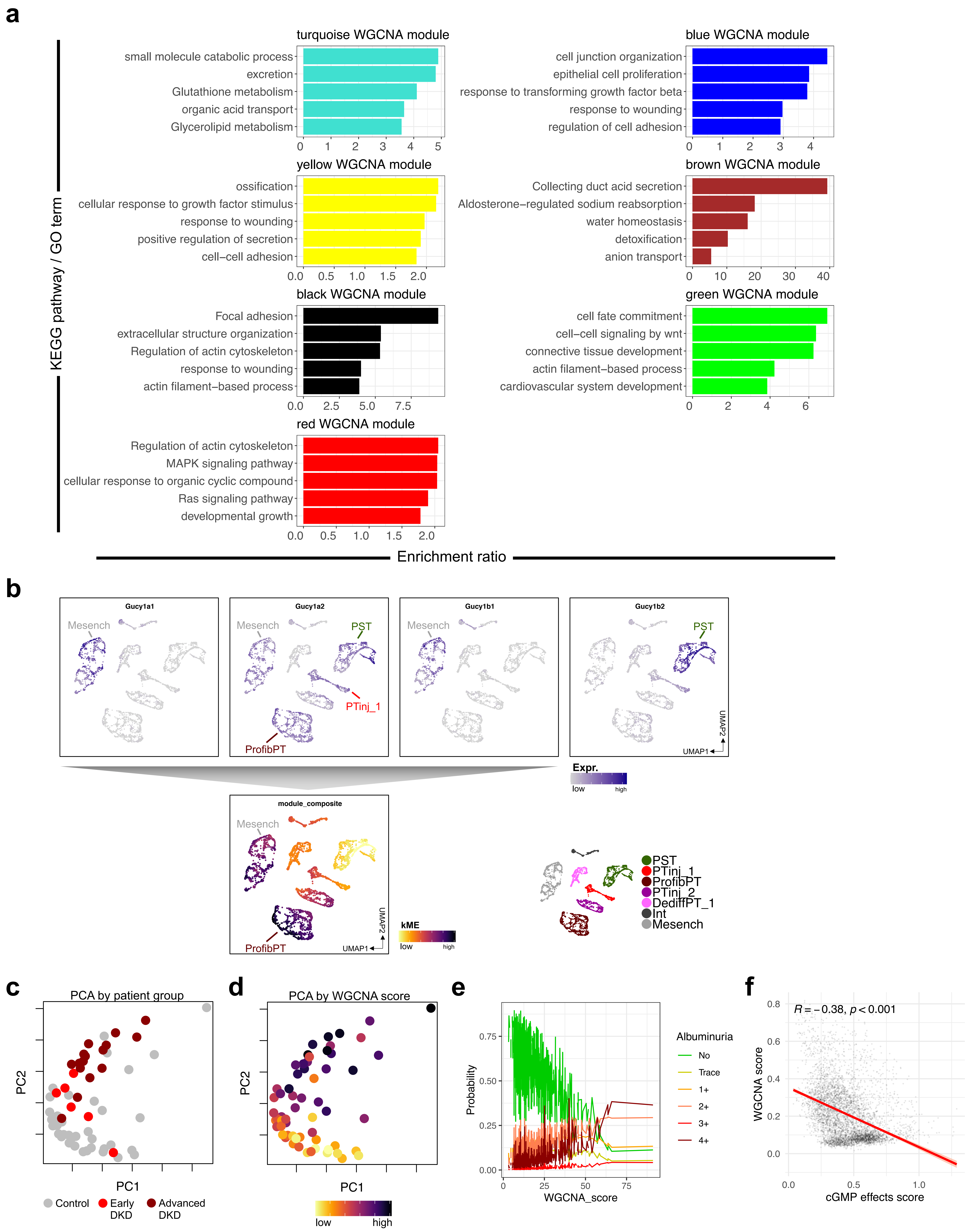

a
KEGG pathway / GO term
turquoise WGCNA module
small molecule catabolic process
excretion
Glutathione metabolism
organic acid transport
Glycerolipid metabolism
blue WGCNA module
cell junction organization
epithelial cell proliferation
response to transforming growth factor beta
response to wounding
regulation of cell adhesion
yellow WGCNA module
ossification
cellular response to growth factor stimulus
response to wounding
positive regulation of secretion
cell–cell adhesion
brown WGCNA module
Collecting duct acid secretion
Aldosterone–regulated sodium reabsorption
water homeostasis
detoxification
anion transport
black WGCNA module
Focal adhesion
extracellular structure organization
Regulation of actin cytoskeleton
response to wounding
actin filament–based process
green WGCNA module
cell fate commitment
cell–cell signaling by wnt
connective tissue development
actin filament–based process
cardiovascular system development
red WGCNA module
Regulation of actin cytoskeleton
MAPK signaling pathway
cellular response to organic cyclic compound
Ras signaling pathway
developmental growth
Enrichment ratio
b
Gucy1a1
Gucy1a2
Gucy1b1
Gucy1b2
Mesench
PST
PTinj_1
ProfibPT
UMAP1
UMAP2
Expr.
low
high
module_composite
kME
PST
PTinj_1
ProfibPT
PTinj_2
DediffPT_1
Int
Mesench
c
PCA by patient group
PC1
PC2
Control
Early DKD
Advanced DKD
d
PCA by WGCNA score
PC1
PC2
low
high
e
Probability
WGCNA_score
Albuminuria
No
Trace
1+
2+
3+
4+
f
$R = -0.38, p < 0.001$
WGCNA score
cGMP effects score

Figure S14. WGCNA-derived sGC co-expression modules correlate with human DKD outcome variables. Related to Figure 7.

(a) Enrichment ratios of top enriched KEGG pathway and GO terms, derived by overrepresentation analysis of WGCNA module genes. The graph is split into subpanels by WGCNA module.

(b) Expression of sGC genes *(Gucy1a1, Gucy1a2, Gucy1b1, Gucy1b2)* (top) and kME values of composite WGCNA score (bottom) visualized in UMAP space. Insert (bottom right) indicates cell types along the PT-Mesench trajectory. PST, proximal straight tubule; PTinj, injured PT; ProfibPT, profibrotic PT; DediffPT, dedifferentiated PT; Int, interstitial cell; Mesench, mesenchymal cell.

(c-d) Principal component analysis (PCA) plots stratifying microdissected kidney tubule RNA-seq samples from human individuals with and without DKD by disease severity **(c)** and by WGCNA score **(d)**.

(e) Ordinal logistic regression showed that as WGCNA score increased, probability of human subjects to have any form of albuminuria increased and probability to be normoalbuminuric decreased.

(f) Scatterplot for correlation of WGCNA score (y axis) and REACTOME "cGMP effects" score (x axis); p value is given for linear regression, error bands represent 95% confidence interval.
